# Synthesis, in silico studies and biological screening of (*E*)-2-(3-(substitutedstyryl)-5-(substitutedphenyl)-4,5-dihydropyrazol-1-yl)benzo[d]thiazole derivatives as an anti-oxidant, anti-inflammatory and antimicrobial agents

**DOI:** 10.1186/s13065-022-00901-2

**Published:** 2022-11-24

**Authors:** Manoj Kumar, Vijay Kumar, Vikramjeet Singh, Samridhi Thakral

**Affiliations:** Department of Pharmaceutical Sciences, Guru Jambheshwar University of Science and Technology, Hisar, 125001 Haryana India

**Keywords:** Dibenzalacetones, Antimicrobial, Anti-inflammatory, DPPH assay, Molecular docking, ADMET

## Abstract

A new series of (*E*)-2-(3-(substitutedstyryl)-5-(substitutedphenyl)-4,5-dihydropyrazol-1-yl)benzo[d]thiazole derivatives was synthesized and the chemical structures of synthesized compounds were deduced by IR and NMR spectral tools. These compounds were synthesized via aldol condensation reaction of substituted benzaldehydes and acetone in alkaline ethanolic solution and their in vitro anti-oxidant, anti-inflammatory and antimicrobial activities were investigated. All the synthesized compounds displayed anti-oxidant potential with IC_50_ values ranging from 0.13 to 8.43 µmol/ml. The compound **Z13** exhibited potent anti-inflammatory activity with IC_50_ value of 0.03 µmol/ml compared with the standard ibuprofen, which showed IC_50_ value of 0.11 µmol/ml. On the other hand, most of the compounds had a certain antibacterial potential particularly against *P. aeruginosa* and among these derivatives, compound **Z2** exhibited the highest potential against *P. aeruginosa* with MIC value of 0.0069 µmol/ml. The analysis of docking results demonstrated the binding affinity and hydrogen bond, electrostatic and hydrophobic interactions of all the synthesized compounds with their respective targets. In silico ADMET studies were carried out for the synthesized compounds and most of the compounds exhibited good ADMET profile.

**Supplementary Information:**

The online version contains supplementary material available at 10.1186/s13065-022-00901-2.

## Introduction

The evolution of medicines, drug discovery, and medicinal chemistry are all intertwined [1]. Medicinal chemistry continues to play an important part in drug discovery, utilising improved methodologies and a better understanding of other fields of related sciences [2]. Dibenzalacetone is an unsaturated organic compound with the chemical formula: C_6_H_5_CH = CHCOCH = CHC_6_H_5_. Dibenzalacetone is also known by the acronym’s DBA and dibenzylideneacetone. It is pale yellow solid in nature that is insoluble in water but generally soluble in alcohol [3]. The IUPAC name of dibenzalacetone is 1,5-diphenylpenta-1,4-dien-3-one. It interacts with metals and aids in the formation of a stable chemical structure and it is employed as a component in sunscreens and some commercial organometallic compounds. It's a symmetrical, non-polar molecule. The dibenzalacetone can show cis–trans geometrical isomerism due to the presence of a double bond. DBA and its analogs can be synthesized via a classic Claisen-Schmidt (cross-aldol) condensation reaction of acetone and benzaldehyde derivatives [4]. Aldol condensation reaction is usually performed to synthesize unsaturated α-carbonyl compounds with various advantages such as chalcone, benzalacetone, etc. [5] Heterocyclic compounds have a cyclic structure in which the ring encompasses two or more distinct groups of atoms. The quantity and diversity of heteroatoms in the rings of known compounds has grown over time, indicating a continuous transition to incorporate the growing domain of heterocyclic systems. The number of conceivable heterocyclic systems is essentially endless since rings may be of any size, from three-member upwards, and heteroatom’s can be drawn in practically any combination from a huge number of elements (though nitrogen, oxygen, and sulphur are still by far the most frequent) [6]. There are a huge number of heterocyclic compounds known, and the number is continually growing. Molecules containing benzothiazole moiety have broad range of biological action, encompassing antiviral [7], antibacterial [8, 9], anti-inflammatory [10], antidiabetic [11], analgesic [12], antioxidant [13, 14], antidepressant [15], anticonvulsant [16], antianginal [17], anticancer [18], immunomodulatory characteristics [19], antihelmintic [20], antimalarial [21], fungicidal [22–24], insecticidal (Melaku et al. [25]) and herbicidal properties [26–28].

Antioxidants have the ability to protect organisms and cells from the damage caused by oxidative stress, and as a result, much studies have been done to investigate this property [29, 30]. There are several mediators that control inflammation, among them the prostaglandins (PGs) which play a key role in the process. PGs are synthesised from arachidonic acid (AA) via the COX enzyme (cyclooxygenase isoenzymes). COX-1 is a constitutive type that protects cells in the GI tract from damage, while COX-2 is an inducible version that increases PG synthesis during inflammation [31]. At therapeutic levels, most non-steroidal anti-inflammatory medications (NSAIDs) suppress both COX-1 and COX-2. Antimicrobials are anticipated as one of the leading kind of chemotherapy in medical history [32]. Antibiotics are antimicrobial substances that are efficacious against bacterial, parasitic and fungal infections [33]. Antibiotic drugs are extensively employed in the treatment and prevention of bacterial infections since they are the representative form of antibacterial agent [1]. Antibiotics are essential in contemporary medicine, and antibiotic resistance is a serious global health problem. Both at the general level and in an individual, the link between drug exposure and antibiotic resistance is unmistakable [34]. Antibiotic resistance can only be mitigated by reducing needless antibiotic use. In order to tackle microbial resistance, there has been an increasing interest in investigating and creating novel antimicrobial agents from diverse sources [35]. As a result, approaches for screening and measuring antimicrobial activity have received more attention. In view of all these facts the present study was undertaken to synthesize and evaluate of (*E*)-2-(3-(substitutedstyryl)-5-(substitutedphenyl)-4,5-dihydropyrazol-1-yl)benzo[d]thiazole derivatives as anti-oxidant, anti-inflammatory and antimicrobial agents.

## Results and discussion

### Chemistry

**Fig. 1:**
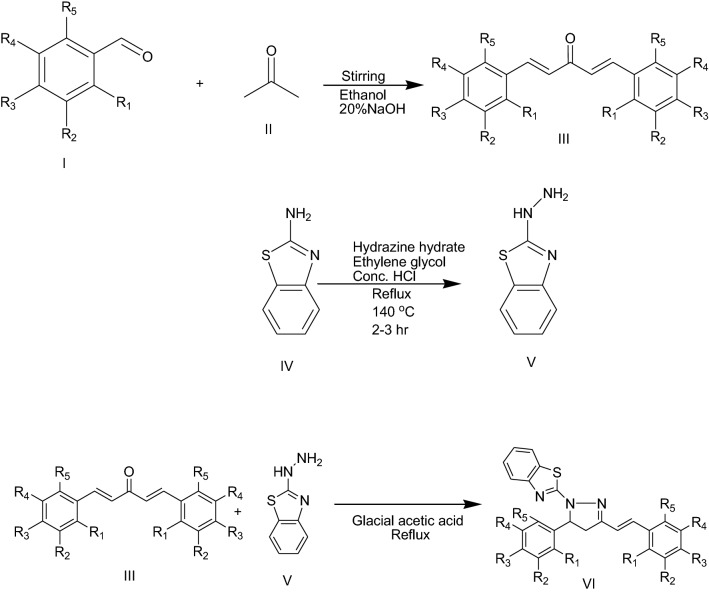
General synthetic scheme for the synthesis of (*E*)-2-(3-(substitutedstyryl)-5-(substitutedphenyl)-4,5-dihydropyrazol-1-yl)benzo[d]thiazole derivatives

The synthesis of (*E*)-2-(3-(substitutedstyryl)-5-(substitutedphenyl)-4,5-dihydropyrazol-1-yl) benzo[d]thiazole derivatives (VI) was accomplished as presented in Fig. 1. The compound (1*E*,4*E*)-1,5-bis(substitutedphenyl)penta-1,4-dien-3-ones (III) was prepared by aldol condensation reaction of substituted benzaldehydes and acetone in alkaline ethanolic solution. From IR spectra, the appearance of peaks at 1651.66 cm^−1^confirmed the presence of α, β unsaturated ketone of synthesized compound III. The aromatic C–H stretching (3027 cm^−1^), aliphatic CH stretching (2950 cm^−1^, 2835 cm^−1^), aromatic C = C stretching (1495 cm^−1^, 1448 cm^−1^), aliphatic C = C stretching (1595 cm^−1^), CH = CH trans (982 cm^−1^) were found in IR spectra of synthesized compound III. 1-(Benzo[d]thiazol-2-yl)hydrazine (V) was synthesized from benzothiazole amine by reaction with hydrazine hydrate in the presence of ethylene glycol. FTIR spectra depicted the presence of NH stretching at 3449 cm^−1^, aromatic CH str. at 3064 cm^−1^, C = N str at 1560 cm^−1^, C–N str at 1282 cm^−1^, C–S-C at 757.92 cm^−1^. (*E*)-2-(3-(Substitutedstyryl)-5-(substitutedphenyl)-4,5-dihydropyrazol-1-yl)benzo[d]thiazole derivative (VI) was synthesized from (1*E*,4*E*)-1,5-bis (substitutedphenyl) penta-1,4-dien-3-one) III and 1-(benzo-[d]thiazol-2-yl)hydrazine (V) in the presence of glacial acetic acid.

The synthesized compounds were characterized by spectral means (FTIR and ^1^HNMR, Additional File 1). The presence of stretching band around 1601–1660 cm^−1^ revealed the existence of C = N functional group. The existence of C–N was demonstrated by the presence of stretching band at 1177–1384 cm^−1^. C–S–C stretching peaks appeared around 750–780 cm^−1^. The FTIR spectrum exhibited characteristics peaks for aromatic CH and aliphatic CH stretching at 3000–3084 cm^−1^ and 2834–2872 cm^−1^, respectively. C–Cl stretching, C–F stretching and C–Br stretching peaks appeared at 692–722 cm^−1^, 1250–1280 cm^−1^and 593–617 cm^−1^, respectively. Presence of methoxy group was confirmed by stretching around 1030–1046 cm^−1^. Hydroxy group stretching peak were observed at 3400–3500 cm^−1^ and NO_2_ group exhibited asymmetrical and symmetrical stretching at 1500–1570 cm^−1^ and 1300–1350 cm^−1^, respectively. ^1^HNMR peaks of H_a_, H_b_ and H_x_ of pyrazole ring appeared at δ 2.13–3.45, 2.39–3.90 and 6.42–5.88 ppm, respectively. The ^1^HNMR spectrum of compounds showed doublet at around δ 6–7 ppm (J = 16 MHz) indicating the ethylene moiety in trans confirmation.

**Table:** 

General structure of target compounds					

Compound	R_1_	R_2_	R_3_	R_4_	R_5_
Z1	H	H	Cl	H	H
Z2	H	Cl	H	H	H
Z3	H	NO_2_	H	H	H
Z4	H	H	NO_2_	H	H
Z5	NO_2_	H	H	H	H
Z6	Cl	H	H	H	H
Z7	H	OH	H	H	H
Z8	H	H	OH	H	H
Z9	H	H	Br	H	H
Z10	H	H	CH_3_	H	H
Z11	H	H	OCH_3_	H	H
Z12	H	H	H	H	H
Z13	H	H	F	H	H
Z14	H	OCH_3_	H	H	H
Z15	OCH_3_	H	H	H	H
Z16	H	Br	H	H	H
Z17	Cl	Cl	H	H	H
Z18	Cl	H	H	H	Cl
Z19	OCH_3_	H	H	OCH_3_	H
Z20	H	OCH_3_	OCH_3_	OCH_3_	H

### In vitro biological evaluation

#### Anti-oxidant activity

All the synthesized compounds were evaluated for anti-oxidant activity via DPPH assay method. Compound **Z1** (R = 4-Cl) showed maximum anti-oxidant potential with IC_50_ value of 0.13 µmol/ml (85.54 ± 0.22% inhibition at 500 µg/ml) in comparison to standard compound (0.50 µmol/ml) as presented in Table 1. Compound **Z13** (R = 4-F) also displayed higher anti-oxidant activity with IC_50_ value of 0.44 µmol/ml. Compounds **Z16** (R = 3-Br), **Z11** (R = 4-OCH_3_), **Z3** (R = 3-NO_2_) were observed as least active compounds amongst the synthesized compounds with IC_50_ values of 8.43, 4.80 and 2.03 µmol/ml, respectively.

**Table 1:** Anti-oxidant activity (µmol/ml) of synthesized (*E*)-2-(3-(substitutedstyryl)-5-(substitutedphenyl)-4,5-dihydropyrazol-1-yl)benzo[d]thiazole derivatives (VI)

Comp	Conc	%Inhibition ± SEM	IC_50_	Comp	Conc	%Inhibition ± SEM	IC_50_
Z1	500 250 125 62.5 31.25	85.54 ± 0.22^**^ 76.10 ± 0.04^**^ 62.54 ± 0.29^**^ 49.84 ± 0.05^**^ 35.33 ± 0.13^**^	0.13	Z11	500 250 125 62.5 31.25	51.75 ± 0.12^**^ 40.67 ± 0.04^**^ 31.30 ± 0.05^**^ 29.17 ± 0.02^**^ 24.56 ± 0.03^**^	4.8
Z2	500 250 125 62.5 31.25	96.89 ± 0.02 ^**^ 71.08 ± 0.02^**^ 50.46 ± 0.29^**^ 30.57 ± 0.03^**^ 21.72 ± 0.02^**^	0.71	Z12	500 250 125 62.5 31.25	86.50 ± 0.21^**^ 62.82 ± 0.09^**^ 48.05 ± 0.01^**^ 40.12 ± 0.03^**^ 36.96 ± 0.02^**^	1.68
Z3	500 250 125 62.5 31.25	72.67 ± 0.22^**^ 52.30 ± 0.00^**^ 37.38 ± 0.02^**^ 29.39 ± 0.01^**^ 23.70 ± 0.07^**^	2.03	Z13	500 250 125 62.5 31.25	56.07 ± 0.02^**^ 48.56 ± 0.02^**^ 36.70 ± 0.04^**^ 29.54 ± 0.09^**^ 24.50 ± 0.01^**^	0.44
Z4	500 250 125 62.5 31.25	68.26 ± 0.07^**^ 53.70 ± 0.09^**^ 39.35 ± 0.06^**^ 28.11 ± 1.05^**^ 22.01 ± 0.00^**^	0.61	Z14	500 250 125 62.5 31.25	53.00 ± 0.94^**^ 39.13 ± 0.34^**^ 29.63 ± 0.00^**^ 22.58 ± 0.00^**^ 18.54 ± 0.01^**^	5.41
Z5	500 250 125 62.5 31.25	70.82 ± 0.4^**^ 57.59 ± 0.07^**^ 45.80 ± 0.04^**^ 39.61 ± 0.30^**^ 34.40 ± 0.18^**^	1.05	Z15	500 250 125 62.5 31.25	55.37 ± 0.11^**^ 43.49 ± 0.13^**^ 32.89 ± 0.03^**^ 28.20 ± 0.04^**^ 24.37 ± 0.00^**^	0.86
Z6	500 250 125 62.5 31.25	28.96 ± 0.03^**^ 25.85 ± 0.03^**^ 22.16 ± 0.00^**^ 19.99 ± 0.00^**^ 17.53 ± 0.02^**^	0.74	Z16	500 250 125 62.5 31.25	68.22 ± 0.00^**^ 54.48 ± 0.01^**^ 43.37 ± 0.00^**^ 37.93 ± 0.00^**^ 32.39 ± 0.01^**^	8.43
Z7	500 250 125 62.5 31.25	86.39 ± 0.04^**^ 65.10 ± 0.00^**^ 50.44 ± 0.00^**^ 30.83 ± 0.01^**^ 23.81 ± 0.02^**^	0.71	Z17	500 250 125 62.5 31.25	39.80 ± 0.33^**^ 34.61 ± 0.18^**^ 26.75 ± 0.04^**^ 23.96 ± 0.03^**^ 18.91 ± 0.04^**^	0.54
Z8	500 250 125 62.5 31.25	83.87 ± 0.01^**^ 64.66 ± 0.07^**^ 27.30 ± 0.02^**^ 21.53 ± 0.00^**^ 19.83 ± 0.16^**^	0.50	Z18	500 250 125 62.5 31.25	86.11 ± 0.3^**^ 49.05 ± 0.00^**^ 31.67 ± 0.05^**^ 26.63 ± 0.01^**^ 23.13 ± 0.01^**^	1.75
Z9	500 250 125 62.5 31.25	64.36 ± 0.03^**^ 49.79 ± 0.00^**^ 34.62 ± 0.01^**^ 23.35 ± 0.00^**^ 12.81 ± 0.02^**^	0.70	Z19	500 250 125 62.5 31.25	50.57 ± 0.1^**^ 38.53 ± 0.31^**^ 28.53 ± 0.07^**^ 22.92 ± 0.00^**^ 21.84 ± 0.04^**^	0.58
Z10	500 250 125 62.5 31.25	61.12 ± 0.06^**^ 47.88 ± 0.00^**^ 36.72 ± 0.00^**^ 26.70 ± 0.03^**^ 20.95 ± 0.01^**^	0.87	Z20	500 250 125 62.5 31.25	52.64 ± 0.01^**^ 42.45 ± 0.01^**^ 32.53 ± 0.01^**^ 26.87 ± 0.01^**^ 21.62 ± 0.04^**^	0.88
				STD	500 250 125 62.5 31.25	99.921 ± 0.03 88.951 ± 0.00 74.337 ± 0.24 55.319 ± 0.30 40.186 ± 0.51	0.50

This data is represented as Mean ± SEM, n = 3, values are significantly different as compared to positive control (STD) Ascorbic acid (500 µg/ml) (^**^P < 0.01)

#### Anti-inflammatory activity

All the synthesized compounds [(*E*)-2-(3-(substitutedstyryl)-5-(substitutedphenyl)-4,5-dihydropyrazol-1-yl)benzo[d]thiazole derivatives] were evaluated for their in vitro anti-inflammatory potential by egg albumin assay method as presented in Table 2. Compound **Z13** (R = 4-F) was found to be most potent compound with IC_50_ value of 0.03 µmol/ml (79.26 ± 0.13% inhibition at 500 µg/ml) as compared to standard compound ibuprofen (IC_50_ = 0.11 µmol/ml). Compound **Z3** (R = 3-NO_2_) gave 2^nd^ highest activity with IC_50_ value of 0.05 µmol/ml. Compound **Z8** (R = 4-OH) and **Z10** (R = 4-CH_3_) showed less inhibitory potential with IC_50_ value 3.08 and 1.30 µmol/ml, respectively in comparison with other synthesized compounds.

**Table 2:** Anti-inflammatory activity (µmol/ml) of synthesized (*E*)-2-(3-(substitutedstyryl)-5-(substitutedphenyl)-4,5-dihydropyrazol-1-yl)benzo[d]thiazole derivatives (VI)

Comp	Conc	%Inhibition ± SEM	IC_50_	Comp	Conc	%Inhibition ± SEM	IC_50_
Z1	500 250 125 62.5 31.25	46.38 ± 0.19^**^ 42.52 ± 0.06^**^ 38.07 ± 0.43^**^ 33.51 ± 0.25^**^ 31.69 ± 0.11^**^	0.54	Z11	500 250 125 62.5 31.25	31.00 ± 0.00^**^ 28.72 ± 0.11^**^ 26.26 ± 0.06^**^ 21.92 ± 0.02^**^ 18.47 ± 0.07^**^	0.16
Z2	500 250 125 62.5 31.25	70.42 ± 0.07 ns 58.88 ± 0.07^**^ 40.54 ± 3.07^**^ 33.39 ± 0.01^**^ 27.21 ± 0.07^**^	0.41	Z12	500 250 125 62.5 31.25	82.53 ± 0.28^**^ 75.28 ± 0.06^**^ 62.96 ± 0.01^**^ 54.98 ± 0.29^**^ 43.97 ± 0.38^**^	0.23
Z3	500 250 125 62.5 31.25	71.11 ± 0.03^ ns^ 65.23 ± 0.05^**^ 57.86 ± 0.05^**^ 46.90 ± 0.02^**^ 34.71 ± 0.00^**^	0.05	Z13	500 250 125 62.5 31.25	79.26 ± 0.13^**^ 77.17 ± 0.03^**^ 73.51 ± 0.04^**^ 63.28 ± 0.04^**^ 42.12 ± 0.00^**^	0.03
Z4	500 250 125 62.5 31.25	42.26 ± 0.00^**^ 38.37 ± 0.00^**^ 35.91 ± 0.02^**^ 29.49 ± 0.00^**^ 25.14 ± 0.00^**^	0.13	Z14	500 250 125 62.5 31.25	29.58 ± 0.17^**^ 26.98 ± 0.29^**^ 24.54 ± 0.33^**^ 20.04 ± 0.10^**^ 16.73 ± 0.09^**^	0.17
Z5	500 250 125 62.5 31.25	44.16 ± 0.07^**^ 41.06 ± 0.01^**^ 36.32 ± 0.07^**^ 32.92 ± 0.01^**^ 29.15 ± 0.02^**^	0.27	Z15	500 250 125 62.5 31.25	39.57 ± 0.33^**^ 36.83 ± 0.00^**^ 34.62 ± 0.00^**^ 29.58 ± 0.00^**^ 26.53 ± 0.01^**^	0.19
Z6	500 250 125 62.5 31.25	77.70 ± 0.32^**^ 62.26 ± 0.09^**^ 42.24 ± 0.19^**^ 28.67 ± 0.07^**^ 22.17 ± 0.34^**^	0.42	Z16	500 250 125 62.5 31.25	57.24 ± 0.07^**^ 45.39 ± 0.14^**^ 37.15 ± 0.10^**^ 28.20 ± 0.15^**^ 24.08 ± 0.05^**^	1.20
Z7	500 250 125 62.5 31.25	50.23 ± 0.09^**^ 42.92 ± 0.02^**^ 37.68 ± 0.25^**^ 26.65 ± 0.13^**^ 22.15 ± 0.03^**^	0.28	Z17	500 250 125 62.5 31.25	71.05 ± 0.03^ ns^ 60.72 ± 0.14^**^ 51.87 ± 0.27^**^ 43.38 ± 0.01^**^ 38.31 ± 0.35^**^	0.61
Z8	500 250 125 62.5 31.25	48.57 ± 1.34^**^ 35.98 ± 0.06^**^ 27.95 ± 0.02^**^ 20.48 ± 1.33^**^ 17.30 ± 0.79^**^	3.08	Z18	500 250 125 62.5 31.25	51.07 ± 0.00^**^ 46.05 ± 0.05^**^ 37.66 ± 0.01^**^ 35.63 ± 0.01^**^ 31.12 ± 0.01^**^	0.43
Z9	500 250 125 62.5 31.25	86.05 ± 0.02^**^ 64.45 ± 0.02^**^ 49.63 ± 0.09^**^ 33.12 ± 0.03^**^ 26.53 ± 0.04^**^	0.90	Z19	500 250 125 62.5 31.25	58.11 ± 0.02^**^ 54.56 ± 0.11^**^ 48.08 ± 0.05^**^ 36.74 ± 0.31^**^ 26.95 ± 0.63^**^	0.14
Z10	500 250 125 62.5 31.25	80.14 ± 0.02^**^ 55.13 ± 0.05^**^ 38.64 ± 0.03^**^ 28.13 ± 0.05^**^ 24.72 ± 0.08^**^	1.30	Z20	500 250 125 62.5 31.25	32.22 ± 0.02^**^ 30.19 ± 0.04^**^ 27.25 ± 0.00^**^ 24.24 ± 0.02^**^ 21.28 ± 0.01^**^	0.15
				STD	500 250 125 62.5 31.25	70.53 ± 0.03 69.11 ± 0.01 66.53 ± 0.03 60.46 ± 0.03 50.55 ± 0.14	0.11

This data is represented as Mean ± SEM, n = 3, values are significantly different as compared to positive control (STD) Ibuprofen (500 µg/ml) (^**^P < 0.01); ns- non-significant

#### Antimicrobial activity

The synthesized derivatives were tested against Gram positive *B. subtilis* (MTCC 441), *S. aureus* (MTCC 3160), and Gram negative *E. coli* (MTCC 16,521), *P. aeruginosa* (MTCC 647) for antibacterial activity and *C. albicans* (MTCC 183) and *R. oryzae* (MTCC 262) for antifungal activity by serial dilution method. Compound **Z6** (R = 2-Cl) exhibited most potent antibacterial activity against *B. subtilis* with MIC value of 0.0069 µmol/ml as compared to ciprofloxacin (0.0075 µmol/ml). Compound **Z13** (R = 4-F) was found as the second most active compound against *B. subtilis* with MIC value of 0.0150 µmol/ml. Compound **Z14** (R = 3-OCH_3_), **Z5** (R = 2-NO_2_) and **Z12** (R = H) were observed as the least active compounds and showed antibacterial activity against *B. subtilis* with MIC values of 0.0566, 0.0530, 0.0328 µmol/ml, respectively.

Compounds **Z17** (R = 2,3-diCl) and **Z18** (R = 2,6-diCl) among the synthesized compounds showed good antibacterial activity against *E. coli* with MIC value of 0.0241 µmol/ml. Compounds **Z8** (R = 4-OH), **Z10** (R = 4-CH_3_), **Z11** (R = 4-OCH_3_), **Z14** (R = 3-OCH_3_) and **Z15** (R = 2-OCH_3_) were found to be least active compounds against *E. coli*. Compounds **Z20** (R = 3,4,5-tri-OCH_3_), **Z9** (R = 4-Br) and **Z16** (R = 3-Br) displayed good antibacterial activity against *S. aureus* with MIC values of 0.0223, 0.0232 and 0.0232 µmol/ml, respectively. Compound **Z14** (R = 3-OCH_3_) was found as least active compound. Compound **Z2** (R = 3-Cl) revealed maximum inhibitory potential against *P. aeruginosa* with MIC value of 0.0069 µmol/ml. Compounds **Z11** (R = 4-OCH_3_), **Z15** (R = 2-OCH_3_), **Z13** (R = 4-F), **Z12** (R = H) also showed good antibacterial activity against *P. aeruginosa* with MIC values of 0.0140, 0.0140, 0.0149 and 0.063 µmol/ml, respectively. Compounds **Z14** (R = 3-OCH_3_) and **Z10** (R = 4-CH_3_) showed minimum inhibitory potential against *P. aeruginosa*.

In case of Gram positive bacterial strain, study indicated that compound **Z20** (R = 3,4,5-tri-OCH_3_) showed better antibacterial potential towards both *B. subtilis* and *S. aureus* with MIC value of 0.223 µmol/ml. Compounds **Z14** (R = 3-OCH_3_) and **Z12** (R = H) were found to have minimum inhibitory potential against Gram positive bacterial strains. In case of Gram negative strains synthesized compounds such as **Z2** (R = 3-Cl), **Z11** (R = 4-OCH_3_), **Z15** (R = 2-OCH_3_), **Z13** (R = 4-F), **Z12** (R = H) showed maximum inhibitory potential against *P. aeruginosa*. Compounds **Z9** (R = 4-Br), **Z16** (R = 3-Br), **Z20** (R = 3,4,5-tri-OCH_3_) exhibited good antifungal potential against both fungal strains.

Compounds **Z9** (R = 4-Br), **Z16** (R = 3-Br) and **Z20** (R = 3,4,5-tri-OCH_3_), demonstrated good antifungal activity against *C. albicans* with MIC values of 0.223, 0.0232 and 0.232 µmol/ml. Compounds **Z14** (R = 3-OCH_3_) and **Z17** (R = 2,3-di-Cl) displayed minimum inhibitory potential against *C. albicans*. Compounds **Z7** (R = 3-OH), **Z20** (R = 3,4,5-tri-OCH_3_) displayed maximum antifungal activity against *R. oryzae* with MIC values of 0.0151 and 0.0223 µmol/ml. Compounds **Z12** (R = H) and **Z10** (R = 4-CH_3_) were observed as least active compound against *R. oryzae*. The antimicrobial study revealed that the synthesized (*E*)-2-(3-(substitutedstyryl)-5-(substitutedphenyl)-4,5-dihydropyrazol-1-yl)benzo[d]thiazole derivatives exhibited most potent antibacterial potential against *P. aeruginosa* as shown in Table 3.

**Table 3:** Antimicrobial activity (µmol/ml) of synthesized (*E*)-2-(3-(substitutedstyryl)-5-(substitutedphenyl)-4,5-dihydropyrazol-1-yl)benzo[d]thiazole derivatives

Comp	*B. subtilis*	*S. aureus*	*E. coli*	*P. aeruginosa*	*C. albicans*	*R. oryzae*
Z1	0.0278	0.0278	0.0278	0.0278	0.0278	0.0278
Z2	0.0278	0.0278	0.0555	0.0069	0.0278	0.0278
Z3	0.0265	0.0265	0.0265	0.0265	0.0265	0.0265
Z4	0.0265	0.0265	0.0530	0.0265	0.0265	0.0265
Z5	0.0530	0.0265	0.0530	0.0265	0.0265	0.0265
Z6	0.0069	0.0278	0.0555	0.0278	0.0278	0.0278
Z7	0.0302	0.0302	0.0302	0.0302	0.0302	0.0151
Z8	0.0302	0.0302	0.0605	0.0302	0.0302	0.0302
Z9	0.0232	0.0232	0.0464	0.0232	0.0232	0.0232
Z10	0.0305	0.0305	0.0610	0.0305	0.0305	0.0305
Z11	0.0283	0.0283	0.0566	0.0142	0.0283	0.0283
Z12	0.0328	0.0328	0.0328	0.0164	0.0328	0.0328
Z13	0.0150	0.0299	0.0299	0.0150	0.0299	0.0299
Z14	0.0566	0.0566	0.0566	0.0566	0.0566	0.0283
Z15	0.0283	0.0283	0.0566	0.0142	0.0283	0.0283
Z16	0.0232	0.0232	0.0464	0.0232	0.0232	0.0232
Z17	0.0241	0.0241	0.0241	0.0241	0.0481	0.0241
Z18	0.0241	0.0241	0.0241	0.0241	0.0241	0.0241
Z19	0.0249	0.0249	0.0498	0.0249	0.0249	0.0249
Z20	0.0223	0.0223	0.0445	0.0223	0.0223	0.0223
STD^a^	0.0075	0.0075	0.0075	0.0075	0.0040	0.0040

^a^Ciprofloxacin (antibacterial), Fluconazole (antifungal)

**Fig. 2:**
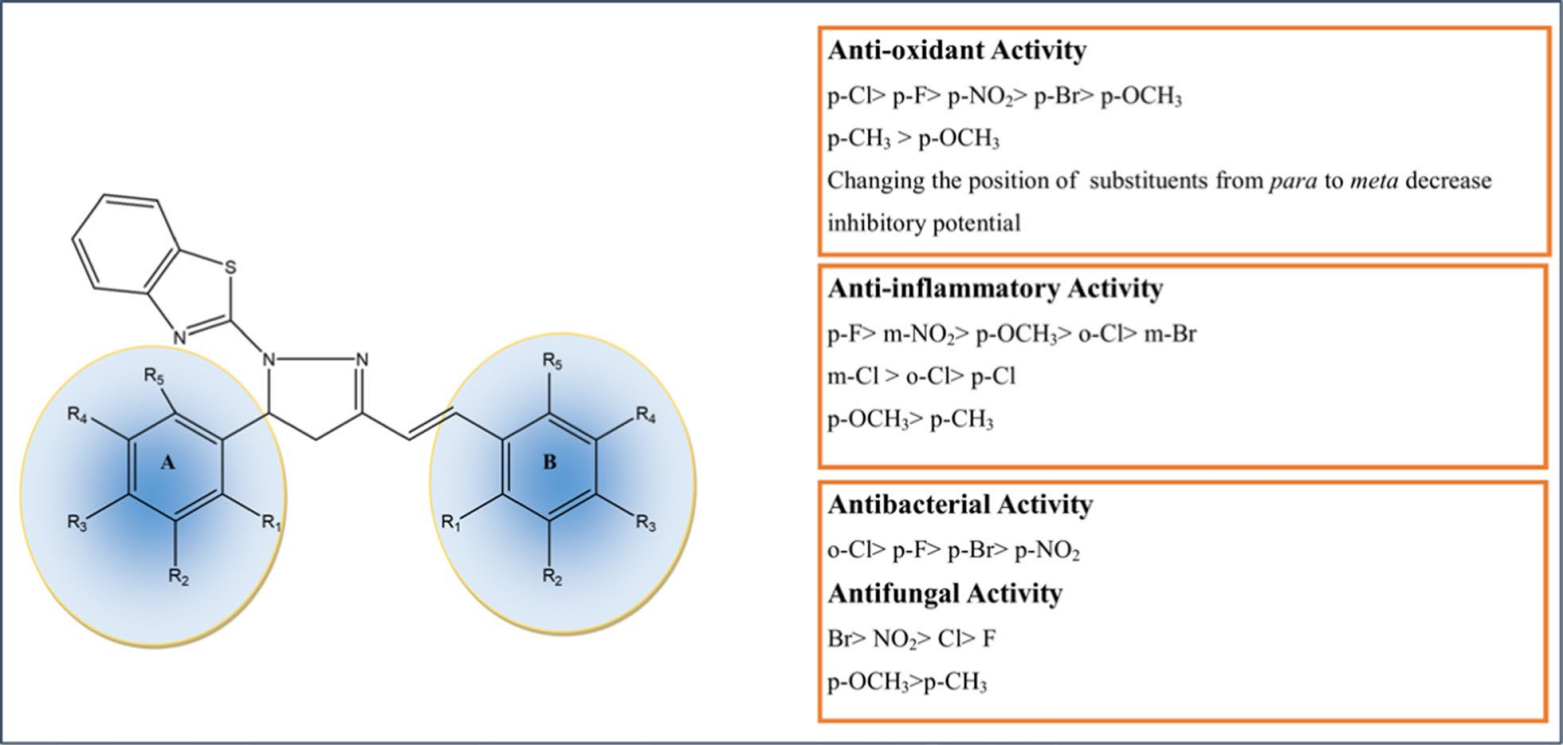
Structure activity relationships of synthesized (E)-2-(3-(substitutedstyryl)-5-(substitutedphenyl)-4,5-dihydropyrazol-1-yl)benzo[d]thiazole derivatives

Structure Activity Relationship can be summarized as follows: (Fig. 2)

### Molecular docking

**Fig. 3:**
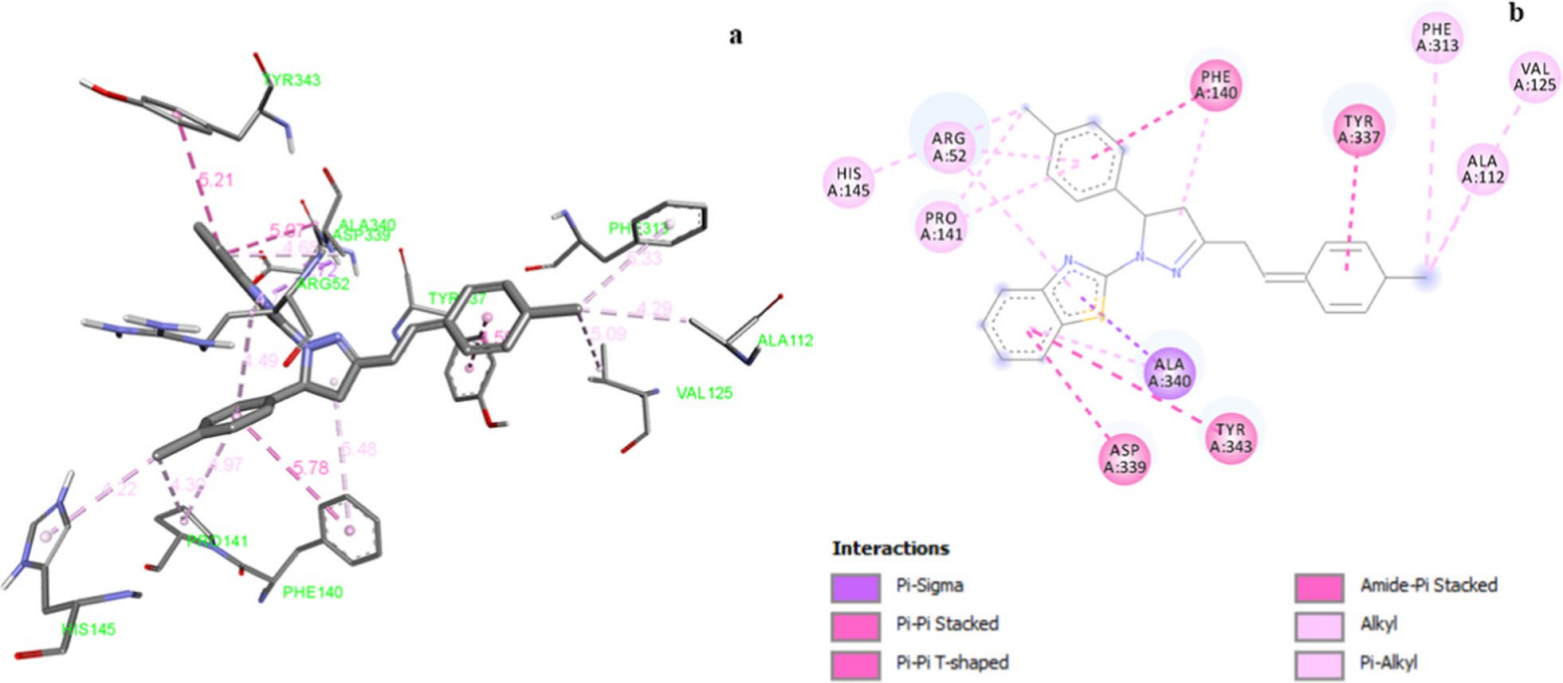
**a** 3D illustration of compound **Z1** in the active site of catalase enzyme (PDB:2CAG). **b** 2D illustration of compound **Z1** in the active site of catalase enzyme (PDB:2CAG)

Molecular docking is appraised as remarkable tool to explore the binding affinity and binding interactions of synthesized compounds with active binding sites of corresponding proteins. In the present study all the synthesized compounds were subjected to molecular docking studies with respect to their target proteins. Binding score of all the synthesized compound against target protein PDB:2CAG is represented in Table 4. Compound **Z1** exhibited hydrophobic interactions with amino acid residues of marked protein PDB: 2CAG (Catalase compound II) (Fig. 3). *Para* chlorostyryl ring created pi-pi stacked interaction with Tyr:337 (4.59 Å) residue. Phenyl ring of benzothiazole displayed pi-pi stacked interaction with Tyr:343 (5.21 Å) and amide-pi-stacked interaction with AspA:339 (5.07 Å) amino acid residue. Pi-pi T shaped interaction was induced by *para* chloro substituted phenyl ring with Phe:140 amino acid residue with bond length of 5.78 Å. Alkyl and pi-alkyl interactions were observed with Val:125, Ala:112, Pro:141, Phe:140, Pro:141, His:145, Arg:52, Ala:340 and Phe:313 amino acid residues. Thiazole ring of benzothiazole was engaged in pi-sigma interaction with Ala:340 amino acid residue. Compound **Z16** displayed four pi-pi stacked and three pi-alkyl and one alkyl interaction with target residue. Compound **Z11** the second least active compound created two hydrogen bonds, one pi-pi stacked, alkyl and pi-alkyl interactions with target residue. Ascorbic acid displayed three hydrogen bond and one pi-donor hydrogen bond interaction with PheA:313, AlaA:311, ArgA:344 and HisA:54 amino acid residues of the target protein.

**Table 4:** Binding affinity of synthesized (*E*)-2-(3-(substitutedstyryl)-5-(substitutedphenyl)-4,5-dihydropyrazol-1-yl)benzo[d]thiazole derivatives against its respective targets (Kcal/mol)

Comp	PDB:2CAG	PDB:6COX	PDB: 1U4G	PDB:1EA1
Z1	− 9.8	− 8.6	− 7.8	− 10.5
Z2	− 11.3	− 10.0	− 8.7	− 10.4
Z3	− 9.2	− 10.8	− 9.2	− 11.3
Z4	− 10.8	− 9.0	− 8.8	− 10.6
Z5	− 10.5	− 9.7	− 7.9	− 10.4
Z6	− 9.1	− 9.2	− 9.3	− 10.6
Z7	− 9.9	− 11.1	− 8.1	− 9.9
Z8	− 10.5	− 9.1	− 8.6	− 10.5
Z9	− 10.1	− 8.8	− 7.9	− 10.0
Z10	− 9.9	− 10.7	− 8.8	− 10.7
Z11	− 9.2	− 8.1	− 7.7	− 10.0
Z12	− 9.2	− 9.4	− 7.6	− 10.4
Z13	− 10.6	− 10.0	− 8.2	− 11.0
Z14	− 10.6	− 10.3	− 9.0	− 10.4
Z15	− 9.8	− 9.0	− 8.0	− 10.6
Z16	− 8.3	− 9.1	− 9.1	− 10.5
Z17	− 9.2	− 9.1	− 7.9	− 10.9
Z18	− 9.4	− 8.7	− 7.6	− 10.6
Z19	− 7.8	− 8.4	− 7.2	− 10.0
Z20	− 9.0	− 7.4	− 6.9	− 10.0
STD	− 6.1	− 7.2	− 6.6	− 7.2

PDB:2CAG (Catalase compound II); PDB:6COX (Cyclooxygenase-2 (prostaglandin synthase-2) complexed with a selective inhibitor, SC-558 IN I222 space group); PDB:1U4G (Elastase of *P. aeruginosa* with an inhibitor); PDB: 1EA1 (cytochrome P450 14 alpha-sterol demethylase (CYP51) from *Mycobacterium tuberculosis* in complex with fluconazole)

**Fig. 4:**
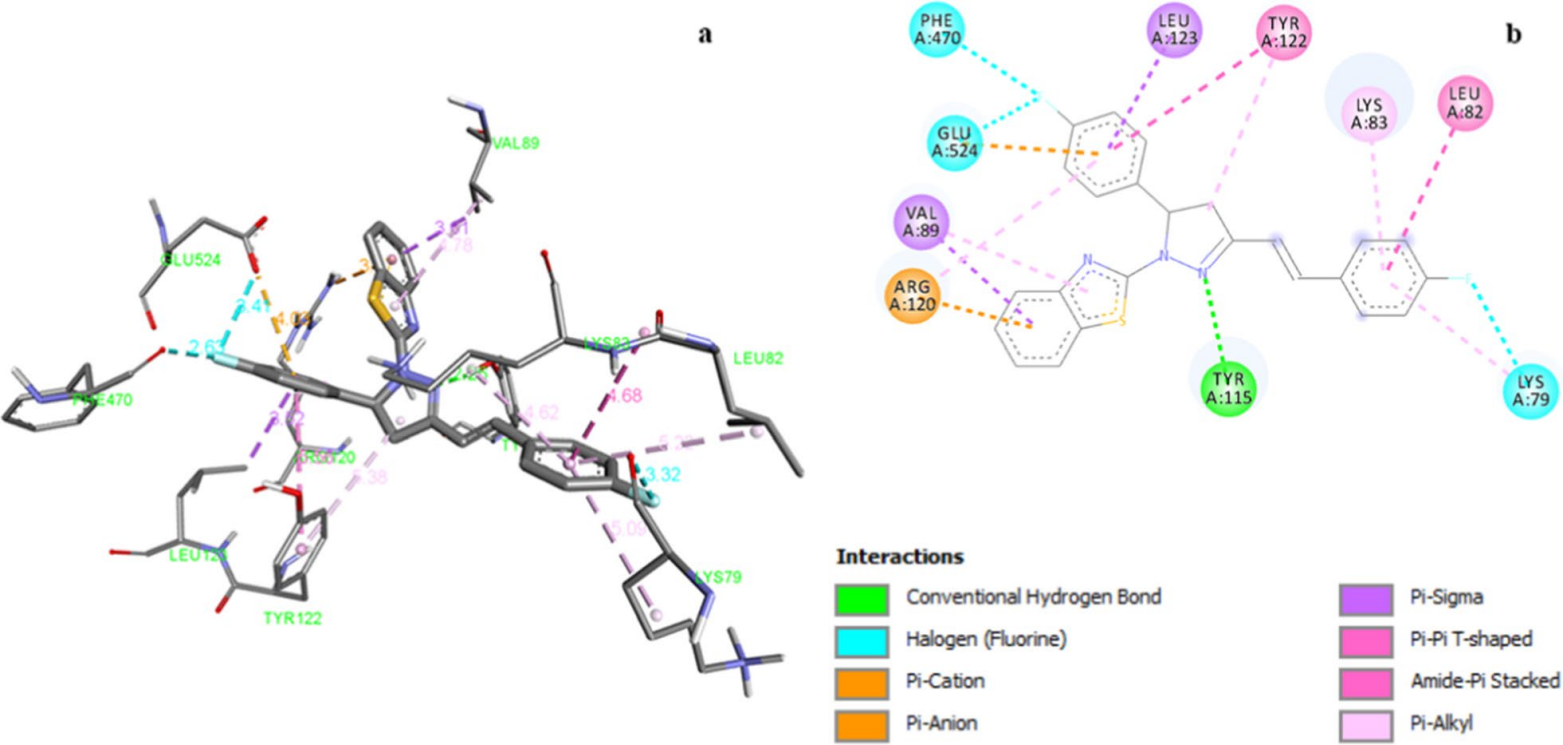
**a** 3D illustration of compound **Z13** in the active site of cyclooxygenase-2 enzyme (PDB:6COX). **b** 2D illustration of compound **Z13** in the active site of cyclooxygenase-2 enzyme (PDB:6COX)

All the synthesized compounds showed binding affinity values ranging between −7.8 to −11.3 kcal/mol against target protein PDB:6COX (Table 4). Compound **Z13** exhibited hydrogen bonding, hydrophobic (pi-pi-T-shaped, amide-pi-stacked, pi-sigma, pi-alkyl), electrostatic (pi-cation, pi-anion) and halogen bond interactions with target residues of PDB:6COX [Cyclooxygenase-2 (prostaglandin synthase 2)] (Fig. 4). TyrA:115 amino acid was involved in hydrogen bond interaction with N of pyrazoline ring at a distance of 2.26 Å. The phenyl ring of benzothiazole contributed pi-cation interaction with Arg:120 amino acid residue (3.37 Å). *Para* fluoro substituted phenyl ring established pi-anion interaction with GluA:254 residue (4.03 Å). Amide-pi-stacked interaction was introduced by LeuA:82 amino acid residue with *para* fluorostyryl ring (4.68 Å) whereas *para* fluoro substituted phenyl ring fascinated pi-pi-T shaped interaction with TyrA:122 amino acid residue with bond length of 5.56 Å. *Para* fluorostyryl ring and *para* fluoro substituted phenyl ring also prompted pi-sigma interactions with ValA:89 and LeuA:123 amino acid residue. LysA:79, LeuA:82, LysA:83, ValA:89, TyrA:122 and ArgA:120 amino acid residues induced pi-alkyl interactions with compound **Z13**. The second least active compound **Z8** displayed lesser interactions such as two hydrogen bonds, one pi-cation, two pi-sigma, one alkyl and one pi-alkyl interaction with target residues. The least active compound **Z10** showed one pi-cation and five pi-alkyl interactions with target residues. The reference compound ibuprofen showed one hydrogen bond, one pi-sigma, alkyl and pi-alkyl interactions with target residues i.e. MetA:522, ValA:523, AlaA:516, HisA:90, ArgA:513 and LeuA:352.

**Fig. 5:**
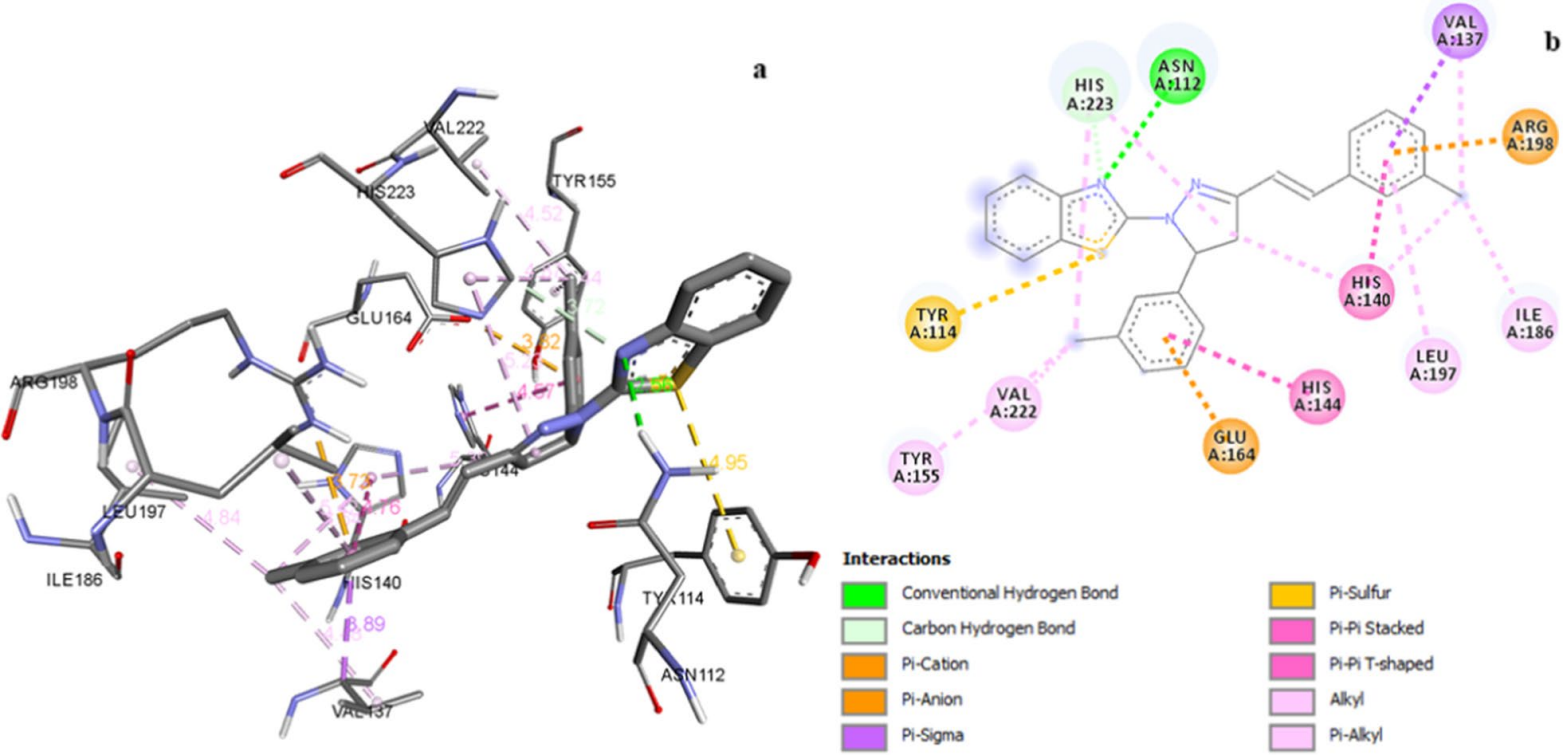
**a** 3D illustration of compound **Z2** in the active site of elastase of *P. aeruginosa* (PDB:1U4G). **b** 2D illustration of compound **Z2** in the active site of elastase of *P. aeruginosa* (PDB:1U4G)

In case of antibacterial activity, all the synthesized compounds exhibited binding affinity in the range of -6.9 to -9.3 kcal/mol (Table 4). In compound **Z2**, nitrogen of benzothiazole ring established hydrogen bond interaction with AsnA:112 amino acid residue at a distance of 2.56 Å (Fig. 5). *Meta* chlorostyryl ring created pi-cation interaction with ArgA:198 whereas *meta* chloro substituted phenyl ring displayed pi-anion interaction with GluA:164 residue. Pi-pi-T shaped interaction was formed by *meta* chlorostyryl ring (4.76 Å) with HisA:140 amino acid residue and *meta* chloro substituted phenyl ring showed pi-pi stacked interaction with HisA:144 (4.57 Å) amino acid residue. The chloro group of synthesized compounds interacted with target protein (ValA:222, IleA:186, ValA:137) via alkyl interaction. Pi-alkyl interactions exhibited by compound **Z2** with LeuA:197, HisA:140, HisA:223 and TyrA:155 amino acid residue. *Meta* chlorostyryl ring was also engaged in pi-sigma interaction with ValA:137 amino acid residue of target protein. The least active compound **Z14** showed two hydrogen bond, three pi-pi-T shaped, three pi-alkyl and one pi-sigma interaction with target residues. The reference compound ciprofloxacin showed three hydrogen bond, one pi-anion, one pi-pi-T shaped, two pi-pi stacked, one alkyl, two pi-alkyl and one halogen bond with target residues like ValA:222, HisA:223, GluA:164, TrpA:115, TyrA:155, HisA:144 and GluA:148.

**Fig. 6:**
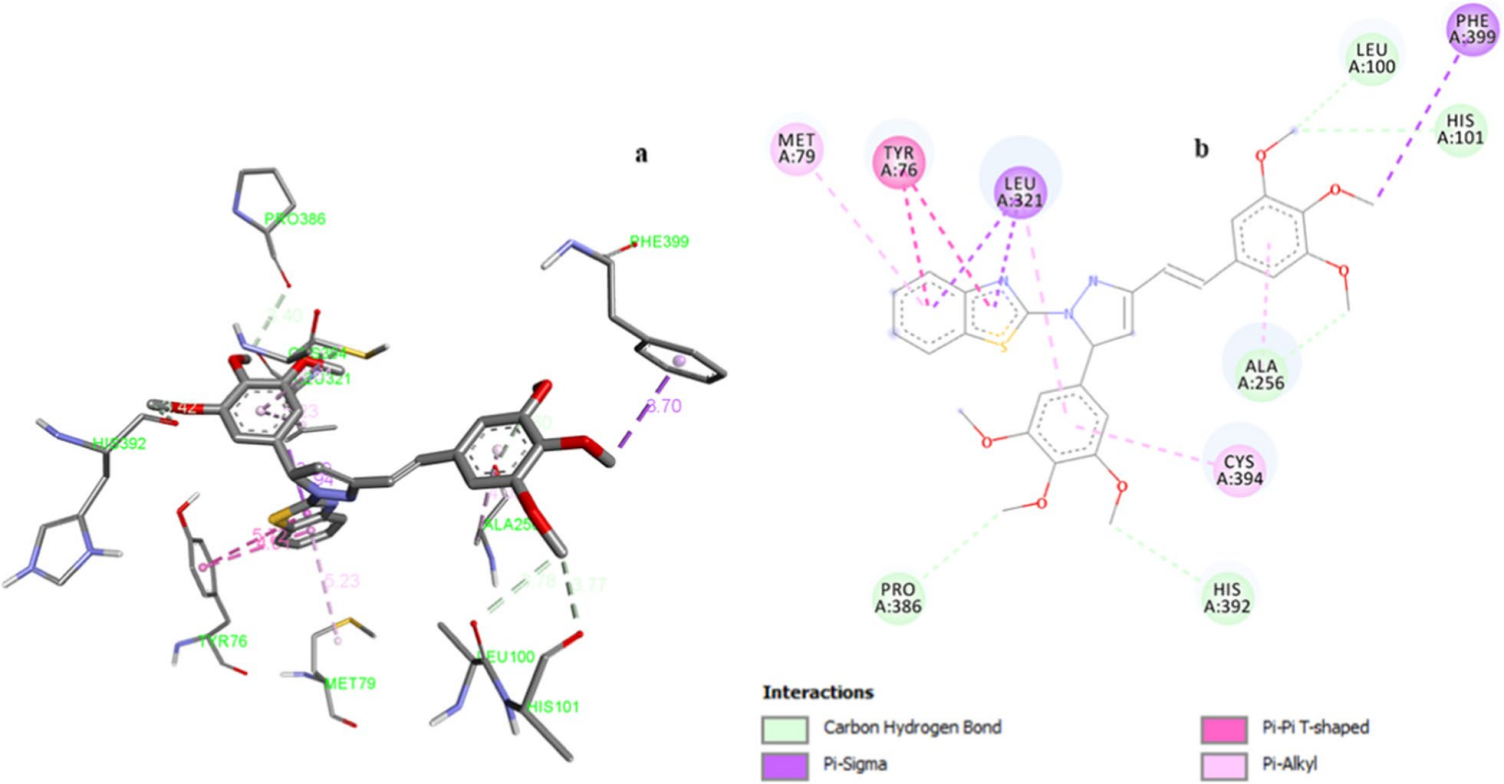
**a** 3D illustrations of compound **Z20** in the active site of cytochrome P450 14alpha-sterol demethylase enzyme (PDB:1EA1). **b** 2D illustrations of compound **Z20** in the active site of cytochrome P450 14alpha-sterol demethylase enzyme (PDB:1EA1)

Molecular docking studies for fungal studies depicted the binding affinity ranging from -9.9 to 11.3 kcal/mol (Table 4) and binding interactions of all the synthesized compounds with target protein PDB:1EA1. Compound **Z20** created hydrophobic and carbon hydrogen bond interactions with target amino acid residues (Fig. 6). The benzothiazole ring was engaged in two pi-pi-T shaped interactions with Tyr:76 amino acid residue with bond length of 4.91 and 5.782 Å. Two pi-sigma interactions were formed by benzothiazole ring at a distance of 3.59 and 3.94 Å. The carbon of methoxy group of trimethoxystyryl ring formed pi-sigma interaction with PheA:399 amino acid residue with bond length of 3.70 Å. Pi-alkyl interactions were created by compound **Z20** with Cys:394, Ala:256, Leu:321 and Met79 amino acid residues. Carbon-hydrogen bond were induced with ProA:386, HisA:392, AlaA:256, HisA:101 and LeuA:100 amino acid residues. The second active compound **Z9** displayed hydrogen bond interaction, pi-pi T shaped, amide-pi-stacked, pi-sigma interactions with Arg:96, Tyr:76, Phe:387, Leu:321, Cys:394, Leu:105, Ala:256, Leu:321 and Met79 target residues. The least active compound **Z14** displayed one pi-pi-T shaped, two alkyl, two pi-sigma, five pi-alkyl interactions with target residues. The standard drug fluconazole exhibited two hydrogen bond, two pi-pi-T shaped, one pi-sigma, one pi-cation, pi-alkyl and one halogen bond interaction with target residues.

### Drug likeness parameters

Molinspiration online tool kit and OSIRIS property explorer was used for the evaluation of drug like characteristics. According to the laws, molecular weight < 500 Daltons, hydrogen bond donors < 5 and hydrogen bond acceptors < 10 and log P not be higher than 5 [36]. If more than two criteria are violated then these rules highlight possible bioavailability problem. The intestinal absorption, oral bioavailability, and blood brain barrier penetration of the drug molecules are influenced by optimum value of descriptor like polar surface area. The compounds with TPSA value < 140 Å^2^ possess better intestinal absorption, molecules with a polar surface area > 140 Å^2^ be likely to be poor at permeating cell membrane and TPSA of < 60 Å^2^ signifies sufficient bioavailability and generally the compounds penetrate the blood brain barrier. The results presented in Table 5 depicted that compounds **Z1**, **Z2**, **Z3**, **Z6**, **Z7**, **Z9** and **Z13** met all the rules of Lipinski. Agreeing to Veber’s rule there must be number of rotatable bonds 10 or < 10 and TPSA equal to or < 140Å^2^ which is also supporting the synthesized compounds [37, 38].

**Table 5:** Drug likeness characteristics of synthesized (*E*)-2-(3-(substitutedstyryl)-5-(substitutedphenyl)-4,5-dihydropyrazol-1-yl)benzo[d]thiazole derivatives

Comp	miLog P^a^	Log S^b^ (mol/L)	TPSA^c^ (Å^2^)	MW^d^	nON^e^	nOHNH^f^	nvoilatio^g^	nrot^h^
Z1	4.01	− 7.13	27.97	454.43	3	0	0	4
Z2	4.88	− 7.13	28.49	450.39	3	0	0	4
Z3	4.44	− 6.582	120.14	471.50	9	0	0	6
Z4	6.49	− 6.582	120.14	471.50	9	0	1	6
Z5	6.21	− 6.582	120.14	471.50	9	0	1	6
Z6	4.65	− 7.134	28.49	450.39	3	0	0	4
Z7	4.57	− 5.07	68.95	413.50	5	2	0	4
Z8	5.61	− 5.07	68.95	413.50	5	2	1	4
Z9	4.18	− 7.33	28.49	539.30	3	0	1	4
Z10	7.47	− 6.35	28.49	409.56	3	0	1	4
Z11	6.69	− 5.698	46.96	441.56	5	0	1	6
Z12	6.57	− 5.662	28.49	381.50	3	0	1	4
Z13	3.90	− 6.29	28.49	417.48	3	0	0	4
Z14	4.43	− 5.698	46.96	441.56	5	0	1	6
Z15	6.41	− 5.698	46.96	441.56	5	0	1	6
Z16	8.13	− 7.33	28.49	539.30	3	0	2	4
Z17	8.65	− 8.606	28.49	519.28	3	0	2	4
Z18	8.65	− 8.606	28.49	519.28	3	0	2	4
Z19	6.48	− 5.734	65.43	501.61	7	0	2	8
Z20	5.83	− 5.77	83.90	561.66	9	0	2	10

^a^*miLog P* Logarithm of partition coefficient between n-octanol and water

^b^*LogS* Solublity

^c^*TPSA* Topological polar surface area

^d^*MW* Molecular weight

^e^*nON* Number of hydrogen bond acceptor

^f^*nOHNH* Number of hydrogen bond donor

^g^*nvoilations* Number of violations

^h^*nrot* Number of rotatable bonds

### ADMET study

Pre-ADMET online server was utilized for estimation of pharmacokinetic parameters. The HIA value ranging 0 and 20% specifies poor absorption, 20–70% moderate absorption, and 70–100% indicates well absorption. In case of Caco-2 cell permeability, the value < 4 shows low permeability, 4–70 moderate permeability, and > 70 high permeability. MDCK cell system may be used as a sensible tool for rapid permeability screening and the value < 25 indicates low permeability, 25–500 moderate permeability, and > 500 high permeability. The percentage of drug bind to plasma protein is another remarkable factor, the value < 90% indicates weak binding and > 90% indicates strong binding to plasma proteins. The blood–brain barrier (BBB) penetration is symbolized as BB = (Brain)/(Blood). The value < 0.1 indicates low absorption, 0.1–2.0 moderate absorption, and > 2.0 higher absorption to CNS [38].

The human intestinal absorption values were observed in range of 96.02–98.48% which recognised as the absorption capacity of synthesized compounds. The in vitro Caco-2 cell permeable property in the range of 1.01–57.80 nm/s, in vitro MDCK cell permeability in range of 0.02–63.42 nm/s designated low to moderate permeability of target compounds with the concerned cell line. The synthesized compounds displayed values in range of 90.12–100% which assured its strong binding capacity with proteins. The in vivo blood brain barrier penetration ranges from 0.32 to 3.62 facilitated its distribution in vivo with medium to good penetration capacity (Table 6) [38].

**Table 6:** ADME analysis of synthesized (*E*)-2-(3-(substitutedstyryl)-5-(substitutedphenyl)-4,5-dihydropyrazol-1-yl)benzo[d]thiazole derivatives by Pre ADMET online server

Comp	Human intestinal absorption (HIA, %)	In vitro Caco-2 cell permeability (nm/s)	In vitro MDCK cell permeability (nm/s)	In vitro plasma protein binding (%)	In vivo blood brain barrier penetration (C.brain/C. blood)	Pgp_inhibition
Z1	98.23	57.80	0.177	100	0.41	Inhibitor
Z2	98.23	57.03	28.85	97.62	0.82	Inhibitor
Z3	98.48	01.82	0.04	93.74	0.32	Inhibitor
Z4	98.48	01.08	0.04	94.60	0.52	Inhibitor
Z5	98.48	01.01	0.04	93.74	0.57	Inhibitor
Z6	98.23	56.83	12.79	97.67	0.51	Inhibitor
Z7	96.02	29.91	0.19	95.10	0.59	Inhibitor
Z8	96.02	39.58	0.04	95.28	0.44	Inhibitor
Z9	98.27	56.27	0.02	100	0.41	Inhibitor
Z10	97.95	38.08	0.37	93.08	0.80	Inhibitor
Z11	97.68	44.87	0.04	91.56	1.97	Inhibitor
Z12	97.86	37.63	63.42	94.17	2.18	Inhibitor
Z13	97.87	53.83	0.05	96.94	0.46	Inhibitor
Z14	97.68	34.80	1.32	91.32	3.62	Inhibitor
Z15	97.68	36.02	0.05	91.64	2.02	Inhibitor
Z16	98.35	56.16	0.02	100	0.74	Inhibitor
Z17	98.37	56.16	0.05	100	0.60	Inhibitor
Z18	98.37	56.64	0.07	100	0.60	Inhibitor
Z19	97.69	33.42	0.04	90.39	2.81	Inhibitor
Z20	98.27	36.86	0.04	90.12	2.41	Inhibitor

Caco-2- Cells derived from human colon adenocarcinomas; MDCK- Medin-Darbey Canine Kidney Epithelial Cells; Pgp- P- glycoprotein (plasma membrane protein)

### Bioactivity and toxicity risk

The bioactivity and toxicity risks of synthesized compounds were estimated by Molinspiration online server and Osiris property explorer, respectively (Table 7).

**Table 7:** Bioactivity and toxicity risks of synthesized (*E*)-2-(3-(substitutedstyryl)-5-(substitutedphenyl)-4,5-dihydropyrazol-1-yl)benzo[d]thiazole derivatives

Comp	GPCR ligand	Ion channel modulator	Kinase inhibitor	Nuclear receptor ligand	Protease inhibitor	Enzyme inhibitor	Mutagenic	Tumorigenic	Reproductive effective	Irritant
Z1	− 0.22	− 0.33	− 0.51	− 0.67	− 0.38	− *0.16*	None	None	None	None
Z2	− 0.37	− 0.48	− 0.57	− 0.53	− 0.56	− 0.31	None	None	None	None
Z3	− 0.46	− 0.48	− 0.58	− 0.53	− 0.58	− 0.35	None	None	None	None
Z4	− 0.45	− 0.48	− 0.59	− 0.53	− 0.58	− 0.34	None	None	None	None
Z5	− 0.43	− 0.45	− 0.69	− 0.52	− 0.67	− 0.36	None	None	None	None
Z6	− 0.40	− 0.53	− 0.73	− 0.49	− 0.62	− 0.37	None	None	None	None
Z7	− 0.34	− 0.46	− 0.52	− 0.39	− 0.52	− 0.24	None	None	None	None
Z8	− 0.34	− 0.45	− 0.51	− 0.40	− 0.52	− 0.24	None	None	None	None
Z9	− 0.46	− 0.55	− 0.58	− 0.61	− 0.62	− 0.34	None	None	None	None
Z10	− 0.41	− 0.55	− 0.58	− 0.54	− 0.57	− 0.33	None	None	None	None
Z11	− 0.39	− 0.52	− 0.54	− 0.49	− 0.54	− 0.30	None	None	None	None
Z12	− 0.40	− 0.52	− 0.58	− 0.55	− 0.56	− 0.30	None	None	None	None
Z13	− 0.37	− 0.50	− 0.52	− 0.49	− 0.55	− 0.29	None	None	None	None
Z14	− 0.39	− 0.53	− 0.55	− 0.49	− 0.55	− 0.31	None	None	None	None
Z15	− 0.40	− 0.53	− 0.57	− 0.51	− 0.57	− 0.32	None	None	None	None
Z16	− 0.47	− 0.56	− 0.60	− 0.62	− 0.64	− 0.35	None	None	None	None
Z17	− 0.37	− 0.47	− 0.71	− 0.46	− 0.58	− 0.35	None	None	None	None
Z18	− 0.43	− 0.53	− 0.59	− 0.42	− 0.53	− 0.32	None	None	None	None
Z19	− 0.37	− 0.51	− 0.53	− 0.44	− 0.52	− 0.29	None	None	None	None
Z20	− 0.33	− 0.65	− 0.49	− 0.52	− 0.47	− 0.32	None	None	None	None

*GPCR ligand*: G-Protein coupled receptor ligand property

## Conclusion

(*E*)-2-(3-(Substitutedstyryl)-5-(substitutedphenyl)-4,5-dihydropyrazol-1-yl)benzo[d]thiazole derivatives were synthesized and evaluated for their anti-oxidant, anti-inflammatory and antimicrobial potential. Compound **Z1** showed the maximum anti-oxidant potential and exhibited hydrophobic interactions with target residues of respective protein. Compound **Z13** was observed as the most potent anti-inflammatory compound and established hydrogen bond, electrostatic, halogen and hydrophobic interactions with amino acid residues of target protein. Compound **Z2** revealed maximum inhibitory potential against *P. aeruginosa* and formed hydrogen bond, hydrophobic and electrostatic interaction with target protein. Compound **Z20** showed good antifungal activity and binding interactions with target residues. Molecular docking studies and pharmacokinetic analysis also supported the in vitro results.

## Materials and methods

### Chemical and instruments

The analytical grade chemicals and reagents were utilized by itself in experiments without any purification. Decibel melting point apparatus was adapted for monitoring the melting point of the synthesized compounds and are expressed as uncorrected. The thin-layer chromatography (TLC) was fascinated for observing the reaction progress. FT-IR (Diffuse Reflectance Method (DRS) -8000A, Shimadzu, Japan) spectrophotometer was utilized for recording infrared spectra and the Bruker Avance III, 400 MHz NMR spectrometer was employed for nuclear magnetic resonance spectra (^1^H NMR, ^13^C NMR, Chemical shift δ values- ppm). DPPH (High Media), Nutrient broth and Sabouard dextrose broth (Hi-Media) have been used for in vitro biological studies.

### General procedure for synthesis of (E)-2-(3-(substitutedstyryl)-5-(substitutedphenyl)-4,5-dihydropyrazol-1-yl)benzo[d]thiazole derivatives (Z1-Z20)

#### Synthesis of (1E,4E)-1,5-bis (substitutedphenyl)penta-1,4-dien-3-one (III)

First of all, 40 mmol benzaldehyde (II) was taken in a round bottom flask and 20 ml of ethanol was added. After dissolution, 20 mmol acetone (I) was added in above mixture. The solution was vigorously stirred for 15 min on magnetic stirrer. RBF was placed in an ice bath for maintaining temperature 1–4 ºC and 20 ml of a freshly prepared 20% sodium hydroxide solution was added drop by drop into the solution with continuous stirring. After complete addition of 20% sodium hydroxide solution, the resulting mixture was continuously stirred for 1 h. The resultant product was neutralized by 10% HCl solution (approximately 50–70 ml). After neutralization the separated product was filtered, washed with water and then dried at room temperature [4].

#### Synthesis of 1-(benzo[d]thiazol-2-yl)hydrazine (V)

1.5 ml of hydrazine hydrate (99%) was taken in a 50 ml round bottom flask and 1.5 ml concentrated HCl was added drop by drop with stirring the flask at 5–10 °C temperature. After complete addition of conc. HCl, 15 ml of ethylene glycol was added slowly, mixed and 0.75 g of benzo[d]thiazol-2-amine was added. Then flask was vigorously shaken and refluxed for 3 h. Mixture was cooled at room temperature and the mixture was poured drop by drop into crushed ice to obtain solid precipitate, which were filtered off and dried [26].

#### Synthesis of (E)-2-(3-(substitutedstyryl)-5-(substitutedphenyl)-4,5-dihydropyrazol-1-yl)benzo[d]thiazole derivatives (Z1-Z20) (VI)

2 mmol of (1*E*,4*E*)-1,5-bis (substitutedphenyl)penta-1,4-dien-3-one (III) was taken in a 50 ml round bottom flask and 15 ml of glacial acetic acid was added and shaken vigorously to dissolve completely. Then 2 mmol of 1-(benzo[d]thiazol-2-yl)hydrazine (V) was added in the solution and refluxed until the completion of reaction monitored by TLC. The reaction was cooled at room temperature and pour the solution into crushed ice, drop by drop, to obtain solid precipitate. The product was filtered and washed it with cold water and dried [4].

### Physicochemical and spectral characterization

*(E)-2-(3-(4-Chlorostyryl)-5-(4-chlorophenyl)-4,5-dihydropyrazol-1-yl)benzo[d]thiazole (Z1)*: Yield: 66.6%; m.p.: 129–132 ºC; R_f_: 0.7 (Benzene:Chloroform 5:5); FT-IR (KBr), v_max_ (cm^−1^): 2923.36, 2857 (C–H stretching aliphatic), 1650.30 (C = N stretching), 1536.28 (C = C stretching aliphatic), 1490.68 (C = C stretching aromatic), 1327.02 (C–N stretching), 753.74 (C–S–C stretching), 710 (C–Cl stretching); ^1^HNMR (400 MHz, CDCl_3_, δ ppm): 7.69–7.73 (d, 2H, C_4_″ of benzothiazole ring), 7.56–7.58 (d, 2H, C_7_″of benzothiazole ring), 7.40–7.43 (t, 3H, C_5_″ and C_6_″ of benzothiazole ring), 7.30–7.31 (d, 2H, C_3_ and C_5_ of phenyl ring A), 7.15–7.17 (d, 2H, C_3_ and C_5_ of phenyl ring B), 7.12–7.14 (d, 2H, C_2_ and C_6_ of phenyl ring B), 7.04–7.08 (d, 2H, C_2_ and C_6_ of phenyl ring A), 6.69–6.75 (dd, 2H, J 16 MHz, ethylene group), 5.79–5.84 (dd, H_x_, C_4_ʹ of pyrazole ring), 3.78–3.85 (dd, 1H_b_, C_3_ʹ of pyrazole ring), 3.14–3.19 (dd, 1H_a_, C_3_ʹ of pyrazole ring). ^13^C NMR (300 MHz, CDCl3, δ, ppm), 188.47 (N = CS-N), 142.08 (C = N), 140.21 (C–S), 138.72 (C–N, pyrazooline), 136.53 (C–N, benzothiazole), 135.11 (C–Cl), 134.0.6, 133.22 (CH = CH), 129.56, 129.30, 129.26, 129.23, 128.28, 127.96, 126.37, 125.72, 122.89, 121.11, 120.51, 119.50, 63.57.

(*E)-2-(3-(3-Chlorostyryl)-5-(3-chlorophenyl)-4,5-dihydropyrazol-1-yl)benzo[d]thiazole (Z2)*: Yield: 54.6%; m.p.: 90–93 ºC; R_f_: 0.7 (Benzene:Chloroform 5:5); FT-IR (KBr), v_max_ (cm^−1^): 3062.39 (C–H stretching aromatic), 2925.49, 2860.4 (C–H stretching aliphatic), 1655.23 (C = N stretching), 1476.82 (C = C stretching aliphatic), 1429.08 (C = C aromatic stretching), 1320.09 (C–N stretching), 793.59 (C–S–C stretching), 711.90 (C–Cl stretching).

*(E)-2-(3-(3-Nitrostyryl)-5-(3-nitrophenyl)-4,5-dihydropyrazol-1-yl)benzo[d]thiazole (Z3)*: Yield: 43.1%; m.p.: 102-105ºC; R_f_: 0.6 (Toluene:Methanol 7:3); FT-IR (KBr), v_max_ (cm^−1^): 3084.43(C–H stretching aromatic), 2924.2, 2926.85 (C–H stretching aliphatic), 1630.89 (C = N stretching), 1564.41 (assym. NO_2_ stretching), 1526.81(C = C stretching aliphatic), 1478.26(C = C aromatic stretching), 1351.48 (sym. NO_2_ stretching), 1203.43 (C–N stretching), 736.35 (C–S–C stretching); ^1^HNMR (400 MHz, DMSO,d_6_ δ, ppm): 8.64 (s, 1H, C_2_ of phenyl ring A), 8.57 (s, 1H, C_2_ of phenyl ring B), 8.52–8.53 (t, 1H, C_5_ of phenyl ring B), 8.41–8.44 (d,1H,C_4_ of phenyl ring A),8.26–8.30 (t, 1H, C_5_ of phenyl ring A), 8.20–8.21 (d, 1H, C_4_ of phenyl ring B), 7.95–7.99 (d, 1H, C_6_ of phenyl ring A and B), 7.73–7.84 (t, 2H, C_5_ and C_6_ of benzothiazole ring), 7.69–7.73 (d, 1H, C_4_″ of benzothiazole ring), 7.56–7.60 (d, 1H, C_7_″ of benzothiazole ring), 6.00–6.05 (dd, 2H, J 12 MHz, ethylene group), 5.69–5.76 (dd, H_x_, C_4_ʹ pyrazole ring), 3.40–3.45 (dd, 1H_b_, C_3_ʹ of pyrazole ring), 3.26–3.32 (dd, 1H_a_ C_3_ʹof pyrazole ring).

*(E)-2-(3-(4-Nitrostyryl)-5-(4-nitrophenyl)-4,5-dihydropyrazol-1-yl)benzo[d]thiazole (Z4)*: Yield: 27.8%; m.p.: 167–170 ºC; R_f_: 0.8 (Toluene:Methanol 7:3); FT-IR (KBr), v_max_ (cm^−1^): 2941.7, 2857 (C–H stretching aliphatic), 1630.35 (C = N stretching), 1517.20 (Assym. NO_2_ stretching), 1444.43 (C = C aliphatic stretching), 1411.63 (C = C aromatic stretching), 1347.79 (Sym. NO_2_ Stretching), 1191.63 (C–N Stretching), 746.49 (C–S–C stretching).

*(E)-2-(3-(2-Nitrostyryl)-5-(2-nitrophenyl)-4,5-dihydropyrazol-1-yl)benzo[d]thiazole (Z5)*: Yield: 36.8%; m.p.: 138-141ºC; R_f_:0.8 (Benzene:Chloroform 5:5); FT-IR (KBr), v_max_ (cm^−1^): 2928.1, 2857 (C–H stretching aliphatic), 1625.76 (C = N stretching), 1570.77 (Assym. NO_2_ stretching), 1521.91(C = C aliphatic stretching), 1443.81(C = C stretching aromatic), 1344.93 (Sym. NO_2_ stretching), 1201.05 (C–N stretching), 748.79 (C–S–C stretching).

*(E)-2-(3-(2-Chlorostyryl)-5-(2-chlorophenyl)-4,5-dihydropyrazol-1-yl)benzo[d]thiazole (Z6)*: Yield: 60%; m.p.: 111–114 ºC; R_f_:0.6 (Benzene:Chloroform 5:5); FT-IR (KBr), v_max_ (cm^−1^): 3062.79 (C–H stretching aromatic), 2924.28 (C–H stretching aliphatic), 1660.66 (C = N stretching), 1565.52(C = C aliphatic stretching), 1471.72 (C = C stretching aromatic), 1316.26(C–N stretching), 752.45(C–S–C stretching), 692.04 (C–Cl stretching).

*(E)-2-(3-(3-Hydroxystyryl)-5-(3-hydroxyphenyl)-4,5-dihydropyrazol-1-yl)benzo[d]thiazole (Z7)*: Yield: 75.6%; m.p.: 157–160 ºC; R_f_:0.9 (Toluene:Methanol 7:3); FT-IR (KBr), v_max_ (cm^−1^): 3436.49 (OH str.), 2965.4, 2826.5 (C–H stretching aliphatic), 1620.83 (C = N stretching), 1539.90 (C = C aliphatic stretching), 1451.79 (C = C aromatic stretching), 1276.55 (C–N stretching),760.55(C–S–C stretching); ^1^HNMR (400 MHz, DMSO,d_6_ δ, ppm): 9.66 (s, OH), 8.05 (s, 2H, C_2_ of phenyl ring A and B), 7.79–7.81 (d, 1H, C_4_ of phenyl ring A), 7.67–7.71 (d, 1H, C_4_ of phenyl ring B), 7.05–7.82 (m, 4H, of benzothiazole ring), 6.86–6.88 (d, 2H, C_6_ of phenyl ring A and B), 6.64–6.68 (t, 2H, C_5_ of phenyl ring A and B), 6.70–6.76 (dd, 2H, J 16 MHz, ethylene group), 5.69–5.73(dd, H_x_, C_4_ʹof pyrazole ring), 3.82–3.90 (dd, 1H_b_, C_3_ʹ of pyrazole ring), 3.13–3.18 (dd, 1H_a_, C_3_ʹ of pyrazole ring). ^13^C NMR (300 MHz, DMSO-d6, δ, ppm), 188.95 (N = CS–N), 162.49 (C–OH), 158.23 (C = N), 155.69 (C–S), 152.67 (C–N, pyrazooline), 143.51 (C–N, benzothiazole), 138.39, 137.57 (CH = CH), 136.49, 130.41, 126.02, 122.36, 121.71, 120.07, 118.19, 115.35, 115.10, 114.43, 63.30.

*(E)-2-(3-(4-Hydroxystyryl)-5-(4-hydroxyphenyl)-4,5-dihydropyrazol-1-yl)benzo[d]thiazole (Z8)*: Yield: 53.7%; m.p.: 197–200 ºC; R_f_:0.8 (Toluene:Methanol 7:3); FT-IR (KBr), v_max_ (cm^−1^): 3454.05 (OH str.), 2928.1,2874 (C–H stretching aliphatic), 1637.60 (C = N stretching), 1573.34 (C = C aliphatic stretching), 1450.39 (C = C aromatic stretching), 1264.79 (C–N stretching), 752.98 (C–S–C stretching).

*(E)-2-(3-(4-Bromostyryl)-5-(4-bromophenyl)-4,5-dihydropyrazol-1-yl)benzo[d]-thiazole(Z9)*: Yield: 58.9%; m.p.: 148–151 ºC; R_f_:0.8 (Benzene:Chloroform 5:5); FT-IR (KBr), v_max_ (cm^−1^): 2928.1,2850.2 (C–H stretching aliphatic), 1648.04 (C = N stretching), 1564.19 C = C aliphatic stretching), 1486.95 (C = C aromatic stretching), 1325.34 (C–N stretching), 754.57 (C–S–C stretching), 593.40 (C–Br stretching).

*(E)-2-(3-(4-Methylstyryl)-5-(4-methylphenyl)-4,5-dihydropyrazol-1-yl)benzo[d]thiazole (Z10)*: Yield: 35.8%; m.p.: 143–146 ºC; R_f_:0.62 (Benzene:Chloroform 5:5); FT-IR (KBr), v_max_ (cm^−1^): 2918, 2853.6 (C–H stretching aliphatic), 1621.40 (C = N stretching), 1540.90 (C = C aliphatic stretching), 1442.87 (C = C aromatic stretching), 1228.88 (C–N stretching), 749.58 (C–S–C stretching); ^1^HNMR (400 MHz, CDCl_3_, δ, ppm): 7.72–7.76 (d, 2H, C_2_, C_6_ of phenyl ring B), 7.64–7.646 (d, 2H, C_2_, C_6_ of phenyl ring A), 7.53–7.55 (d, 2H, C_3_, C_5_ of phenyl ring B), 7.38–7.40 (d, 2H, C_3_, C_5_ of phenyl ring A),7.05–7.28 (m, 4H, of benzothiazole ring), 6.68–6.75 (dd, 2H, J 16 MHz, ethylene group), 5.76–5.79 (dd, H_x_,C_4_ʹ of pyrazole ring), 3.76–3.83 (dd, 1H_b_, C_3_ʹ of pyrazole ring), 3.16–3.21 (dd, 1H_a_, C_3_ʹ of pyrazole ring), 1.99 (s, 3H, CH_3_).

*(E)-2-(3-(4-Methoxystyryl)-5-(4-methoxyphenyl)-4,5-dihydropyrazol-1-yl)benzo[d]-thiazole (Z11)*: Yield: 26%; m.p.: 96–99 ºC; R_f_:0.53 (Benzene:Chloroform 5:5); FT-IR (KBr), v_max_ (cm^−1^): 2961.60, 2840 (C–H stretching aliphatic), 1631.46 (C = N stretching), 1511.24 (C = C aliphatic stretching), 1442.26 (C = C aromatic stretching), 1251.28 (C–N stretching), 1030.50 (OCH_3_ stretching), 757.24 (C–S–C stretching); ^1^HNMR (400 MHz, CDCl_3_, δ, ppm): 7.86–7.88 (d, 4H, C_3_, C_5_ of phenyl ring A and B), 7.59–7.61 (d, 4H, C_2_, C_6_ of phenyl ring A and B),6.95–7.02 (m, 4H, of benzothiazole ring), 6.69–6.76 (dd, 2H, J 16 MHz, ethylene group),3.88 (s, 3H, OCH_3_), 3.79 (d, H_x_, C_4_ʹ of pyrazole ring), 2.39–2.41 (d, 1H_b_, C_3_ʹ of pyrazole ring), 2.13–2.17 (d, 1H_a_, C_3_ʹ of pyrazole ring).

*(E)-2-(5-Phenyl-3-styryl-4,5-dihydropyrazol-1-yl)benzo[d]thiazole (Z12)*: Yield: 55.2%; m.p.:97–100 ºC; R_f_:0.63 (Benzene:Chloroform 5:5); FT-IR (cm^−1^): 3056.38,3026.3 (C–H stretching aromatic), 2928.1,2857 (C–H stretching aliphatic), 1651.32 (C = N stretching), 1536.43 (C = C aliphatic stretching), 1447.94 (C = C aromatic stretching), 1195.50 (C–N stretching), 762.34 (C–S–C stretching); ^1^HNMR (400 MHz, CDCl_3_, δ, ppm): 7.75–7.79 (d, 1H, C_4_ of benzothiazole ring),7.64–7.66 (t, 2H, C_5_ and C_6_ of benzothiazole ring), 7.34–7.54, (m, 10H, phenyl ring A and B), 7.10–7.14 (d, 1H, C_7_ of benzothiazole ring), 6.73–6.78 (dd, 2H, J 16 MHz, ethylene group), 5.78–5.83 (dd, H_x_, C_4_ʹ of pyrazole ring), 3.80–3.87 (dd, 1H_b_, C_3_ʹ of pyrazole ring), 3.19–3.25 (dd, 1H_a_, C_3_ʹ of pyrazole ring).

*(E)-2-(3-(4-Fluorostyryl)-5-(4-fluorophenyl)-4,5-dihydropyrazol-1-yl)benzo[d]thiazole (Z13)*: Yield: 55.4%; m.p.:113–116 ºC; R_f_:0.6 (Benzene:Chloroform 5:5); FT-IR (KBr), v_max_ (cm^−1^): 2928.1, 2850.2 (C–H stretching aliphatic), 1653.14 (C = N stretching), 1537.67(C = C aliphatic stretching 1414.98 (C = C aromatic stretching), 1280.22 (C–F stretching), 1193.39 (C–N stretching), 754.90 (C–S–C stretching); ^1^HNMR (400 MHz, CDCl_3_, δ, ppm): 7.70–7.75 (d, 2H, C_3_,C_5_ of phenyl ring A), 7.56–7.58 (d, 2H, C_3_ and C_5_ of phenyl ring B), 7.46–7.50 (d, 1H, C_4_″ of benzothiazole ring), 7.33–7.37 (d, 1H, C_7_″ of benzothiazole ring),7.11–7.17 (t, 2H, C_5_″ and C_6_″ of benzothiazole ring), 7.06–7.07 (d, 2H, C_2_ and C_6_ of phenyl ring B), 6.99–7.04 (d, 2H, C_2_ and C_6_ of phenyl ring A), 6.69–6.76 (dd, 2H, J 16 MHz, ethylene group), 5.84–5.88 (dd, H_x_, C_4_ʹ of pyrazole ring),3.78–3.85 (dd, 1H_b_, C_3_ʹ of pyrazole ring), 3.16–3.21 (dd, 1H_a_, C_3_ʹof pyrazole ring).

*(E)-2-(3-(3-Methoxystyryl)-5-(3-methoxyphenyl)-4,5-dihydropyrazol-1-yl)benzo[d]- thiazole (Z14)*: Yield:81.8%; m.p.:100–103 ºC; R_f_:0.71 (Benzene:Chloroform 5:5); FT-IR (KBr), v_max_ (cm^−1^): 3056.8, 3000.18 (C–H stretching aromatic), 2936.24; 2835 (C–H stretching aliphatic), 1601.66 (C = N stretching), 1541.86 (C = C aliphatic stretching), 1435.70 (C = C aromatic stretching), 1261.77 (C–N stretching), 1046.74 (OCH_3_ stretching) 755.35 (C–S–C stretching).

*(E)-2-(3-(2-Methoxystyryl)-5-(2-methoxyphenyl)-4,5-dihydropyrazol-1-yl)benzo[d]-thiazole (Z15)*: Yield: 92.5%; m.p.:96–99 ºC; R_f_:0.55 (Benzene:Chloroform 5:5); FT-IR (KBr), v_max_ (cm^−1^): 3060.2 (C–H stretching aromatic), 2935.31 (C–H stretching aliphatic), 1614.99 (C = N stretching), 1538.13 (C = C aliphatic stretching), 1443.06 (C = C aromatic stretching), 1245.62 (C–N stretching), 1025.38 (OCH_3_ stretching), 751.56 (C–S–C stretching).

*(E)-2-(3-(3-Bromostyryl)-5-(3-bromophenyl)-4,5-dihydropyrazol-1-yl)benzo[d]thiazole (Z16)*: Yield: 73.9%; m.p.: 201–204 ºC; R_f_:0.67 (Benzene:Chloroform 5:5); FT-IR (KBr), v_max_ (cm^−1^): 3060.55,3016.2 (C–H stretching aromatic), 2972.2,2926.16 (C–H stretching aliphatic), 1617.02 (C = N stretching), 1561.29 (C = C aliphatic stretching), 1466.39 (C = C aromatic stretching), 1364 0.16 (C–N stretching), 752.39 (C–S–C stretching) 617.41 (C–Br stretching).

*(E)-2-(3-(2,3-Dichlorostyryl)-5-(2,3-dichlorophenyl)-4,5-dihydropyrazol-1-yl)benzo-[d]thiazole (Z17)*: Yield: 82%; m.p.: 100–103 ºC; R_f_:0.72 (Benzene:Chloroform 5:5); FT-IR (KBr), v_max_ (cm^−1^): 3070.4 (C–H stretching aromatic), 2931.5,2860.4 (C–H stretching aliphatic), 1618.00 (C = N stretching), 1450.93 (C = C stretching aliphatic), 1411.07 (C = C aromatic stretching), 1181.02 (C–N Stretching), 749.52(C–S–C stretching), 722.10 (C–Cl stretching).

*(E)-2-(3-(2,6-Dichlorostyryl)-5-(2,6-dichlorophenyl)-4,5-dihydropyrazol-1-yl)benzo- [d]thiazole (Z18):* Yield: 94.5%; m.p.: 205–208 ºC; R_f_:0.52 (Benzene:Chloroform 5:5); FT-IR (KBr), v_max_ (cm^−1^): 3063.6(C–H stretching aromatic), 2931.5,2857(C–H stretching aliphatic), 1617.96(C = N stretching), 1437.90 (C = C stretching aliphatic), 1427.31 (C = C aromatic stretching), 1177.26 (C–N Stretching), 718.76 (C–Cl stretching), 746.32 (C–S–C stretching); ^1^HNMR (400 MHz, CDCl_3_, δ, ppm): 7.82–7.87 (d, 2H, C_3_, C_5_ of phenyl ring A), 7.69–7.71(d, 2H, C_3_, C_5_ of phenyl ring B), 7.36–7.42 (t, 1H, C_4_ of phenyl ring A),7.34–7.36 (t, 1H, C_4_ of phenyl ring B), 7.14–7.27 (m, 4H, of benzothiazole ring), 6.85–6.90 (dd, 2H, J 16 MHz, ethylene group), 6.42–6.47 (dd, H_x_, C_4_ʹ of pyrazole ring), 3.77–3.85 (dd, 1H_b_, C_3_ʹ of pyrazole ring), 3.38–3.45 (dd, 1H_a_, C_3_ʹ of pyrazole ring).

*(E)-2-(3-(2,5-Dimethoxystyryl)-5-(2,5-dimethoxyphenyl)-4,5-dihydropyrazol-1-yl) benzo[d]thiazole (Z19)*: Yield: 95.9%; m.p.:137–140 ºC; R_f_:0.57 (Benzene:Chloroform 5:5); FT-IR (KBr), v_max_ (cm^−1^): 3002.6,3063.6 (C–H stretching aromatic), 2941, 2834.73(C–H stretching aliphatic), 1615.19(C = N stretching), 1446.47 (C = C stretching aliphatic), 1420.51 (C = C aromatic stretching), 1178.12 (C–N stretching), 1021.77 (OCH_3_ stretching), 747.32 (C–S–C stretching).

*(E)-2-(3-(3,4,5-Trimethoxystyryl)-5-(3,4,5-trimethoxyphenyl)-4,5-dihydropyrazol-1-yl) benzo[d]thiazole (Z20)*: Yield: 97.7%; m.p.: 95–98 ºC; R_f_:0.74 (Benzene:Chloroform 5:5); FT-IR (KBr), v_max_ (cm^−1^): 2995.9 (C–H stretching aromatic), 2939.94,2838.24 (C–H stretching aliphatic), 1619.76 (C = N stretching), 1538.45, 1455.37 (C = C stretching aliphatic), 1418.06 (C = C aromatic stretching), 1187.75 (C–N Stretching), 1040.88 (OCH_3_ stretching), 757.07 (C–S–C stretching); ^1^HNMR (400 MHz, CDCl_3_, δ, ppm):7.67–7.71 (t, 1H, C_6_″ of benzothiazole ring), 7.57–7.59 (d, 1H, C_4_″ of benzothiazole ring), 7.14–7.16 (t, 1H, C_5_″ of benzothiazole ring), 6.98–7.02 (d, 1H, C_7_″ of benzothiazole ring), 6.87 (s, 2H, C_2_ and C_6_ of phenyl ring B), 6.70–6.76 (dd, 2H, J 16 MHz ethylene group), 6.56 (s, 2H, C_2_ and C_6_ of phenyl ring A), 5.70–5.74 (dd, H_x_, C_4_ of pyrazole ring), 3.94 (s, OCH_3_ group), 3.89–3.90 (dd, 1H_b_, C_3_ of pyrazole ring), 3.83–3.84 (dd, 1H_a_, C_3_ of pyrazole ring).

### In vitro biological evaluation

#### Anti-oxidant activity

The different concentrations (500, 250, 125, 62.5 and 31.25 µg/ml) of synthesized compounds (Z1–Z20) in DMSO were prepared and 1 ml of sample was taken in a test tube, 1 ml of DPPH solution was added in each test tube and a purple color was observed. The test tubes were placed in dark chamber for 30 min, purple color changed into yellow and after 30 min absorbance was determined by UV spectroscopy at 517 nm wavelength. DMSO was used as blank to set zero [39].

#### Anti-inflammatory activity

The synthesized compounds (Z1–Z20) were used for the preparation of different concentrations (500, 250, 125, 62.5 and 31.25 ug/ml) in DMSO and 1 ml of each resulting solutions was taken in different test tubes. Then 1.4 ml of freshly prepared phosphate buffer (pH 6.4) and 0.1 ml egg albumin from fresh egg was transferred in each test tube containing different solutions for determining anti-inflammatory activity. The resulting mixtures in test tubes were incubated in a BOD for 15 min at 37 ± 2 ºC and then heated for 5 min at 70 ºC temperature. The mixture of test tubes was cooled at room temperature and absorbance was determined by UV spectroscopy at 660 nm wavelength [40].

#### Antimicrobial activity

1 ml of test sample was taken in a test tube having 1 ml of nutrient medium and serial dilutions of 50, 25, 12.5, 6.25 and 3.125 µg/ml were prepared. Then inoculation of test strains was done by micropipette and incubated at 37 ºC for 24 h for bacterial strains and 48 h for *C. albicans* and 120 h for *R. oryzae*. Results were calculated by visual turbidity observed in test tubes. MIC was calculated by using lowest concentration that inhibits microbial growth [41, 42].

### Molecular docking

AutoDock Vina, the advanced docking program was employed to estimate the binding charcaterstics of synthesized compounds into the active sites of target protein [38, 43]. The crystal structures of PDB: 6COX Cyclooxygenase-2 (prostaglandin synthase-2) complexed with a selective inhibitor, SC-558 IN I222 space group) [44], PDB: 2CAG (Catalase compound II) [13], PDB:1U4G, Elastase of *P. aeruginosa* with an inhibitor [45] and PDB:1EA1 (cytochrome P450 14 alpha-sterol demethylase (CYP51) from Mycobacterium tuberculosis in complex with fluconazole [46] were retrieved from the protein data bank (www.rcsb.org). AutoDock tools were utilized for the enlightenment of A chain of the proteins in pdbqt format. Water molecules which did not participate in interactions were removed and polar hydrogen atoms were introduced. The 2D structures of ligands were figured in MarvinSketch and saved in mol2 format, and then AutoDock tools were utilized to convert into pdbqt format. Energy minimization was accomplished using MMFF94 force field. The docking studies were executed according to requisite conditions of grid box by AutoDock tools. The search grid was identified as center_x = 21.72, center_y = 23.606, center_z = 47.846 (PDB:6COX); center_x = 58.613, center_y = 15.29, center_z = 16.972 (PDB:2CAG); center_x = 19.067, center_y = 26.357, center_z = − 4.427 (PDB:1U4G); center_x = − 16.172, center_y = − 5.396, center_z = 62.468 (PDB:1EA1), for target proteins with dimension size_x = 60, size_y = 60, size_z = 60, respectively. The exhaustiveness was set to be 8. The results were visualized using PyMol and Discovery studio visualizer [47].

### Pharmacokinetic parameters

ADMET analysis of synthesized compounds was performed by Molinspiration online tool kit, OSIRIS property explorer and Pre ADMET online server [48–50].

## Supplementary Information

**Additional file 1: Fig. S1.** IR spectra of compound Z1 [(*E*)-2-(3-(4-chlorostyryl)-5-(4-chlorophenyl)-4,5-dihydropyrazol-1-yl)benzo[d]thiazole]. **Fig. S2.** ^1^H NMR spectra of compound Z1 [(*E*)-2-(3-(4-chlorostyryl)-5-(4-chlorophenyl)-4,5-dihydropyrazol-1-yl)benzo[d]thiazole]. **Fig. S3.** ^13^C NMR spectra of compound Z1 [(*E*)-2-(3-(4-chlorostyryl)-5-(4-chlorophenyl)-4,5-dihydropyrazol-1-yl)benzo[d]thiazole]. **Fig. S4.** IR spectra of compound Z2 [(*E*)-2-(3-(3-chlorostyryl)-5-(3-chlorophenyl)-4,5-dihydropyrazol-1-yl)benzo[d]thiazole]. **Fig. S5.** IR spectra of compound Z3 [(*E*)-2-(3-(3-nitrostyryl)-5-(3-nitrophenyl)-4,5-dihydropyrazol-1-yl)benzo[d]thiazole]. **Fig. S6.** ^1^H NMR spectra of compound Z3 [(*E*)-2-(3-(3-nitrostyryl)-5-(3-nitrophenyl)-4,5-dihydropyrazol-1-yl)benzo[d]thiazole]. **Fig. S7** IR spectra of compound Z4 [(*E*)-2-(3-(4-nitrostyryl)-5-(4-nitrophenyl)-4,5-dihydropyrazol-1-yl)benzo[d]thiazole]. **Fig. S8.** IR spectra of compound Z5 [(*E*)-2-(3-(2-nitrostyryl)-5-(2-nitrophenyl)-4,5-dihydropyrazol-1-yl)benzo[d]thiazole]. **Fig. S9.** IR spectra of compound Z6 [(*E*)-2-(3-(2-chlorostyryl)-5-(2-chlorophenyl)-4,5-dihydropyrazol-1-yl)benzo[d]thiazole]. **Fig. S10.** IR spectra of compound Z7 [(*E*)-2-(3-(3-hydroxystyryl)-5-(3-hydroxyphenyl)-4,5-dihydropyrazol-1-yl)benzo[d]thiazole]. **Fig. S11.** ^1^H NMR spectra of compound Z7 [(*E*)-2-(3-(3-hydroxystyryl)-5-(3-hydroxy-phenyl)-4,5-dihydropyrazol-1-yl)benzo[d]thiazole]. **Fig. S12.** ^13^C NMR spectra of compound Z7 [(*E*)-2-(3-(3-hydroxystyryl)-5-(3-hydroxy-phenyl)-4,5-dihydropyrazol-1-yl)benzo[d]thiazole]. **Fig. S13.** IR spectra of compound Z8 [(*E*)-2-(3-(4-hydroxystyryl)-5-(4-hydroxyphenyl)- 4,5-dihydropyrazol-1-yl)benzo[d]thiazole]. **Fig. S14.** IR spectra of compound Z9 [(*E*)-2-(3-(4-bromostyryl)-5-(4-bromophenyl)-4,5-dihydropyrazol-1-yl)benzo[d]thiazole]. **Fig. S15.** IR spectra of compound Z10 [(*E*)-2-(3-(4-methylstyryl)-5-(4-methylphenyl)-4,5-dihydropyrazol-1-yl)benzo[d]thiazole]. **Fig. S16.** ^1^H NMR spectra of compound Z10 [(*E*)-2-(3-(4-methylstyryl)-5-(4-methyl-phenyl)-4,5-dihydropyrazol-1-yl)benzo[d]thiazole]. **Fig. S17.** IR spectra of compound Z11 [(*E*)-2-(3-(4-methoxystyryl)-5-(4-methoxyphenyl)-4,5-dihydropyrazol-1-yl)benzo[d]thiazole]. **Fig. S18.** ^1^H NMR spectra of compound Z11 [(*E*)-2-(3-(4-methoxystyryl)-5-(4-methoxy-phenyl)-4,5-dihydropyrazol-1-yl)benzo[d]thiazole]. **Fig. S19.** IR spectra of compound Z12 [(*E*)-2-(5-phenyl-3-styryl-4,5-dihydropyrazol-1-yl)-benzo[d]thiazole]. **Fig. S20.** ^1^H NMR spectra of compound Z12 [(*E*)-2-(5-phenyl-3-styryl-4,5-dihydro-pyrazol-1-yl)-benzo[d]thiazole]. **Fig. S21.** IR spectra of compound Z13 [(*E*)-2-(3-(4-fluorostyryl)-5-(4-fluorophenyl)-4,5-dihydropyrazol-1-yl)benzo[d]thiazole]. **Fig. S22.** ^1^H NMR spectra of compound Z13 [(*E*)-2-(3-(4-fluorostyryl)-5-(4-fluoro-phenyl)-4,5-dihydropyrazol-1-yl)benzo[d]thiazole]. **Fig. S23.** IR spectra of compound Z14 [(*E*)-2-(3-(3-methoxystyryl)-5-(3-methoxyphenyl)-4,5-dihydropyrazol-1-yl)benzo[d]thiazole]. **Fig. S24.** IR spectra of compound Z15 [(*E*)-2-(3-(2-methoxystyryl)-5-(2-methoxyphenyl)-4,5-dihydropyrazol-1-yl)benzo[d]-thiazole]. **Fig. S25.** IR spectra of compound Z16 [(*E*)-2-(3-(3-bromostyryl)-5-(3-bromophenyl)-4,5-dihydropyrazol-1-yl)benzo[d]thiazole]. **Fig. S26.** IR spectra of compound Z17 [(*E*)-2-(3-(2,3-dichlorostyryl)-5-(2,3-dichlorophenyl)-4,5-dihydropyrazol-1-yl)benzo[d]thiazole]. **Fig. S27.** IR spectra of compound Z18 [(*E*)-2-(3-(2,6-dichlorostyryl)-5-(2,6-dichlorophenyl)-4,5-dihydropyrazol-1-yl)benzo[d]thiazole]. **Fig. S28.** ^1^H NMR spectra of compound Z18 [(*E*)-2-(3-(2,6-dichlorostyryl)-5-(2,6-dichlorophenyl)-4,5-dihydropyrazol-1-yl)benzo[d]thiazole]. **Fig. S29.** IR spectra of compound Z19 [(*E*)-2-(3-(2,5-dimethoxystyryl)-5-(2,5-dimethoxyphenyl)-4,5-dihydropyrazol-1-yl) benzo[d]thiazole]. **Fig. S30.** IR spectra of compound Z20 [(*E*)-2-(3-(3,4,5-trimethoxystyryl)-5-(3,4,5-trimethoxyphenyl)-4,5-dihydropyrazol-1-yl) benzo[d]thiazole]. **Fig. S31.** ^1^H NMR spectra of compound Z20 [(*E*)-2-(3-(3,4,5-trimethoxystyryl)-5-(3,4,5-trimethoxyphenyl)-4,5-dihydropyrazol-1-yl) benzo[d]thiazole].

## Data Availability

All data generated or analysed during this study are included in this published article [and its supplementary information files].
