# Supplementary material for: Synthesis, in silico studies and biological screening of (E)-2-(3-(substitutedstyryl)-5-(substitutedphenyl)-4,5-dihydropyrazol-1-yl)benzo[d]thiazole derivatives as an anti-oxidant, anti-inflammatory and antimicrobial agents
Source: BMC Chem. 2022 Nov 24;16(1):103. doi: 10.1186/s13065-022-00901-2 (PMC9694607; doi:10.1186/s13065-022-00901-2)
Supplement: Supplementary file 1 — Additional file 1: Fig. S1. IR spectra of compound Z1 [(E)-2-(3-(4-chlorostyryl)-5-(4-chlorophenyl)-4,5-dihydropyrazol-1-yl)benzo[d]thiazole]. Fig. S2. 1H NMR spectra of compound Z1 [(E)-2-(3-(4-chlorostyryl)-5-(4-chlorophenyl)-4,5-dihydropyrazol-1-yl)benzo[d]thiazole]. Fig. S3. 13C NMR spectra of compound Z1 [(E)-2-(3-(4-chlorostyryl)-5-(4-chlorophenyl)-4,5-dihydropyrazol-1-yl)benzo[d]thiazole]. Fig. S4. IR spectra of compound Z2 [(E)-2-(3-(3-chlorostyryl)-5-(3-chlorophenyl)-4,5-dihydropyrazol-1-yl)benzo[d]thiazole]. Fig. S5. IR spectra of compound Z3 [(E)-2-(3-(3-nitrostyryl)-5-(3-nitrophenyl)-4,5-dihydropyrazol-1-yl)benzo[d]thiazole]. Fig. S6. 1H NMR spectra of compound Z3 [(E)-2-(3-(3-nitrostyryl)-5-(3-nitrophenyl)-4,5-dihydropyrazol-1-yl)benzo[d]thiazole]. Fig. S7 IR spectra of compound Z4 [(E)-2-(3-(4-nitrostyryl)-5-(4-nitrophenyl)-4,5-dihydropyrazol-1-yl)benzo[d]thiazole]. Fig. S8. IR spectra of compound Z5 [(E)-2-(3-(2-nitrostyryl)-5-(2-nitrophenyl)-4,5-dihydropyrazol-1-yl)benzo[d]thiazole]. Fig. S9. IR spectra of compound Z6 [(E)-2-(3-(2-chlorostyryl)-5-(2-chlorophenyl)-4,5-dihydropyrazol-1-yl)benzo[d]thiazole]. Fig. S10. IR spectra of compound Z7 [(E)-2-(3-(3-hydroxystyryl)-5-(3-hydroxyphenyl)-4,5-dihydropyrazol-1-yl)benzo[d]thiazole]. Fig. S11. 1H NMR spectra of compound Z7 [(E)-2-(3-(3-hydroxystyryl)-5-(3-hydroxy-phenyl)-4,5-dihydropyrazol-1-yl)benzo[d]thiazole]. Fig. S12. 13C NMR spectra of compound Z7 [(E)-2-(3-(3-hydroxystyryl)-5-(3-hydroxy-phenyl)-4,5-dihydropyrazol-1-yl)benzo[d]thiazole]. Fig. S13. IR spectra of compound Z8 [(E)-2-(3-(4-hydroxystyryl)-5-(4-hydroxyphenyl)- 4,5-dihydropyrazol-1-yl)benzo[d]thiazole]. Fig. S14. IR spectra of compound Z9 [(E)-2-(3-(4-bromostyryl)-5-(4-bromophenyl)-4,5-dihydropyrazol-1-yl)benzo[d]thiazole]. Fig. S15. IR spectra of compound Z10 [(E)-2-(3-(4-methylstyryl)-5-(4-methylphenyl)-4,5-dihydropyrazol-1-yl)benzo[d]thiazole]. Fig. S16. 1H NMR spectra of compound Z10 [(E)-2-(3-(4-methylstyryl)-5-(4-methyl-phenyl)-4,5- [file 13065_2022_901_MOESM1_ESM.docx]

**Spectral Characterization**


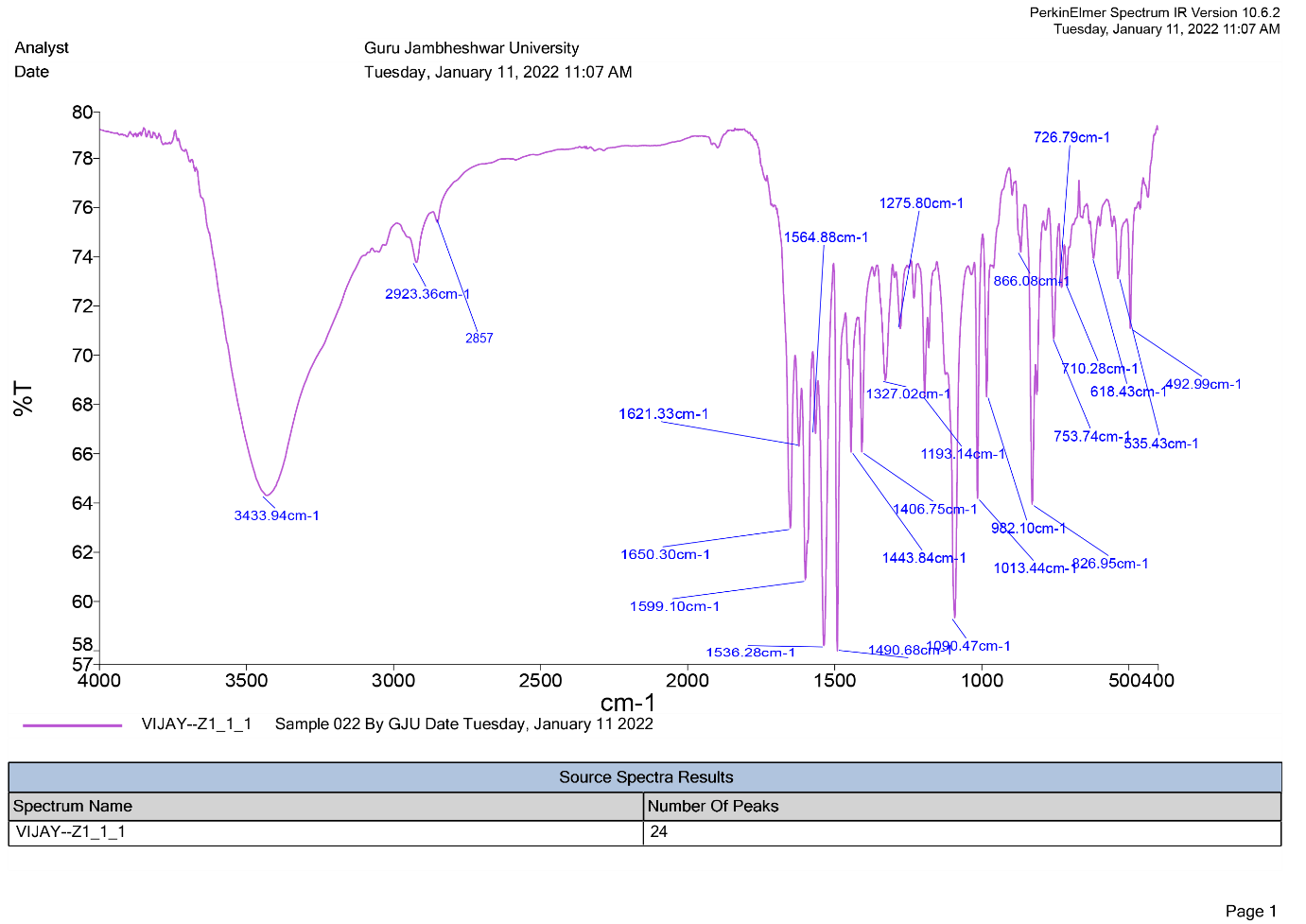

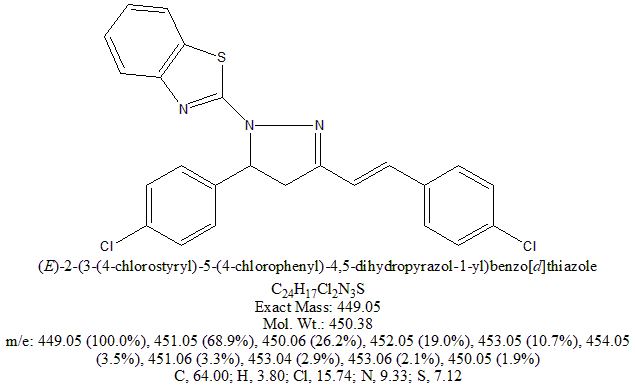


**Figure S1:** IR spectra of compound Z1 [(*E*)-2-(3-(4-chlorostyryl)-5-(4-chlorophenyl)-4,5-dihydropyrazol-1-yl)benzo[d]thiazole]


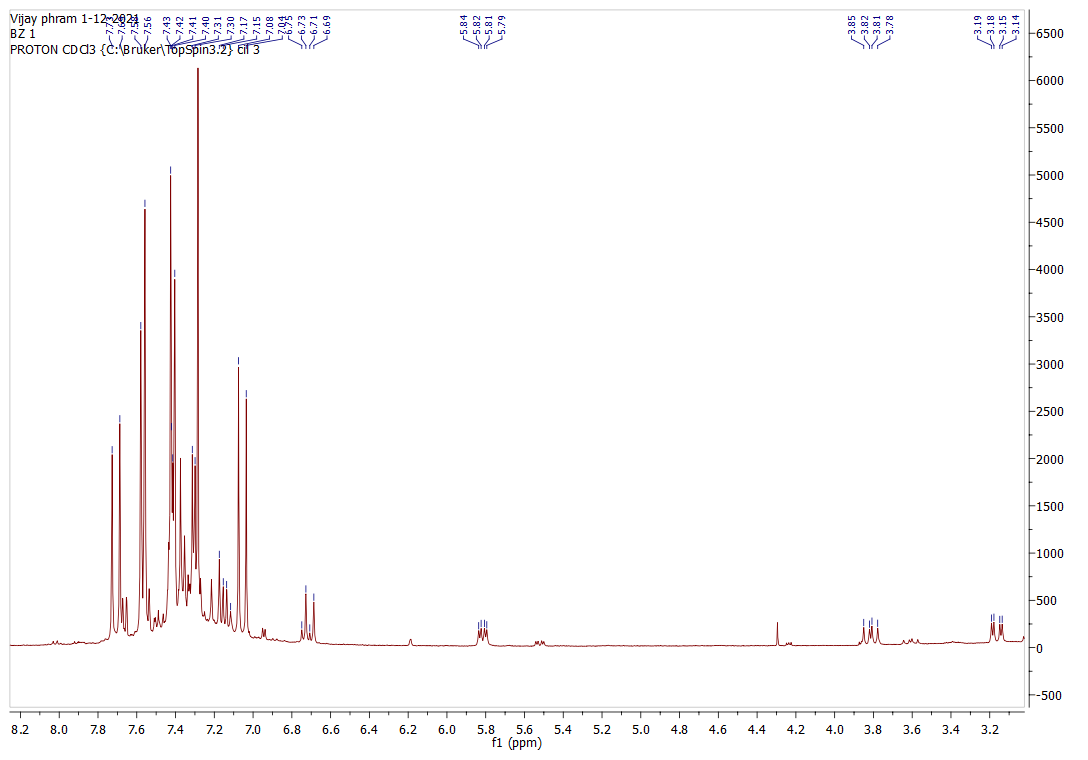


**Figure S2:** ^1^H NMR spectra of compound Z1 [(*E*)-2-(3-(4-chlorostyryl)-5-(4-chlorophenyl)-4,5-dihydropyrazol-1-yl)benzo[d]thiazole]


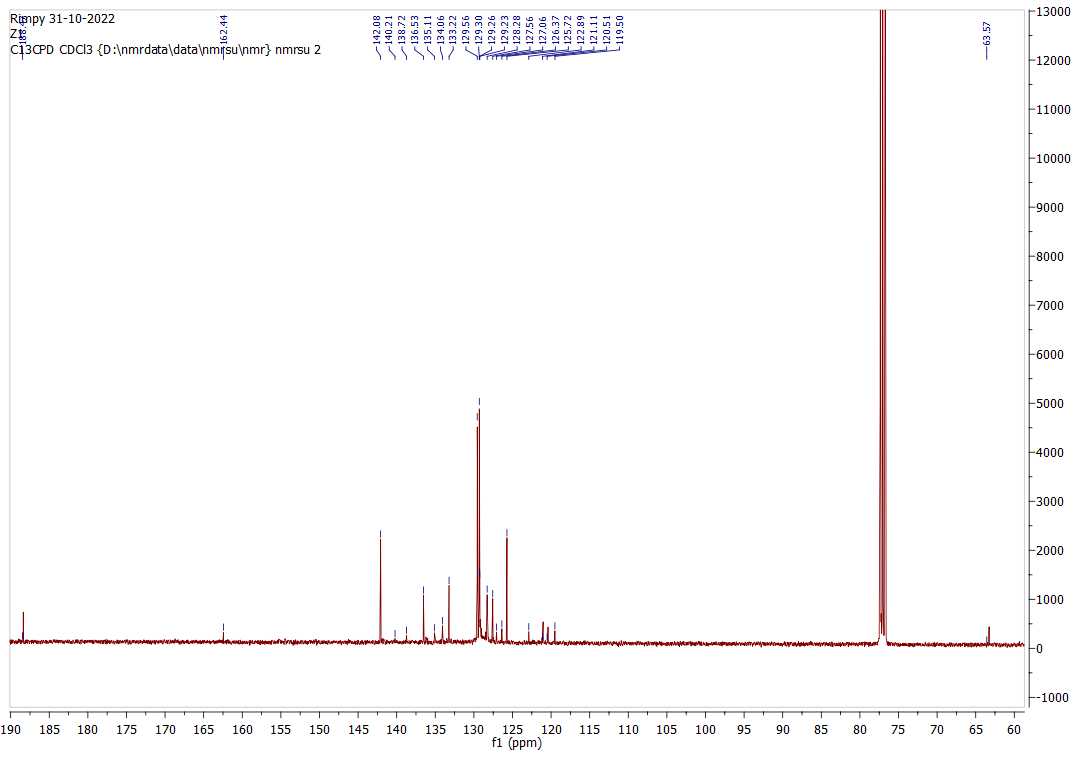


**Figure S3:** ^13^C NMR spectra of compound Z1 [(*E*)-2-(3-(4-chlorostyryl)-5-(4-chlorophenyl)-4,5-dihydropyrazol-1-yl)benzo[d]thiazole]


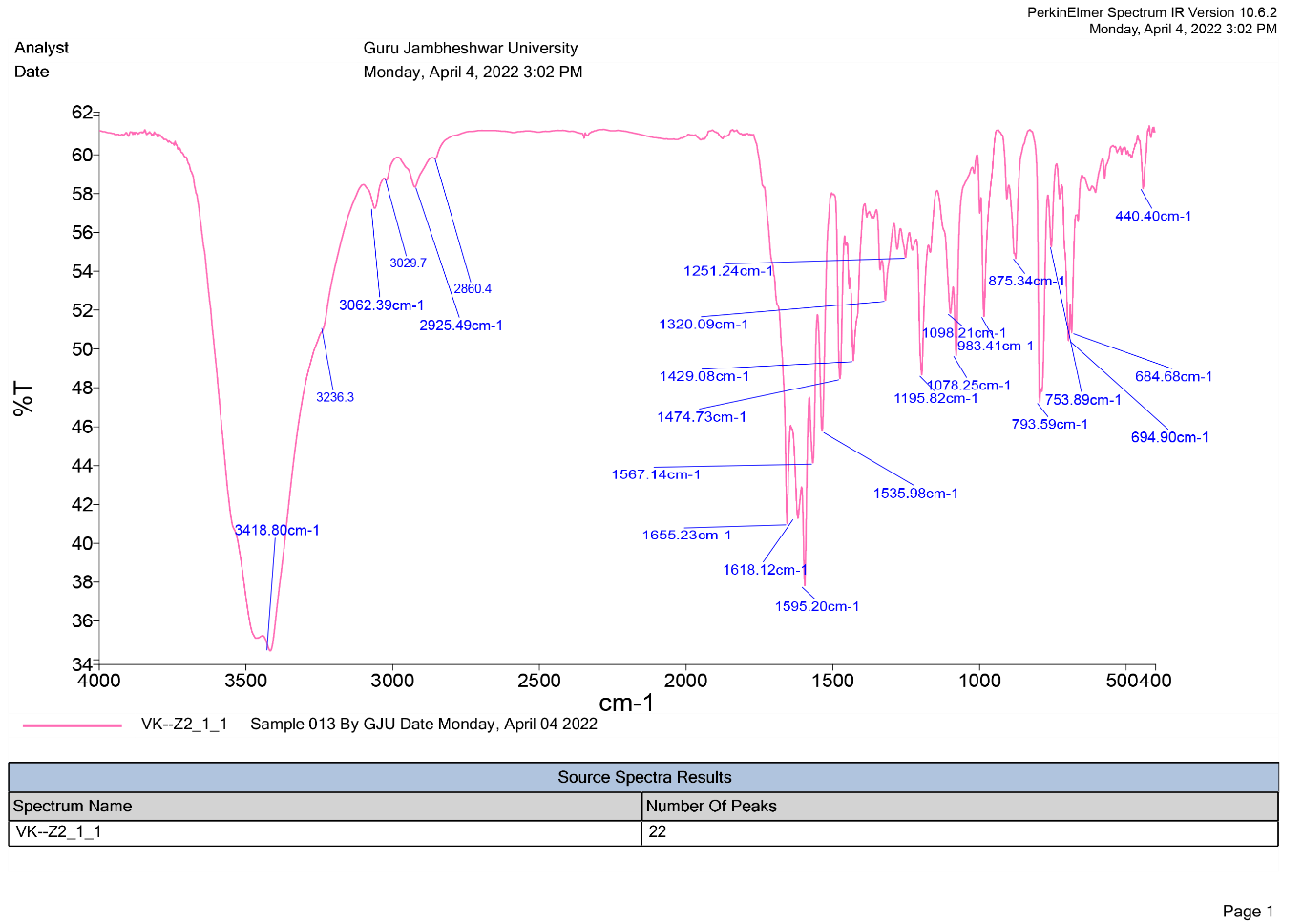

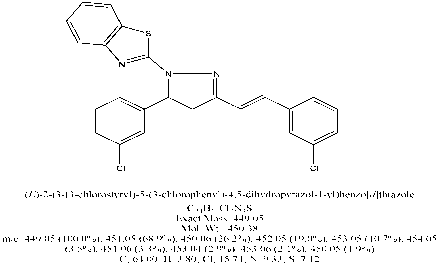


**Figure S4:** IR spectra of compound Z2 [(*E*)-2-(3-(3-chlorostyryl)-5-(3-chlorophenyl)-4,5-dihydropyrazol-1-yl)benzo[d]thiazole]


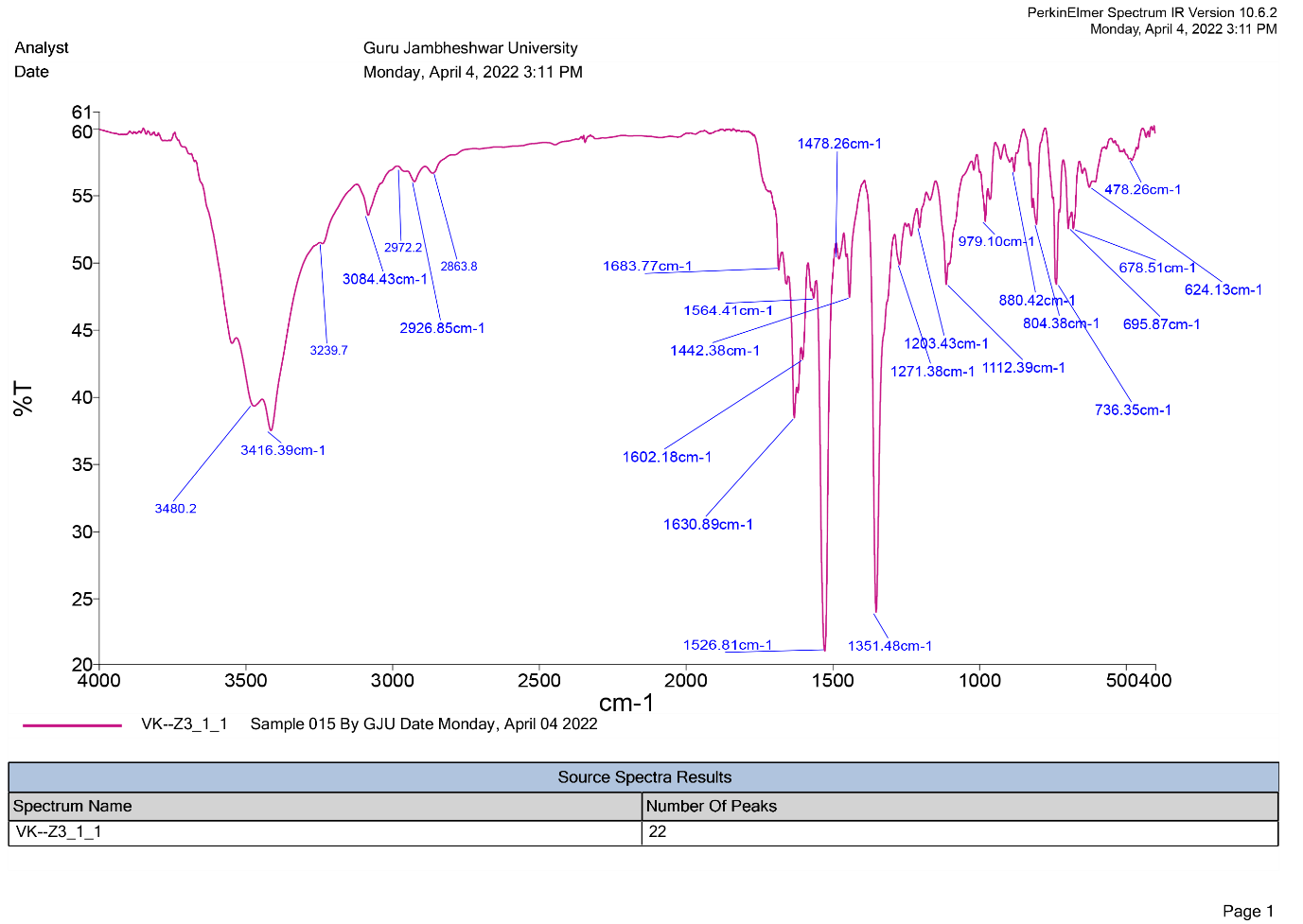

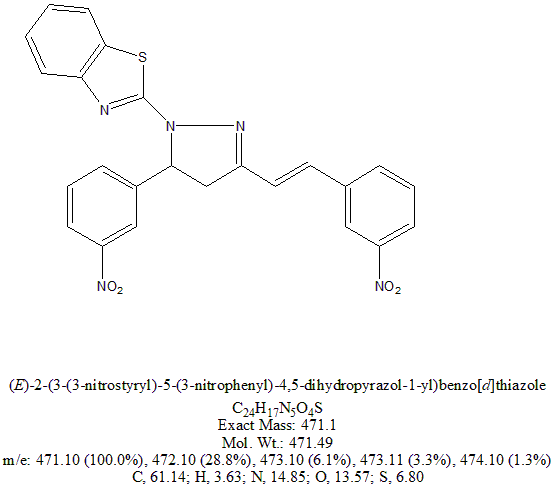


**Figure S5:** IR spectra of compound Z3 [(*E*)-2-(3-(3-nitrostyryl)-5-(3-nitrophenyl)-4,5-dihydropyrazol-1-yl)benzo[d]thiazole]

**
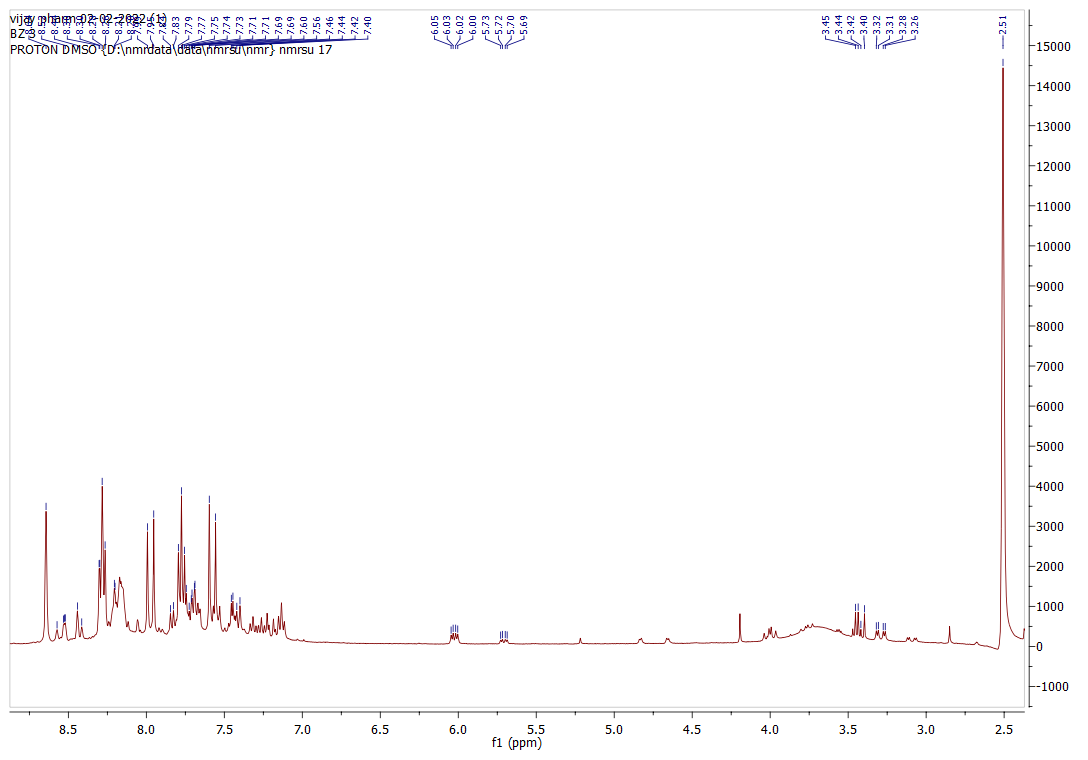
**

**Figure S6:** ^1^H NMR spectra of compound Z3 [(*E*)-2-(3-(3-nitrostyryl)-5-(3-nitrophenyl)-4,5-dihydropyrazol-1-yl)benzo[d]thiazole]


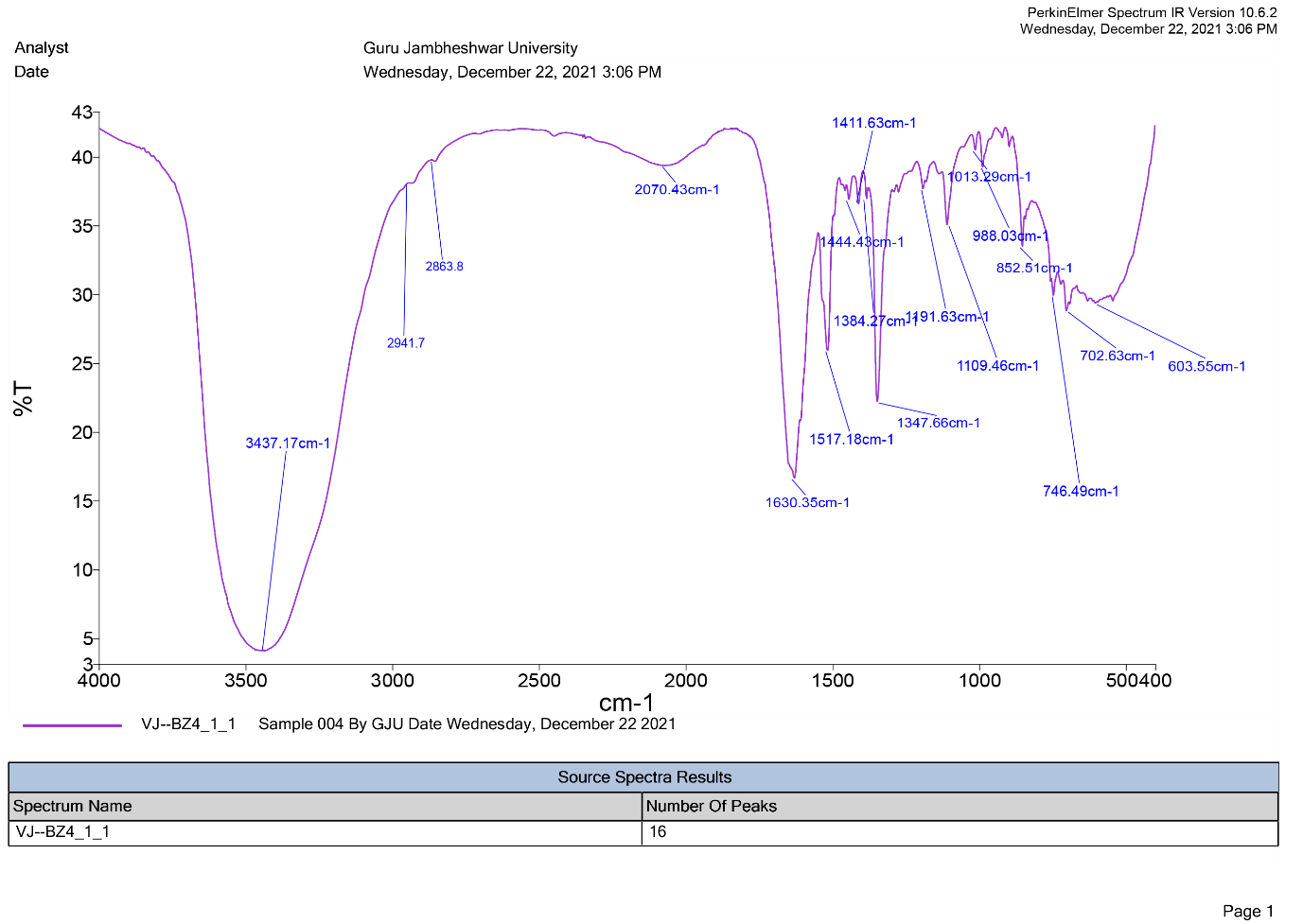

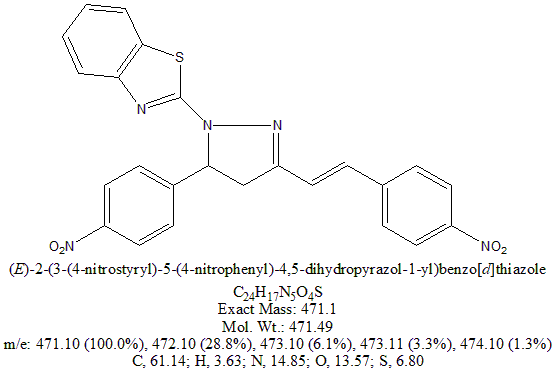


**Figure S7:** IR spectra of compound Z4 [(*E*)-2-(3-(4-nitrostyryl)-5-(4-nitrophenyl)-4,5-dihydropyrazol-1-yl)benzo[d]thiazole]


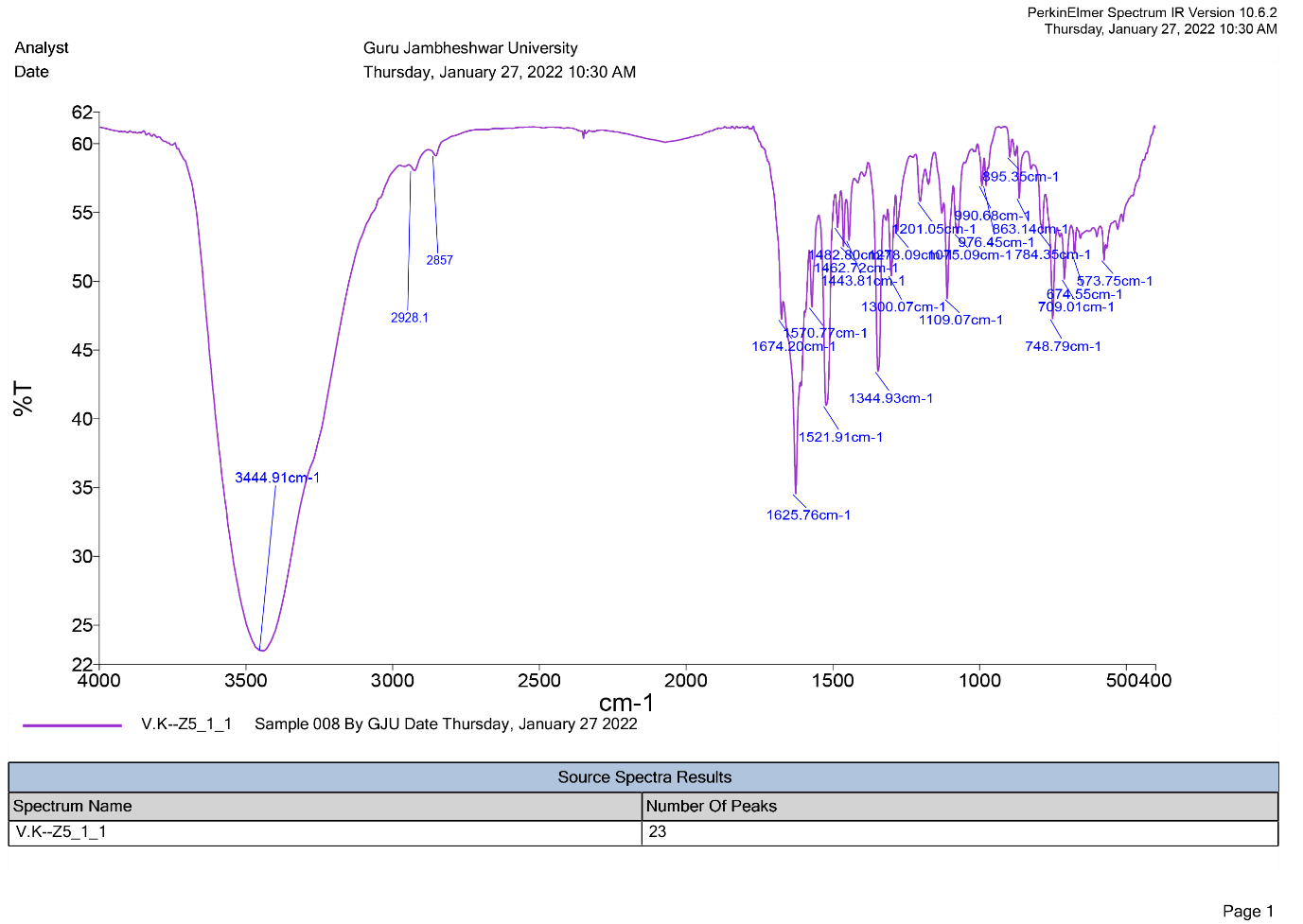

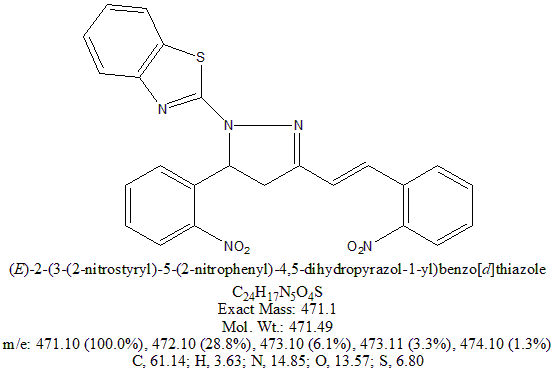


**Figure S8:** IR spectra of compound Z5 [(*E*)-2-(3-(2-nitrostyryl)-5-(2-nitrophenyl)-4,5-dihydropyrazol-1-yl)benzo[d]thiazole]


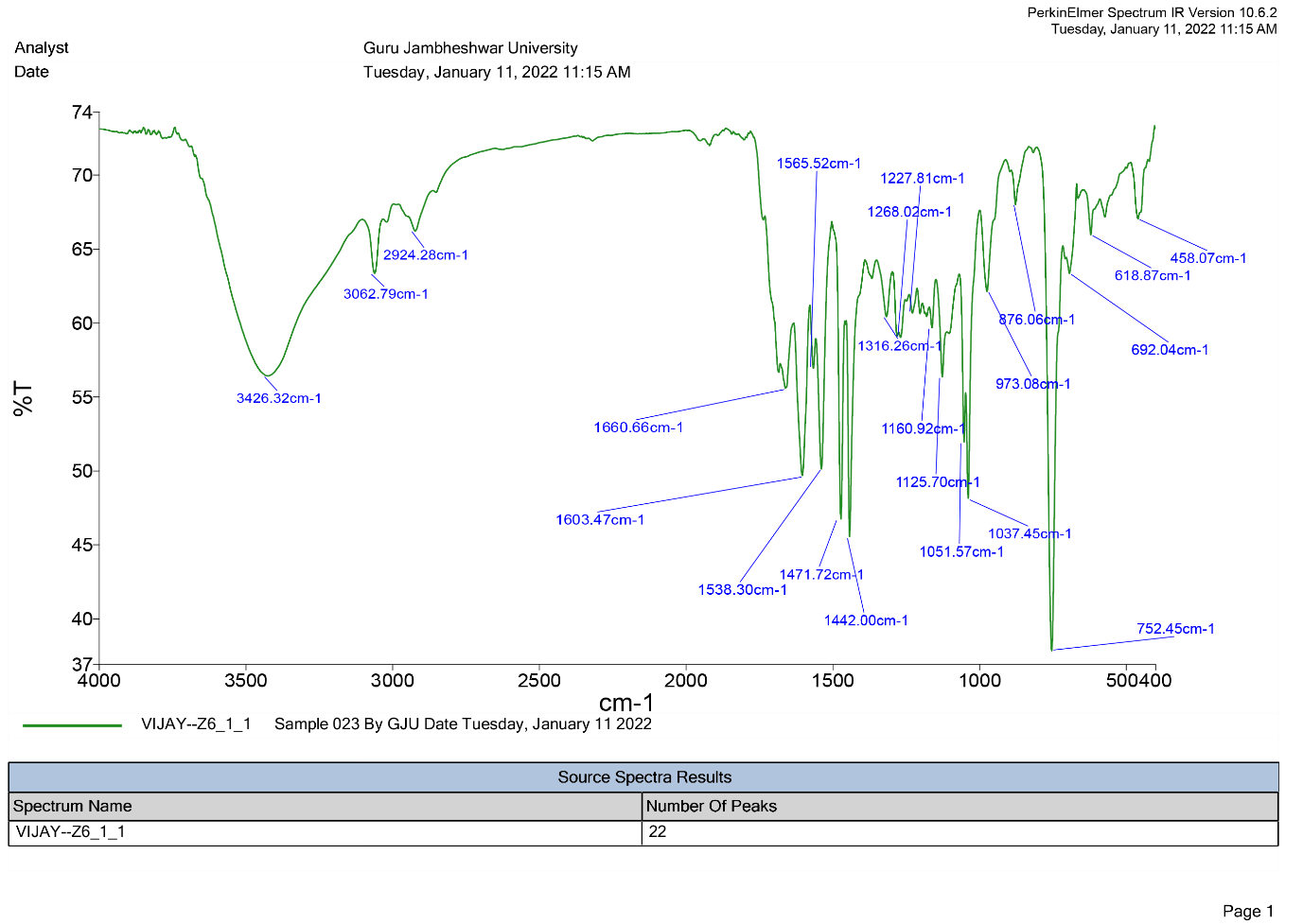

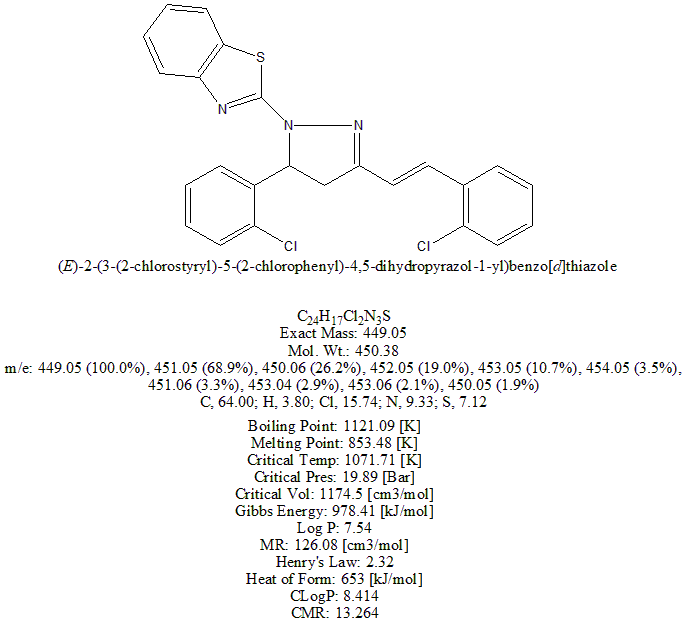


**Figure S9:** IR spectra of compound Z6 [(*E*)-2-(3-(2-chlorostyryl)-5-(2-chlorophenyl)-4,5-dihydropyrazol-1-yl)benzo[d]thiazole]


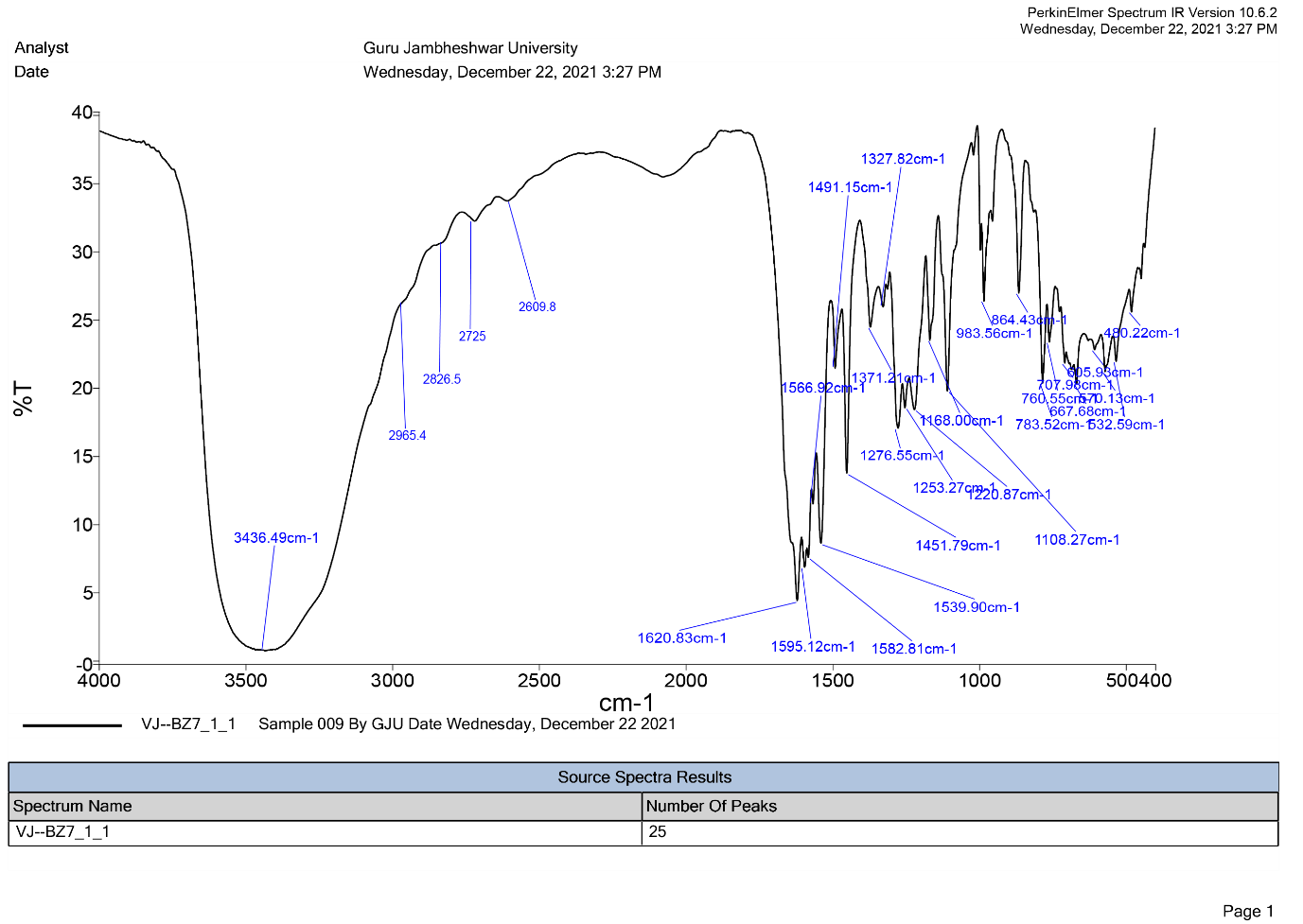

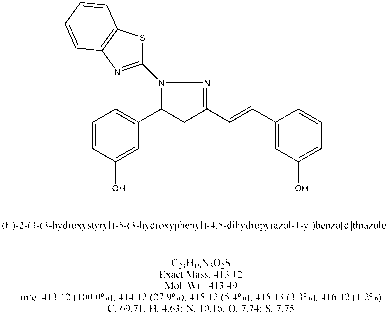


**Figure S10:** IR spectra of compound Z7 [(*E*)-2-(3-(3-hydroxystyryl)-5-(3-hydroxyphenyl)-4,5-dihydropyrazol-1-yl)benzo[d]thiazole]


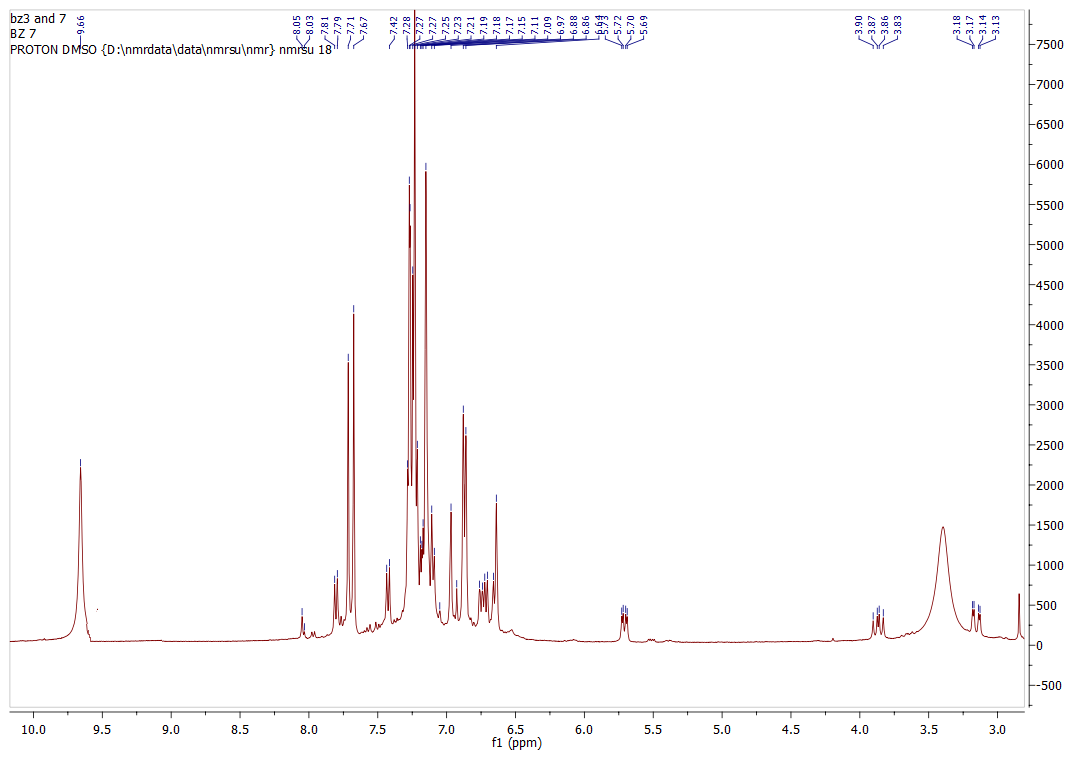


**Figure S11:** ^1^H NMR spectra of compound Z7 [(*E*)-2-(3-(3-hydroxystyryl)-5-(3-hydroxy-phenyl)-4,5-dihydropyrazol-1-yl)benzo[d]thiazole]


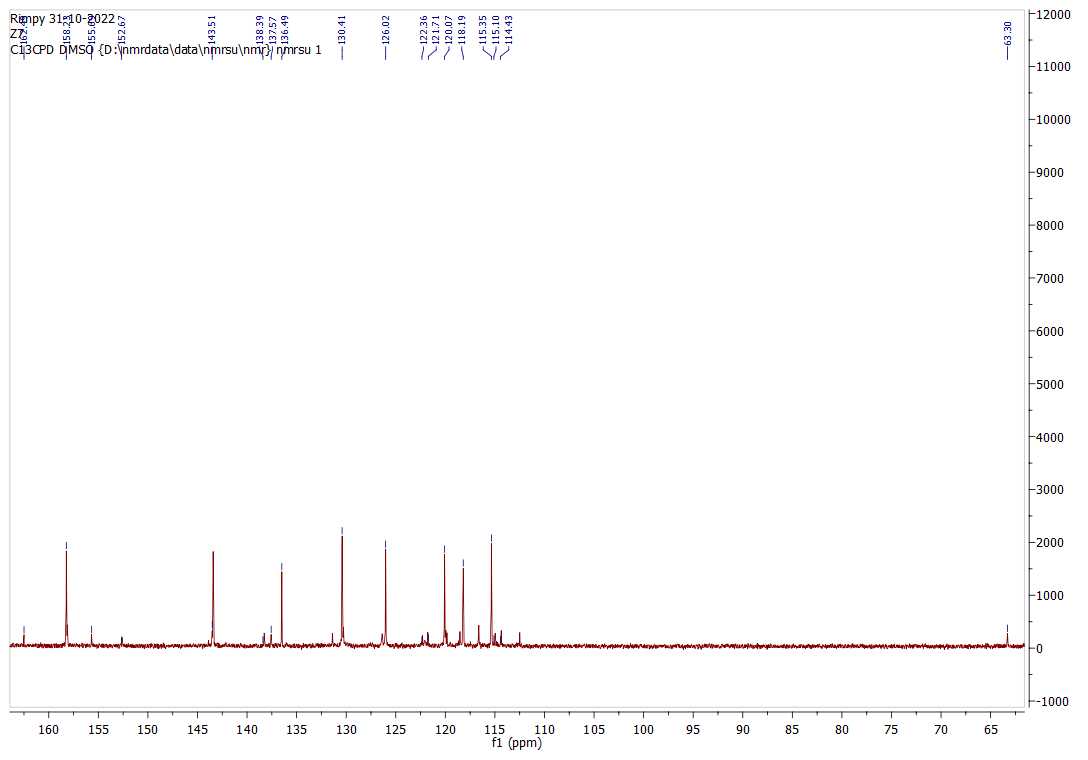


**Figure S12:** ^13^C NMR spectra of compound Z7 [(*E*)-2-(3-(3-hydroxystyryl)-5-(3-hydroxy-phenyl)-4,5-dihydropyrazol-1-yl)benzo[d]thiazole]


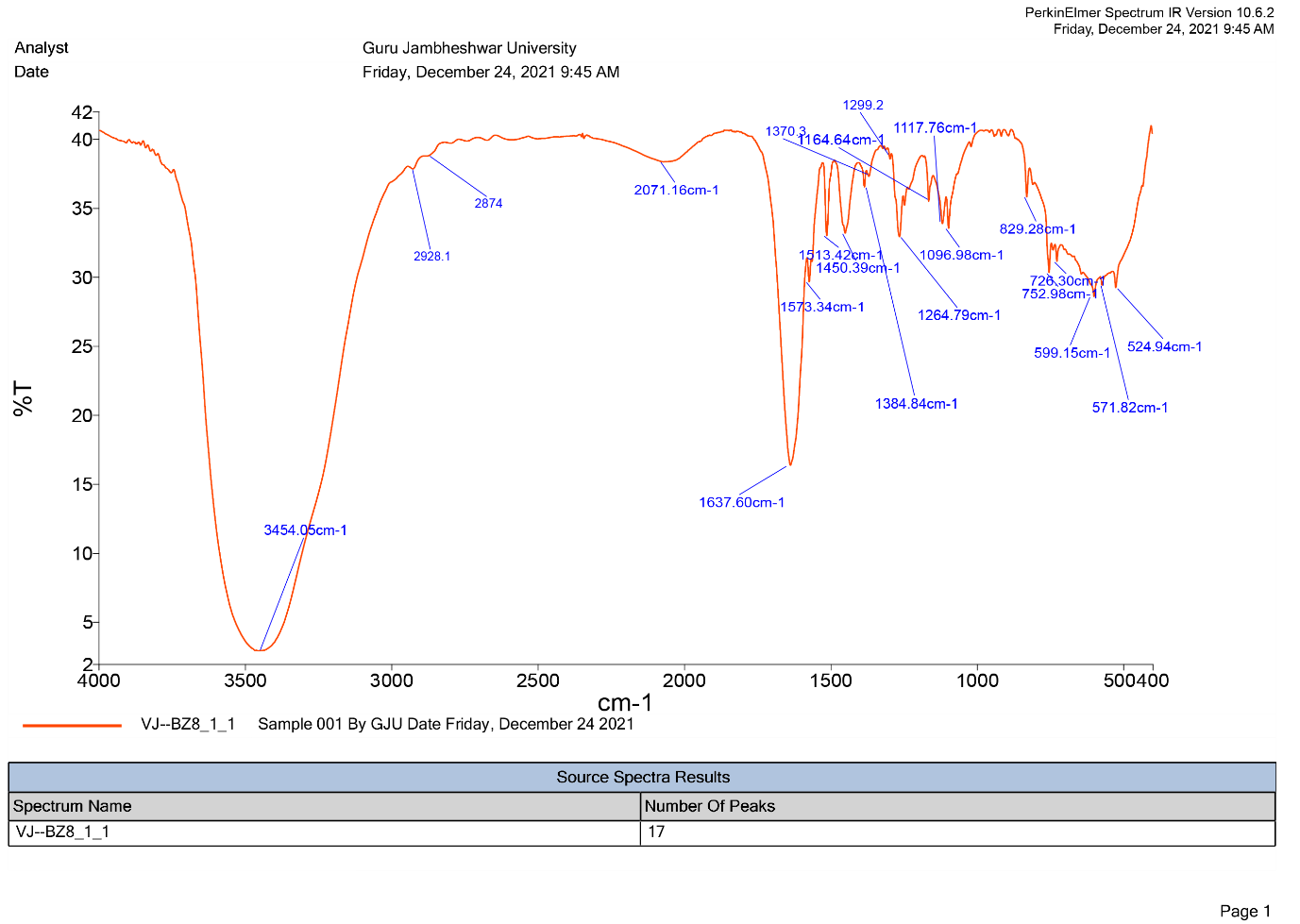

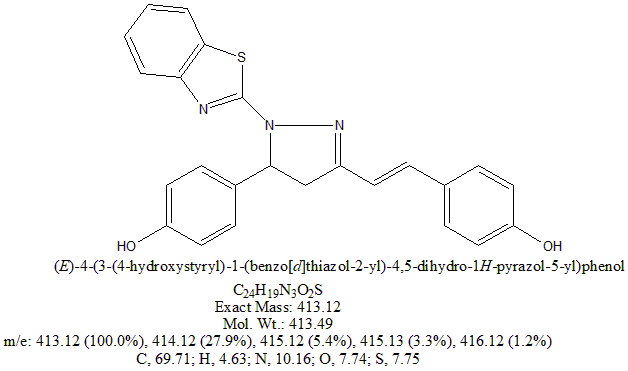


**Figure S13:** IR spectra of compound Z8 [(*E*)-2-(3-(4-hydroxystyryl)-5-(4-hydroxyphenyl)-

4,5-dihydropyrazol-1-yl)benzo[d]thiazole]


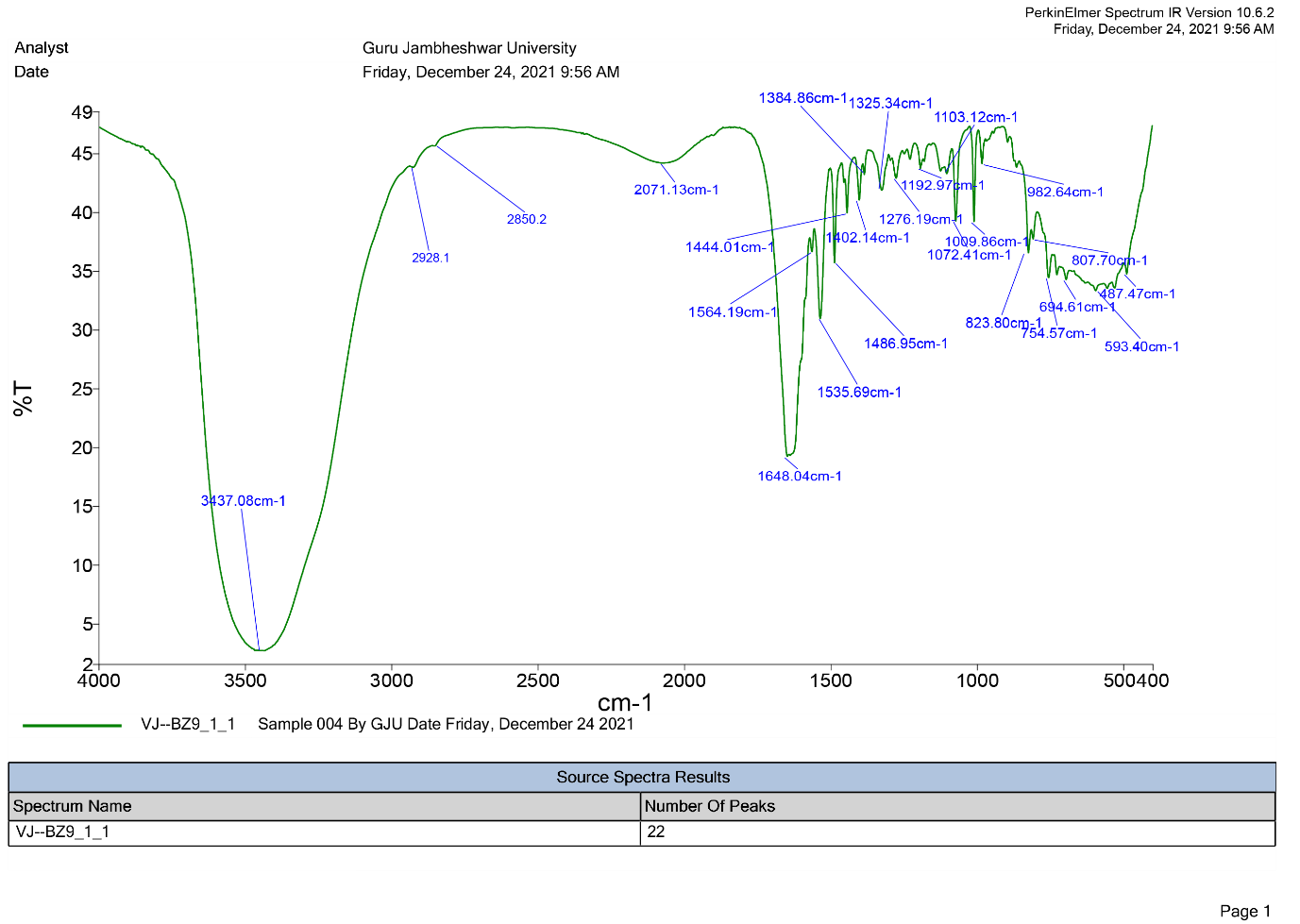

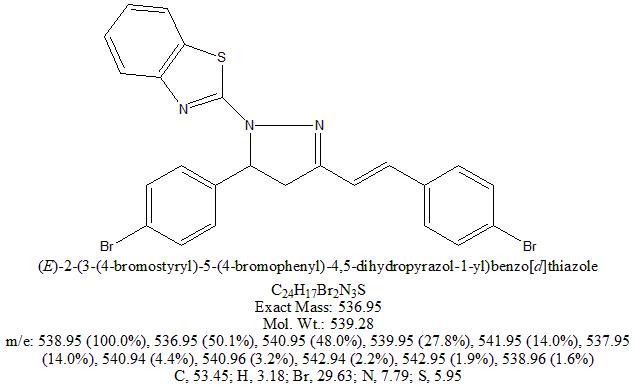


**Figure S14:** IR spectra of compound Z9 [(*E*)-2-(3-(4-bromostyryl)-5-(4-bromophenyl)-4,5-dihydropyrazol-1-yl)benzo[d]thiazole]


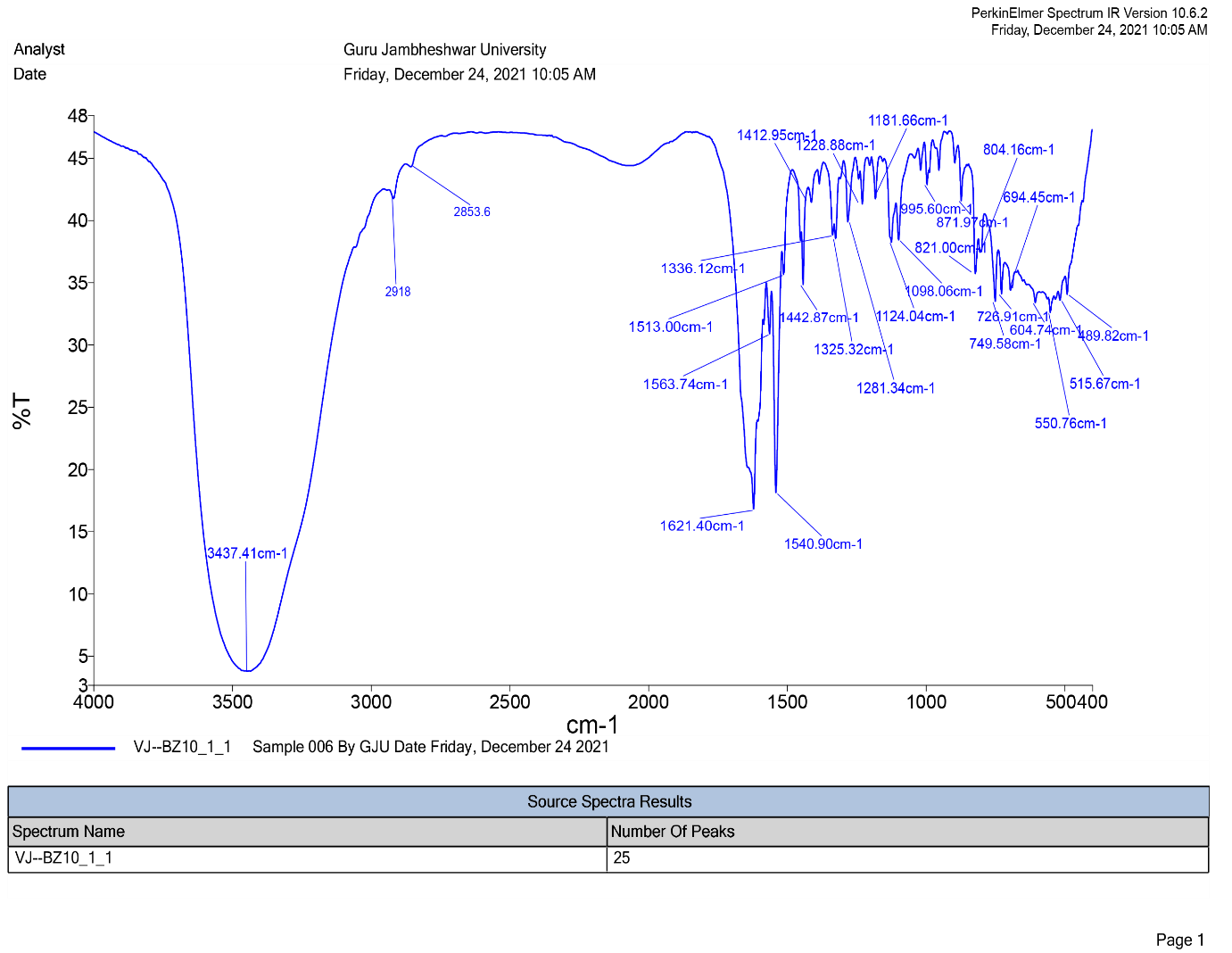

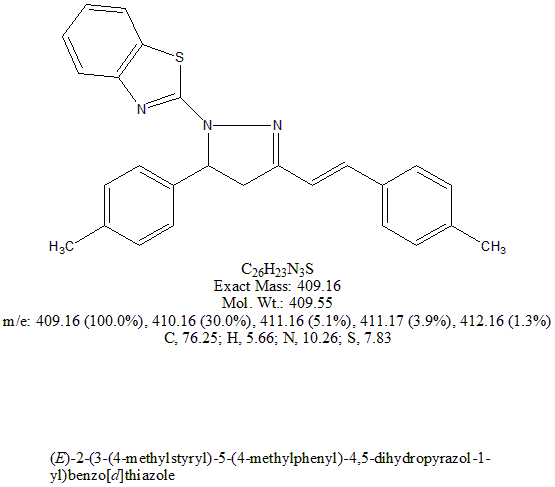


**Figure S15:** IR spectra of compound Z10 [(*E*)-2-(3-(4-methylstyryl)-5-(4-methylphenyl)-4,5-dihydropyrazol-1-yl)benzo[d]thiazole]

**
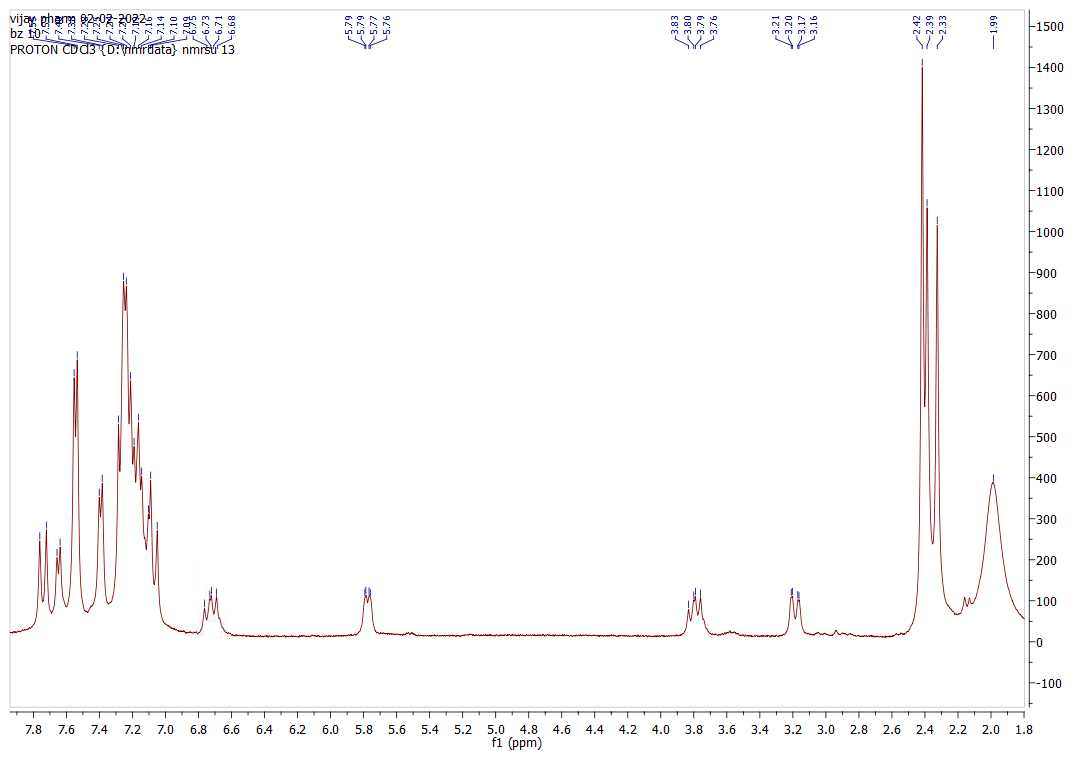
**

**Figure S16:** ^1^H NMR spectra of compound Z10 [(*E*)-2-(3-(4-methylstyryl)-5-(4-methyl-phenyl)-4,5-dihydropyrazol-1-yl)benzo[d]thiazole]


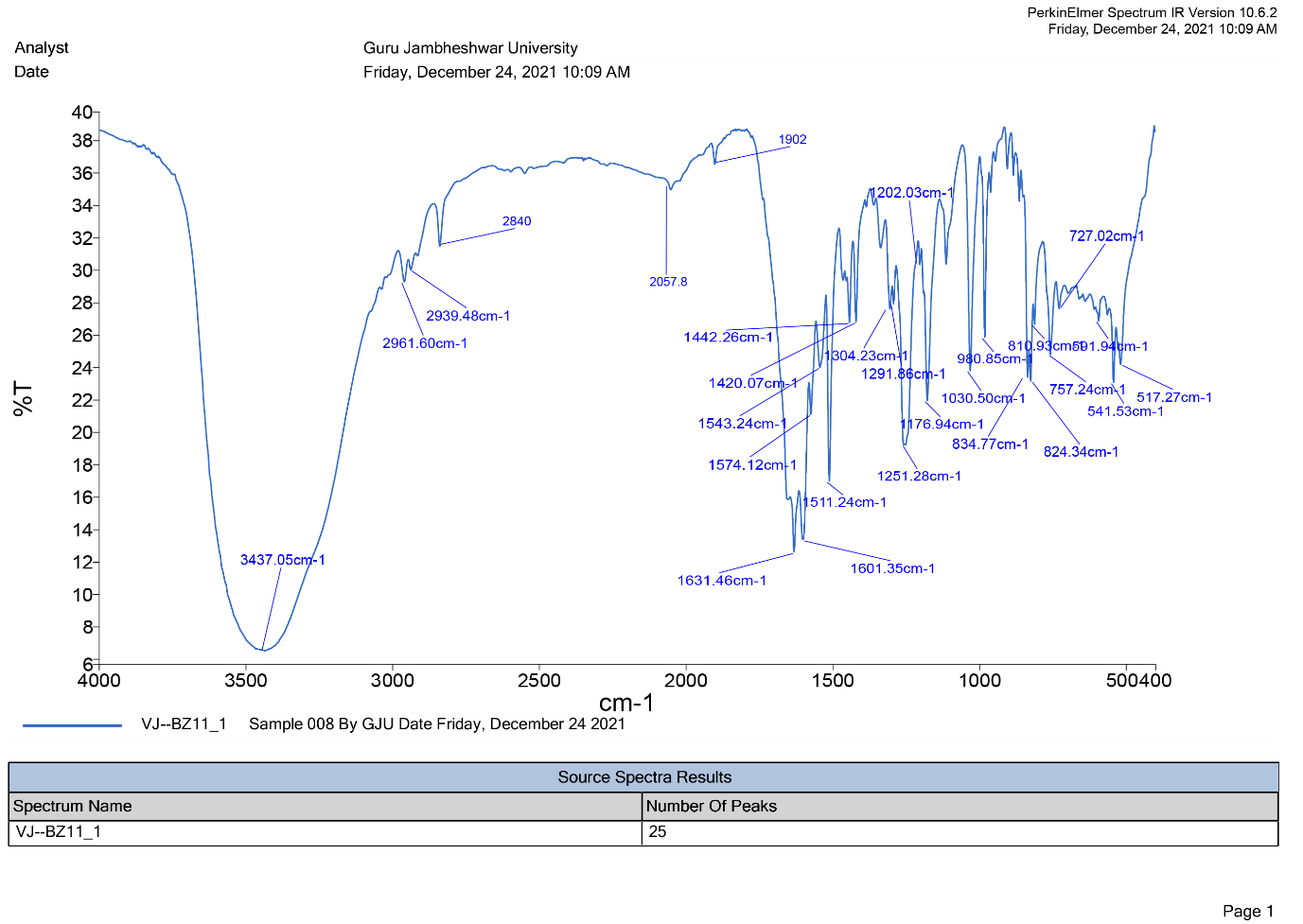

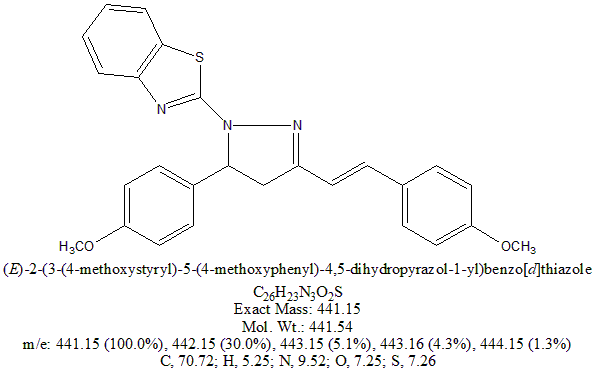


**Figure S17:** IR spectra of compound Z11 [(*E*)-2-(3-(4-methoxystyryl)-5-(4-methoxyphenyl)-4,5-dihydropyrazol-1-yl)benzo[d]thiazole]

**
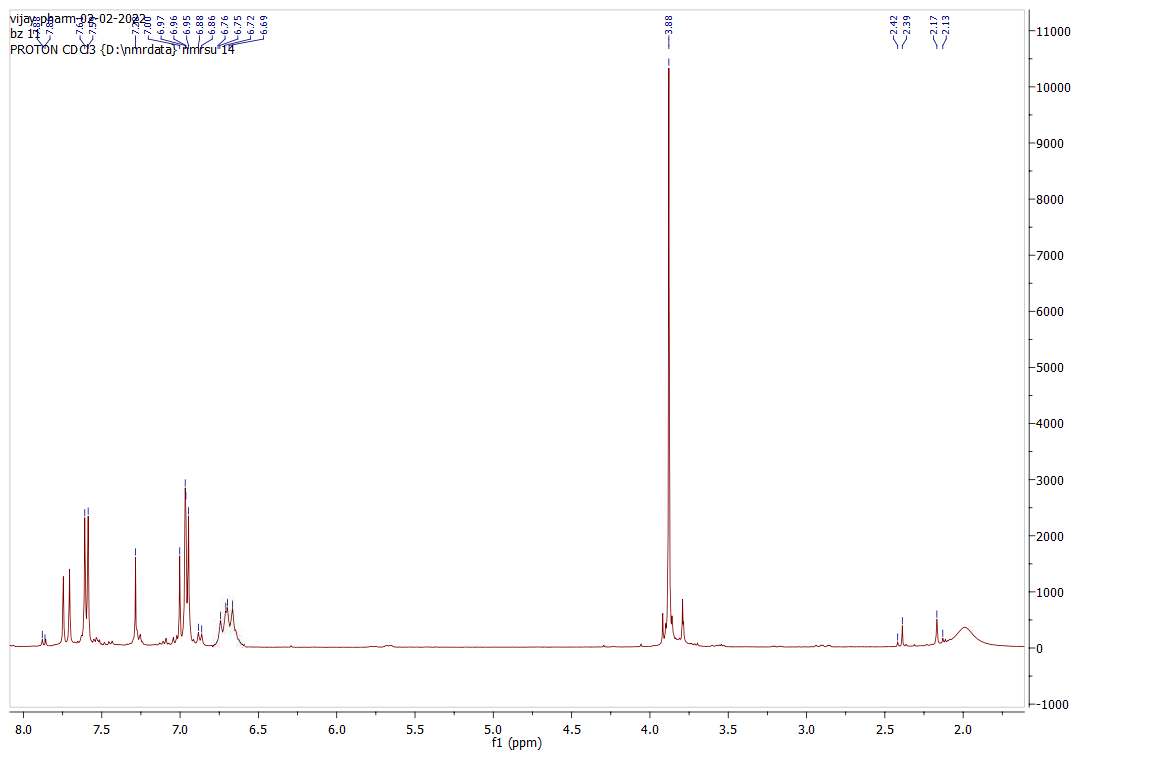
**

**Figure S18:** ^1^H NMR spectra of compound Z11 [(*E*)-2-(3-(4-methoxystyryl)-5-(4-methoxy-phenyl)-4,5-dihydropyrazol-1-yl)benzo[d]thiazole]


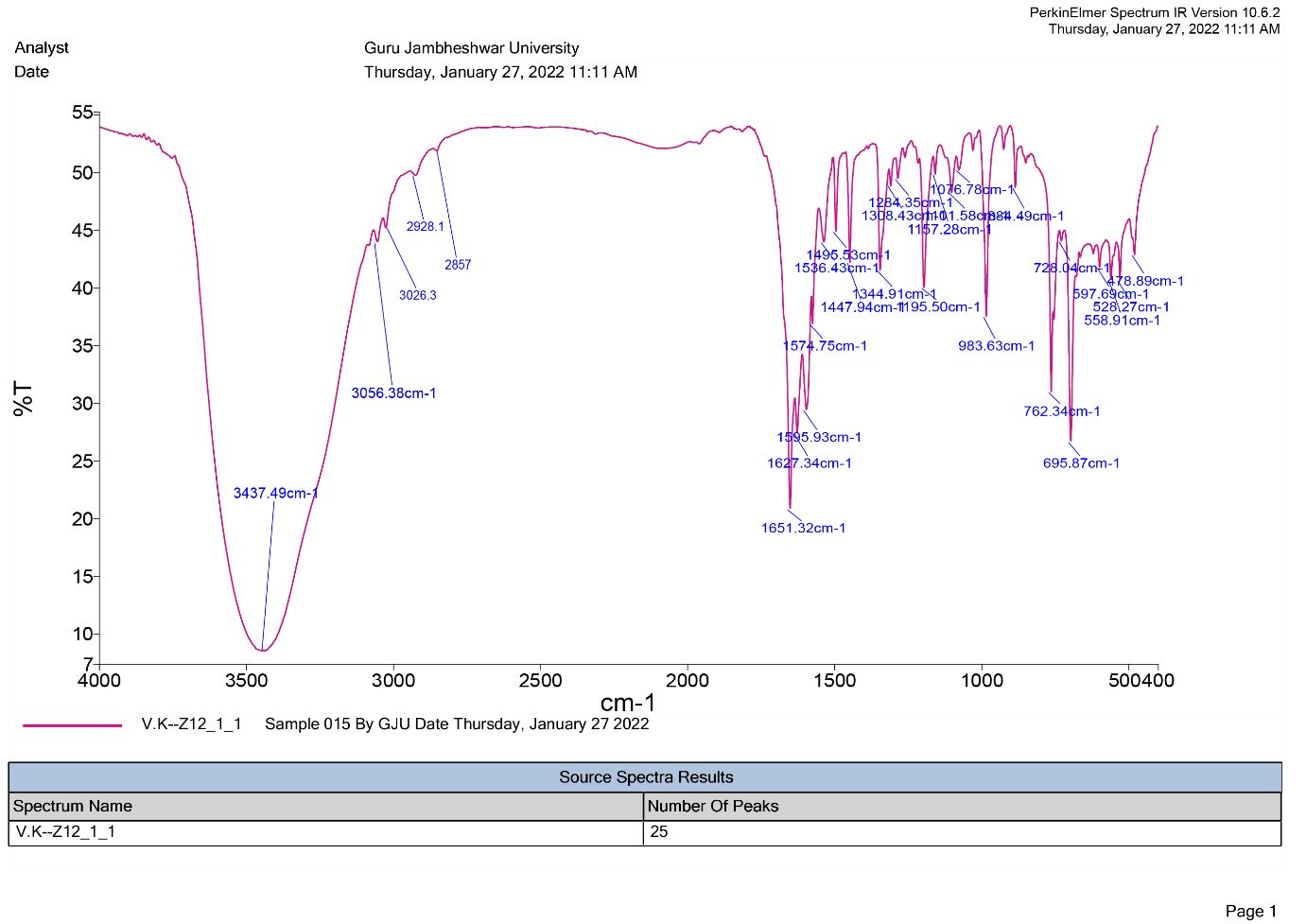

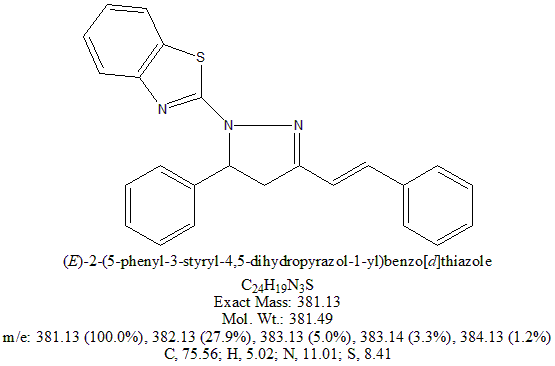


**Figure S19:** IR spectra of compound Z12 [(*E*)-2-(5-phenyl-3-styryl-4,5-dihydropyrazol-1-yl)-benzo[d]thiazole]


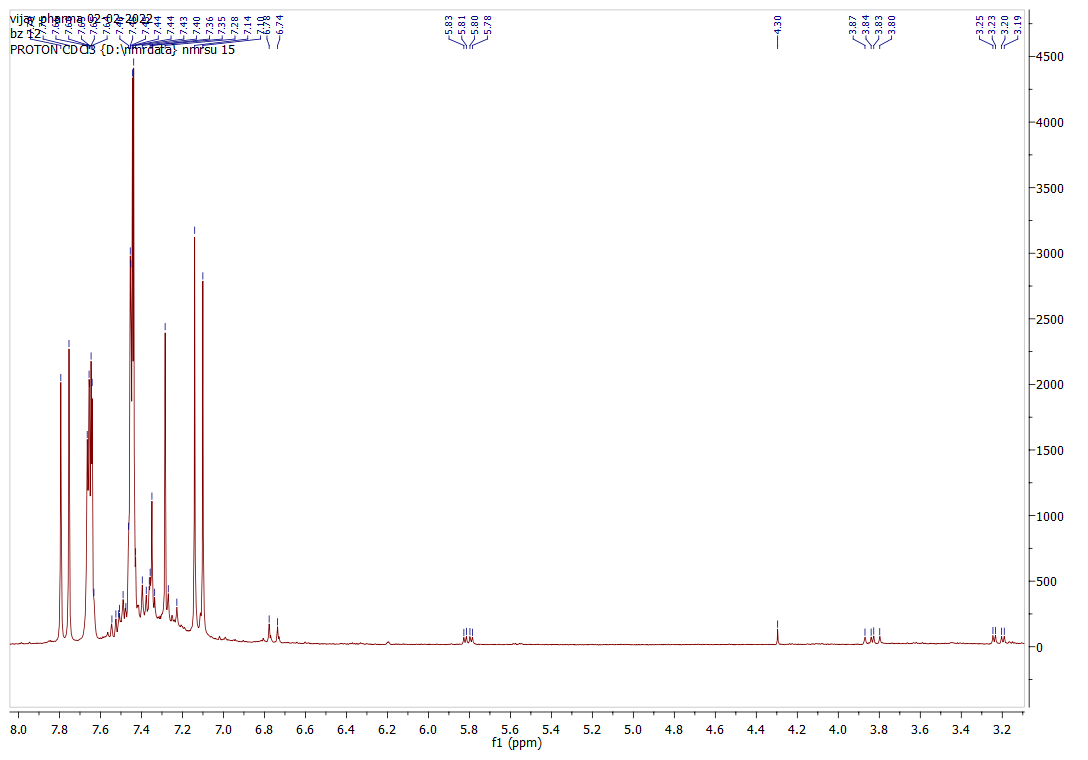


**Figure S20:** ^1^H NMR spectra of compound Z12 [(*E*)-2-(5-phenyl-3-styryl-4,5-dihydro-pyrazol-1-yl)-benzo[d]thiazole]


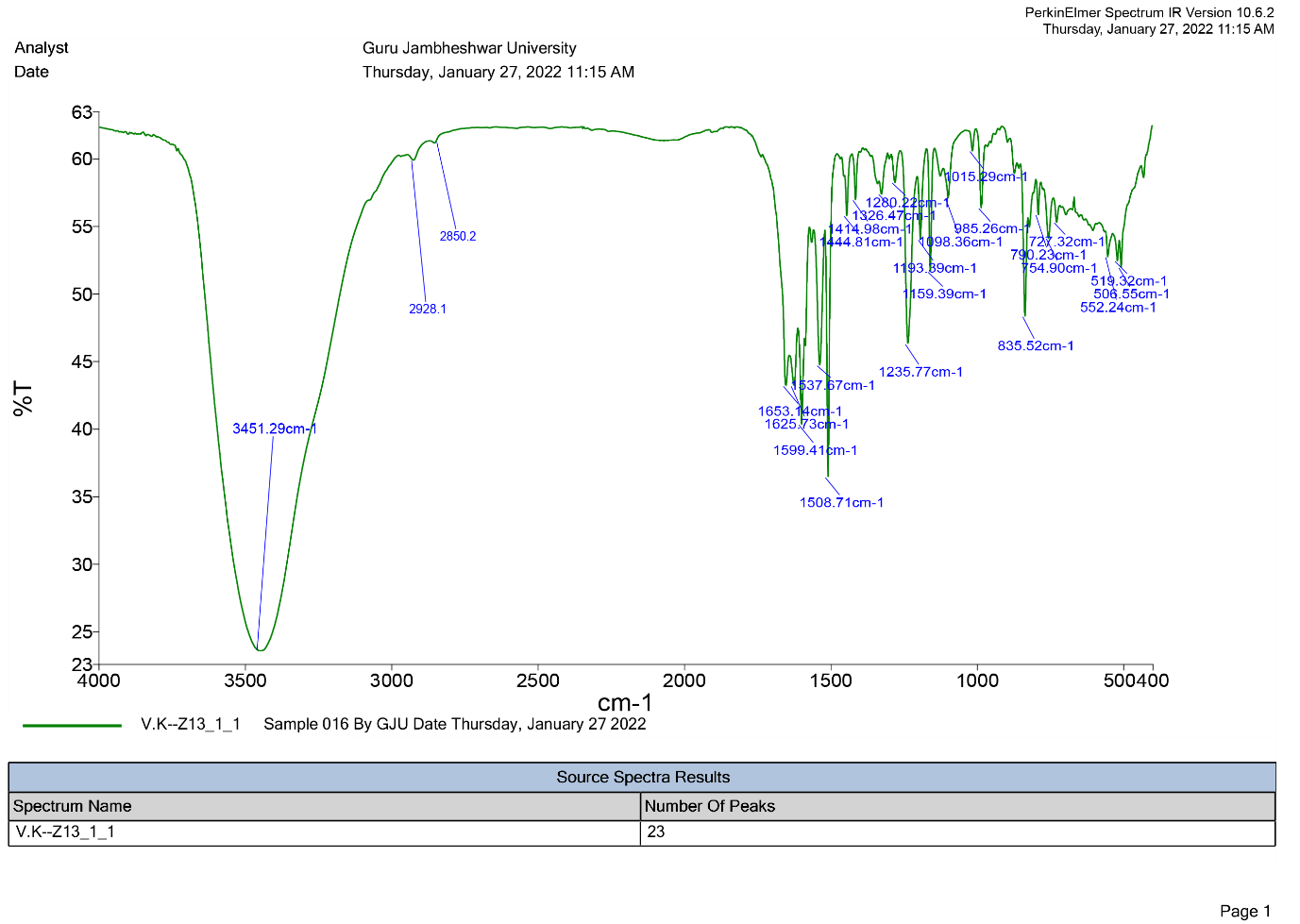

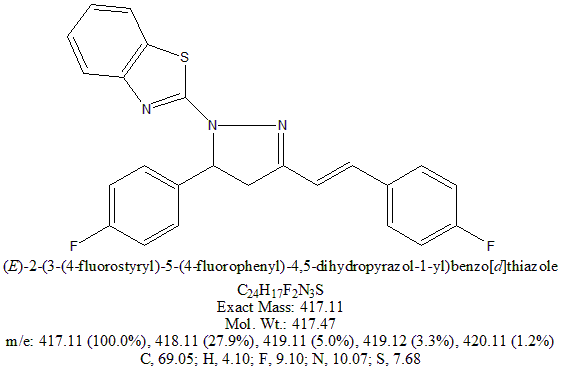


**Figure S21:** IR spectra of compound Z13 [(*E*)-2-(3-(4-fluorostyryl)-5-(4-fluorophenyl)-4,5-dihydropyrazol-1-yl)benzo[d]thiazole]

**
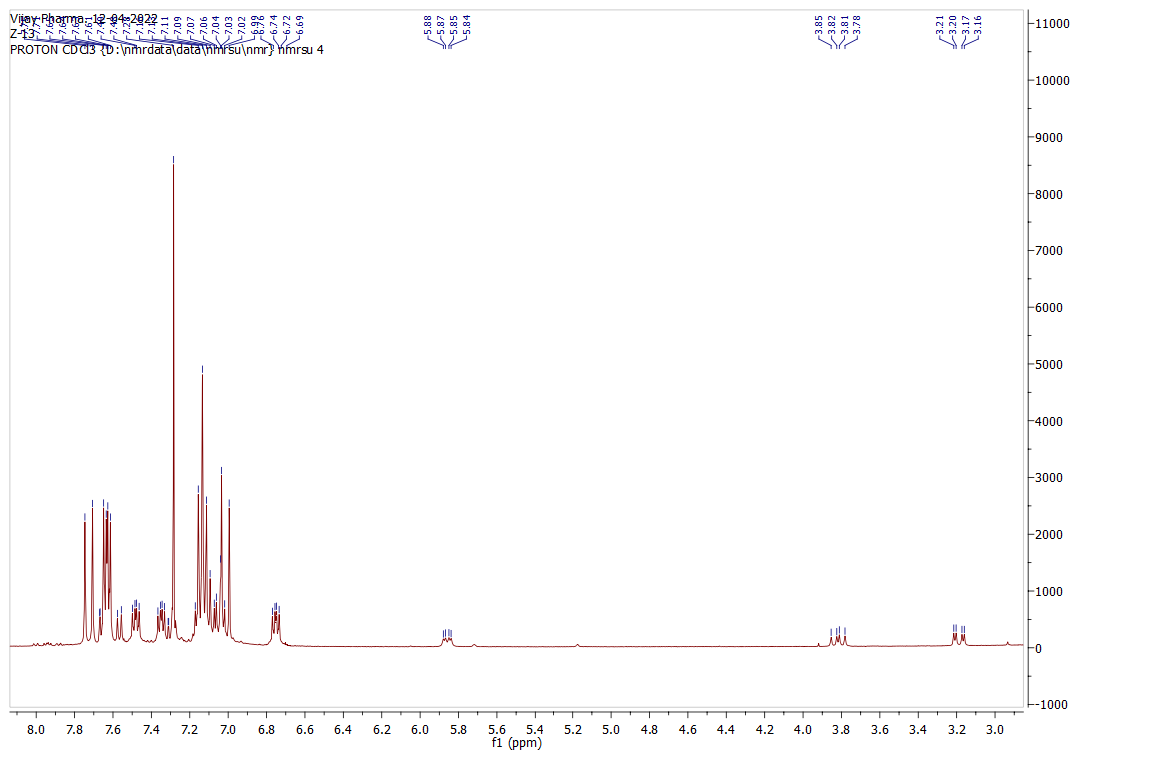
**

**Figure S22:** ^1^H NMR spectra of compound Z13 [(*E*)-2-(3-(4-fluorostyryl)-5-(4-fluoro-phenyl)-4,5-dihydropyrazol-1-yl)benzo[d]thiazole]



**
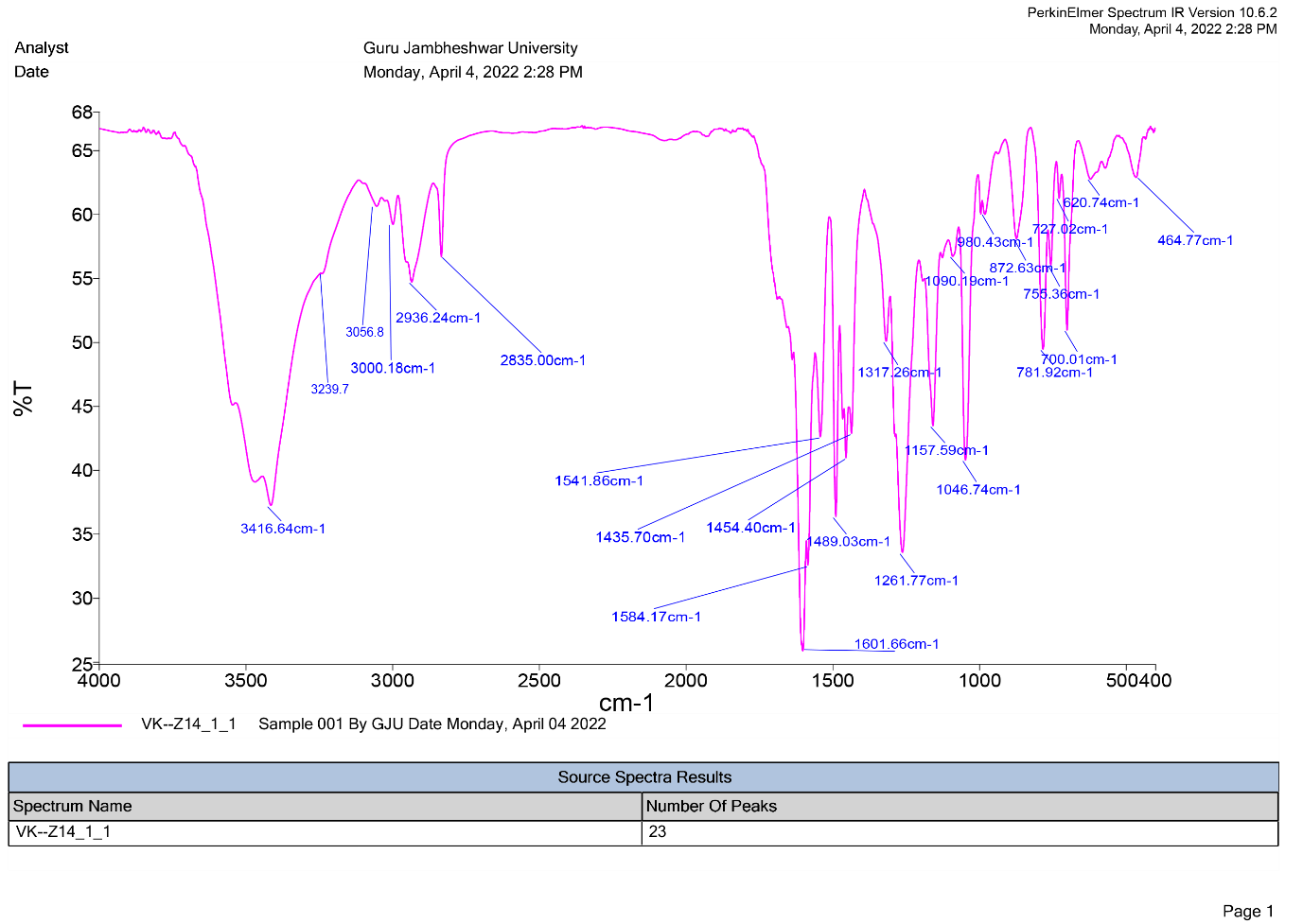
**

**Figure S23:** IR spectra of compound Z14 [(*E*)-2-(3-(3-methoxystyryl)-5-(3-methoxyphenyl)-4,5-dihydropyrazol-1-yl)benzo[d]thiazole]


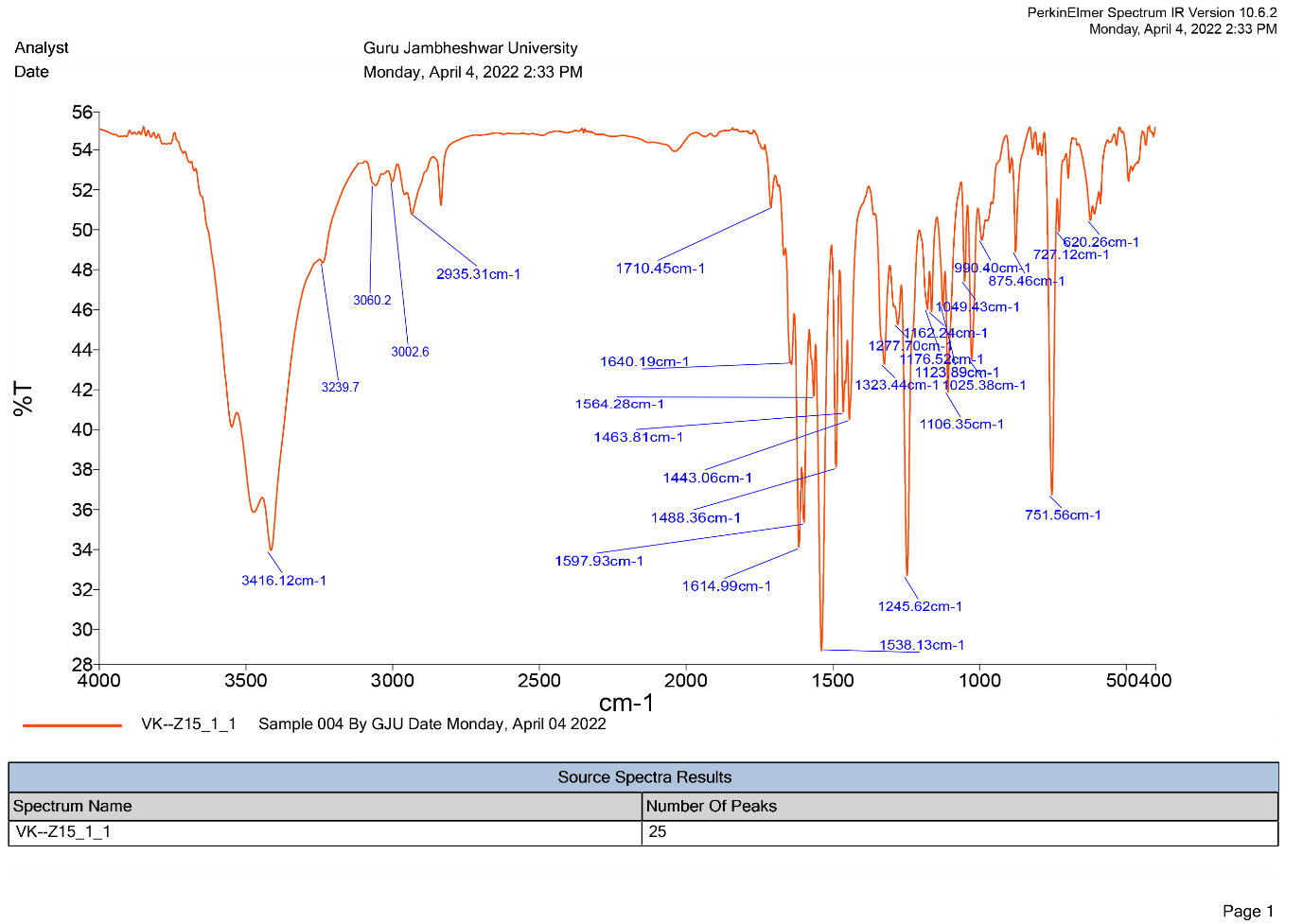

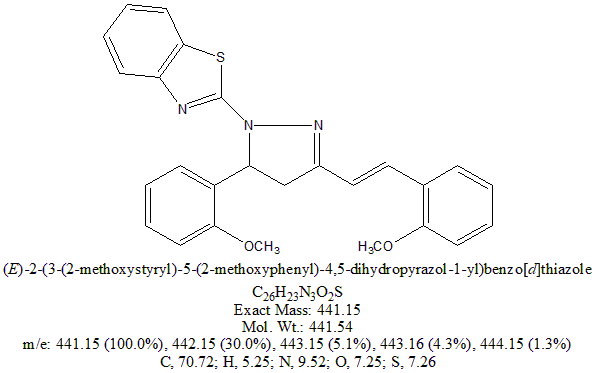


**Figure S24:** IR spectra of compound Z15 [(*E*)-2-(3-(2-methoxystyryl)-5-(2-methoxyphenyl)-4,5-dihydropyrazol-1-yl)benzo[d]-thiazole]


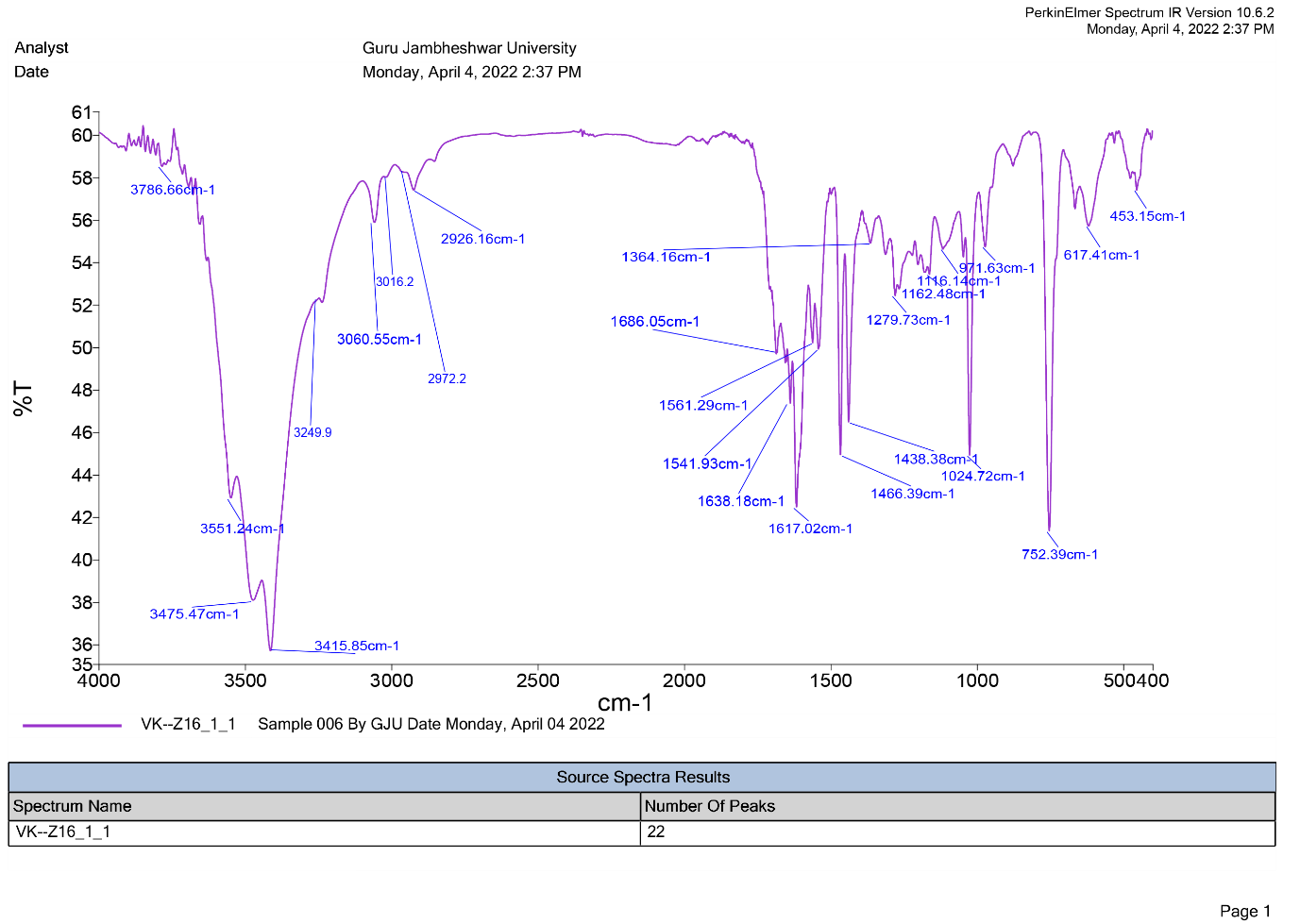

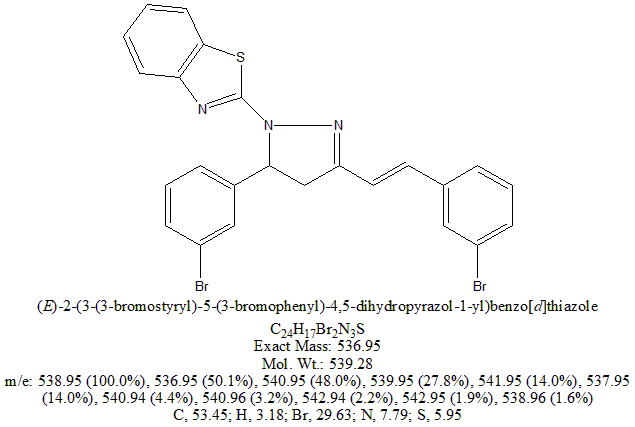


**Figure S25:** IR spectra of compound Z16 [(*E*)-2-(3-(3-bromostyryl)-5-(3-bromophenyl)-4,5-dihydropyrazol-1-yl)benzo[d]thiazole]


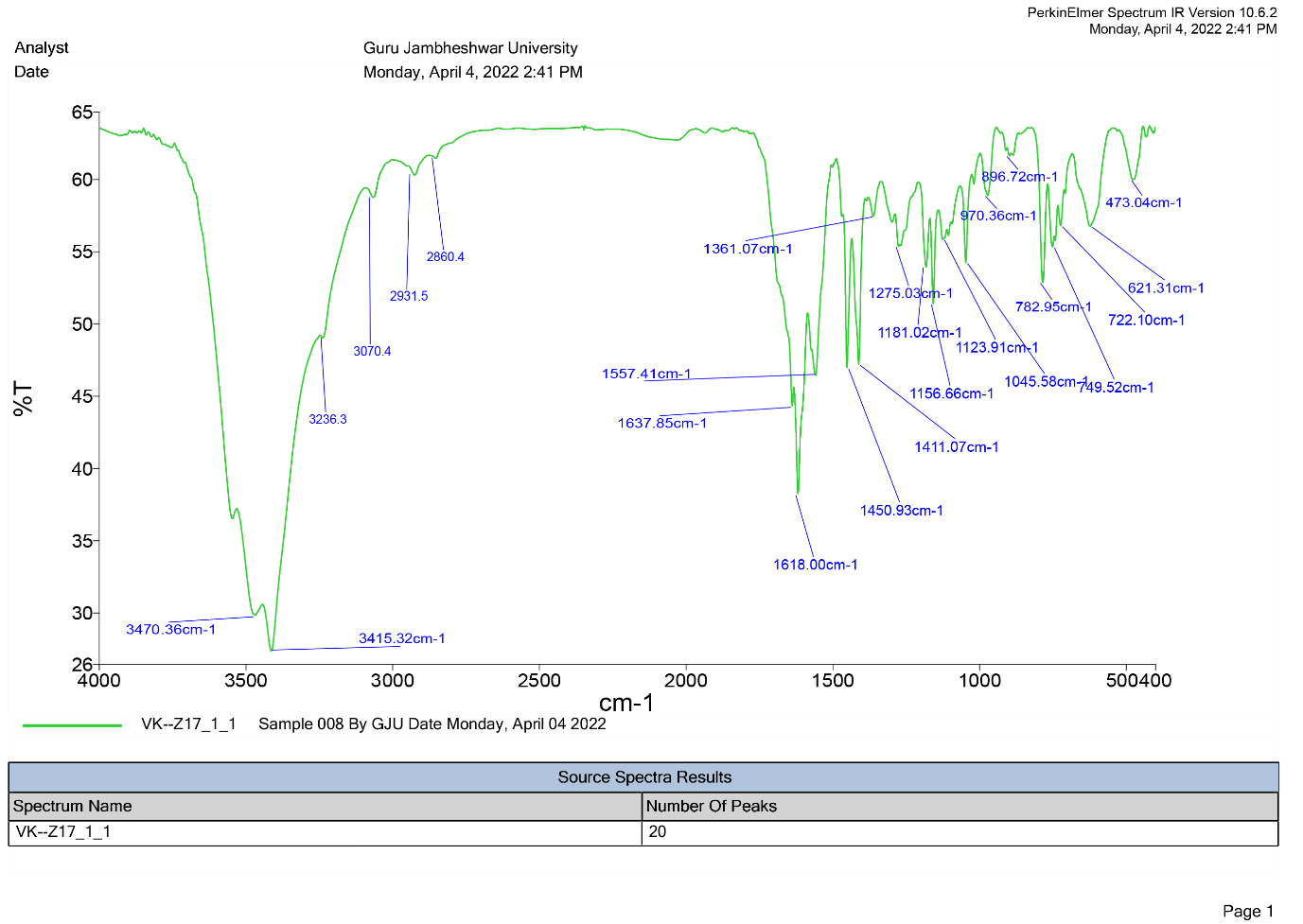

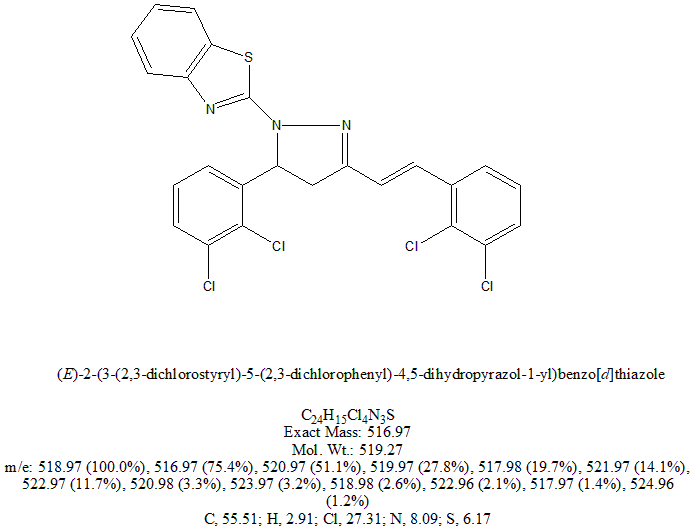


**Figure S26:** IR spectra of compound Z17 [(*E*)-2-(3-(2,3-dichlorostyryl)-5-(2,3-dichlorophenyl)-4,5-dihydropyrazol-1-yl)benzo[d]thiazole]


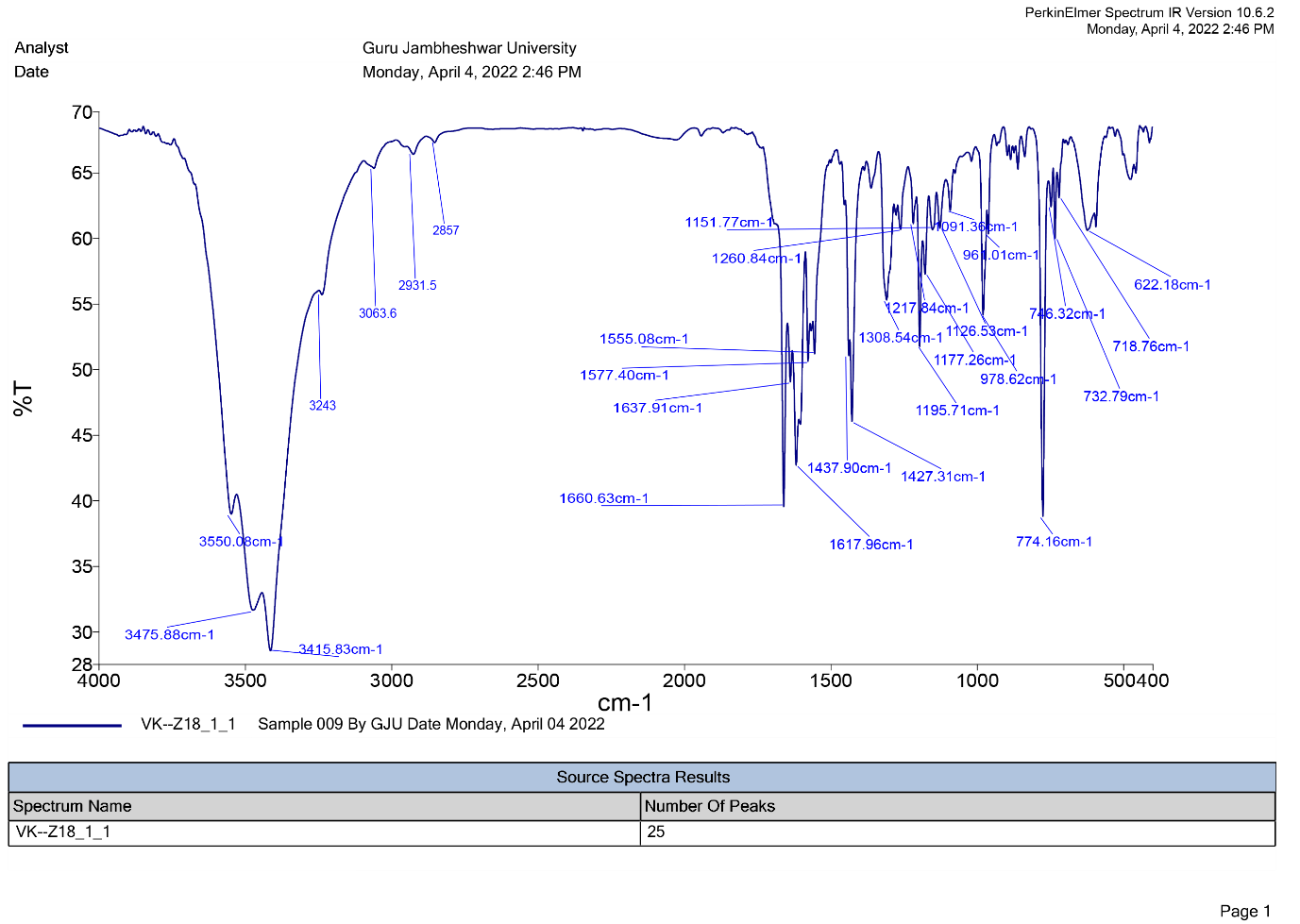

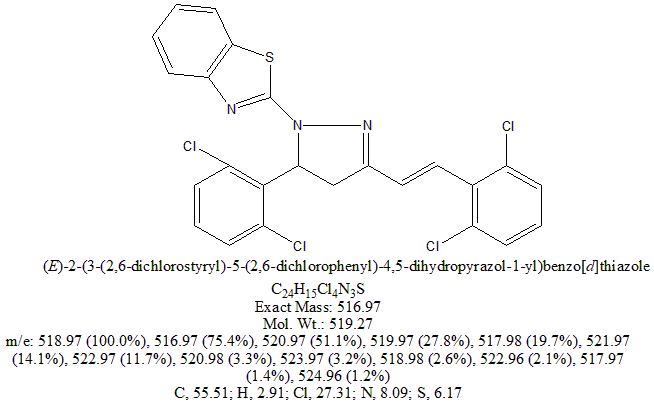


**Figure S27:** IR spectra of compound Z18 [(*E*)-2-(3-(2,6-dichlorostyryl)-5-(2,6-dichlorophenyl)-4,5-dihydropyrazol-1-yl)benzo[d]thiazole]


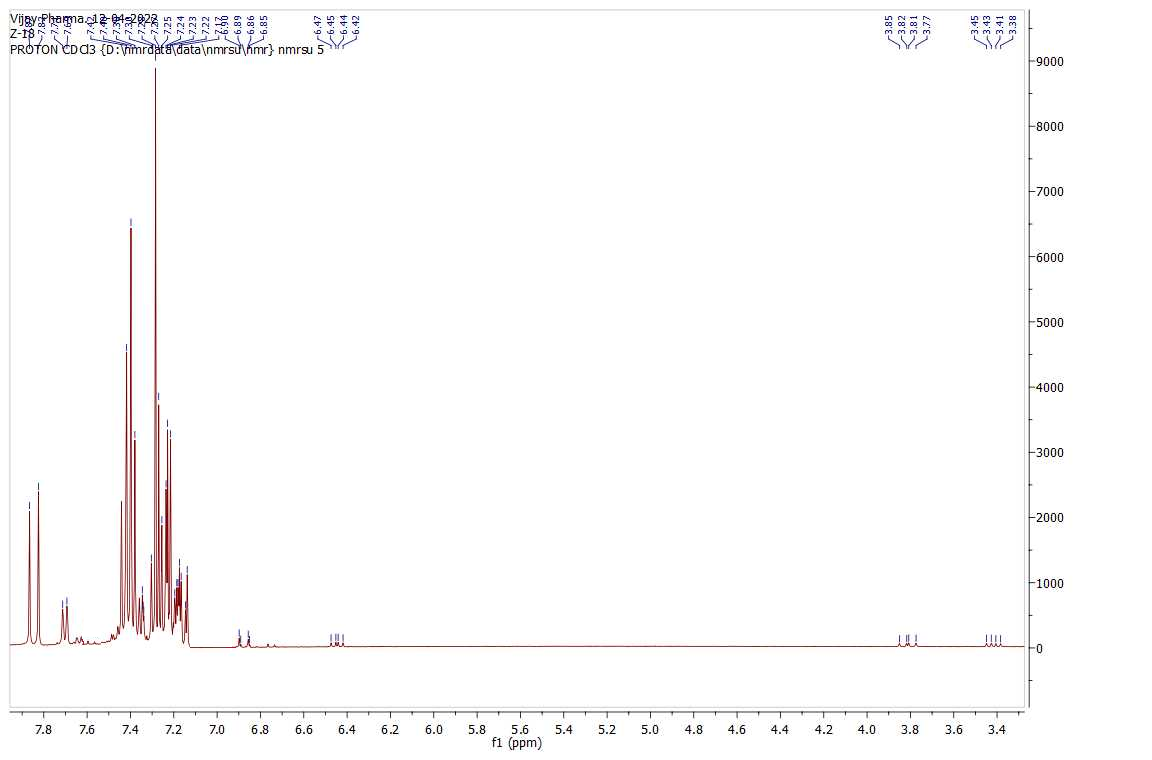


**Figure S28:** ^1^H NMR spectra of compound Z18 [(*E*)-2-(3-(2,6-dichlorostyryl)-5-(2,6-dichlorophenyl)-4,5-dihydropyrazol-1-yl)benzo[d]thiazole]


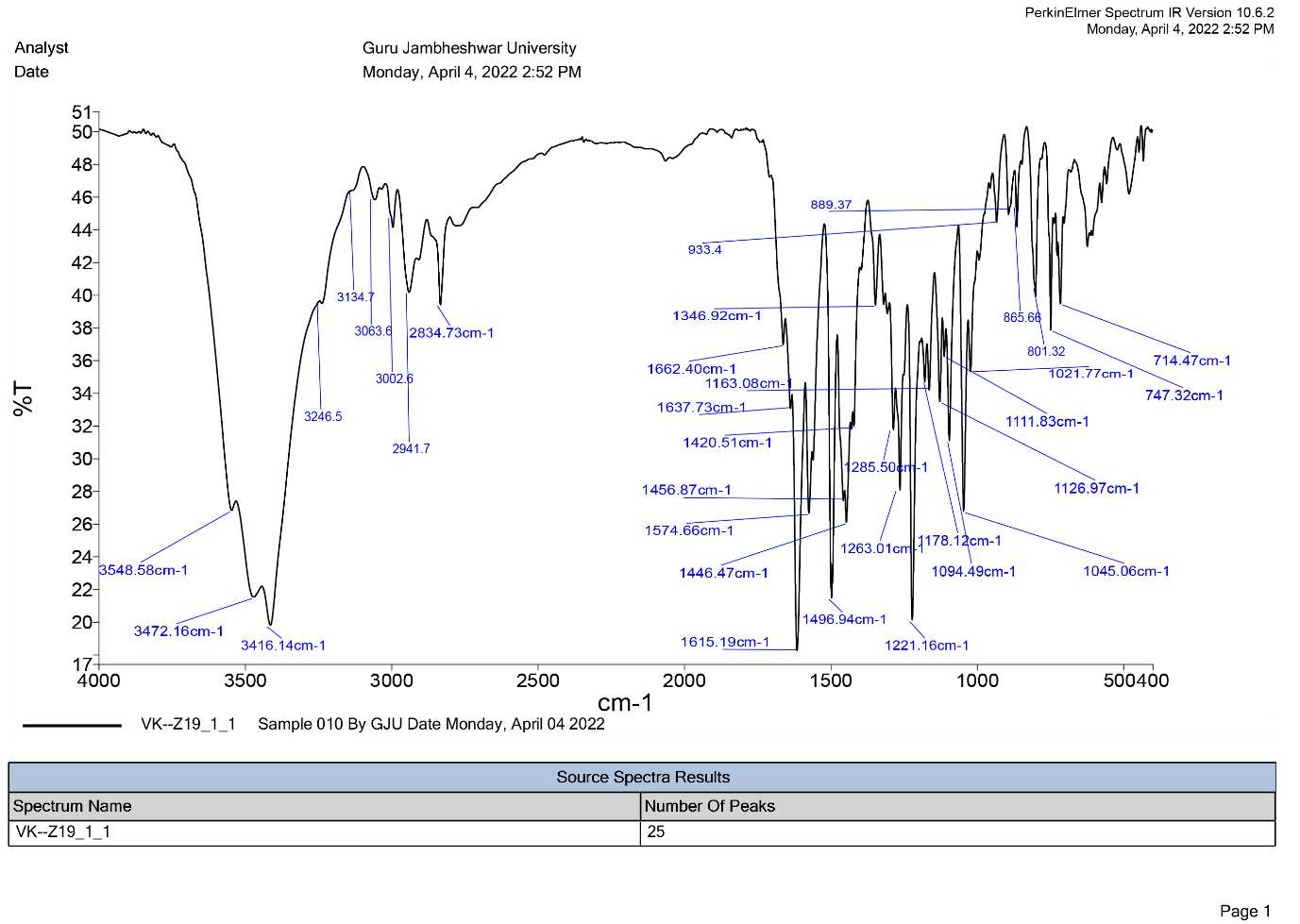

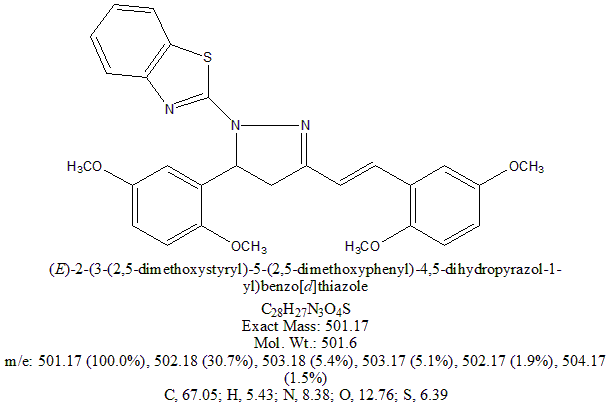


**Figure S29:** IR spectra of compound Z19 [(*E*)-2-(3-(2,5-dimethoxystyryl)-5-(2,5-dimethoxyphenyl)-4,5-dihydropyrazol-1-yl) benzo[d]thiazole]


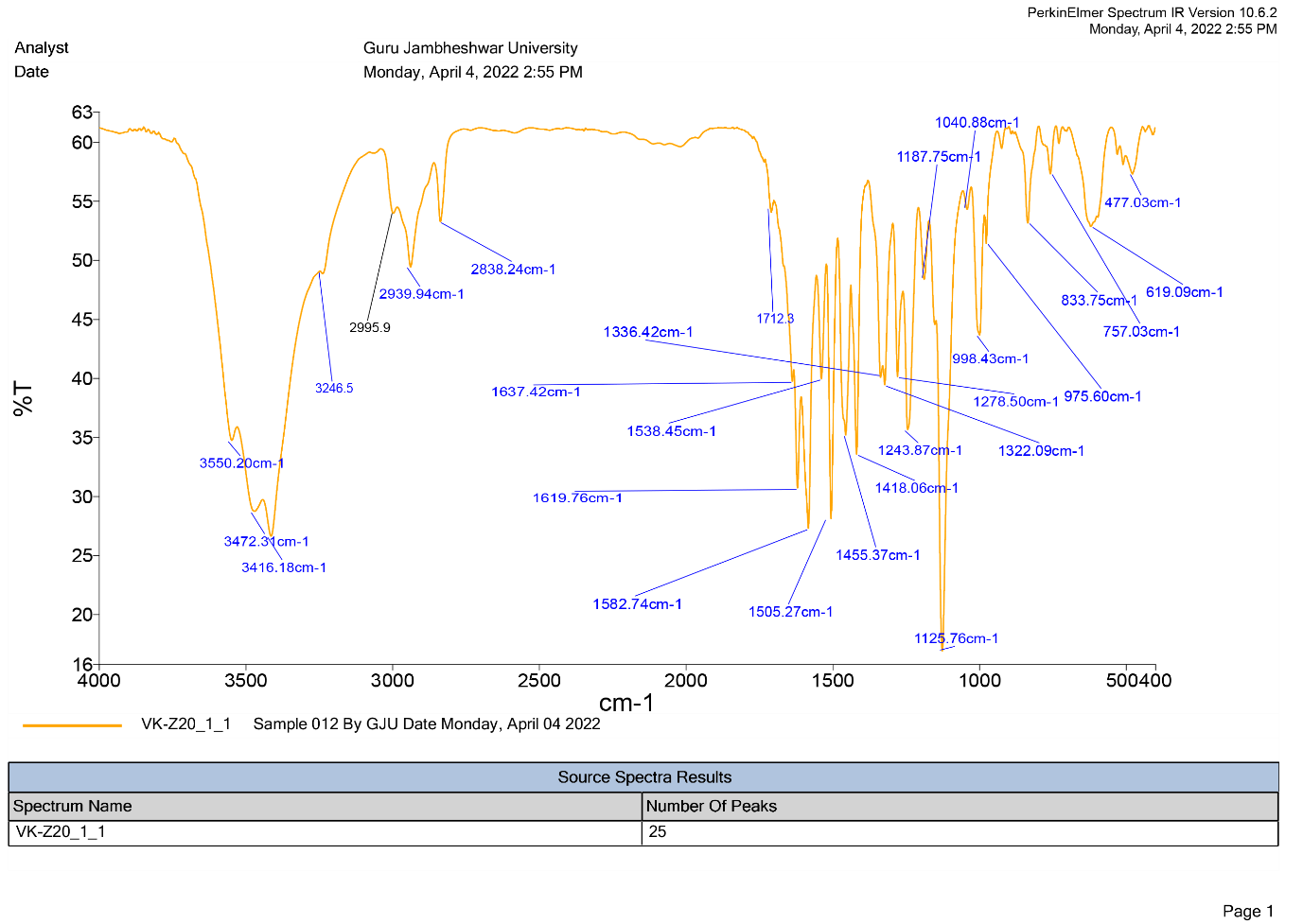

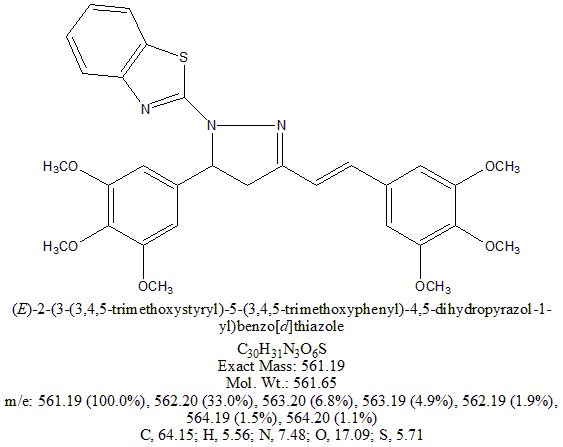


**Figure S30:** IR spectra of compound Z20 [(*E*)-2-(3-(3,4,5-trimethoxystyryl)-5-(3,4,5-trimethoxyphenyl)-4,5-dihydropyrazol-1-yl) benzo[d]thiazole]

**
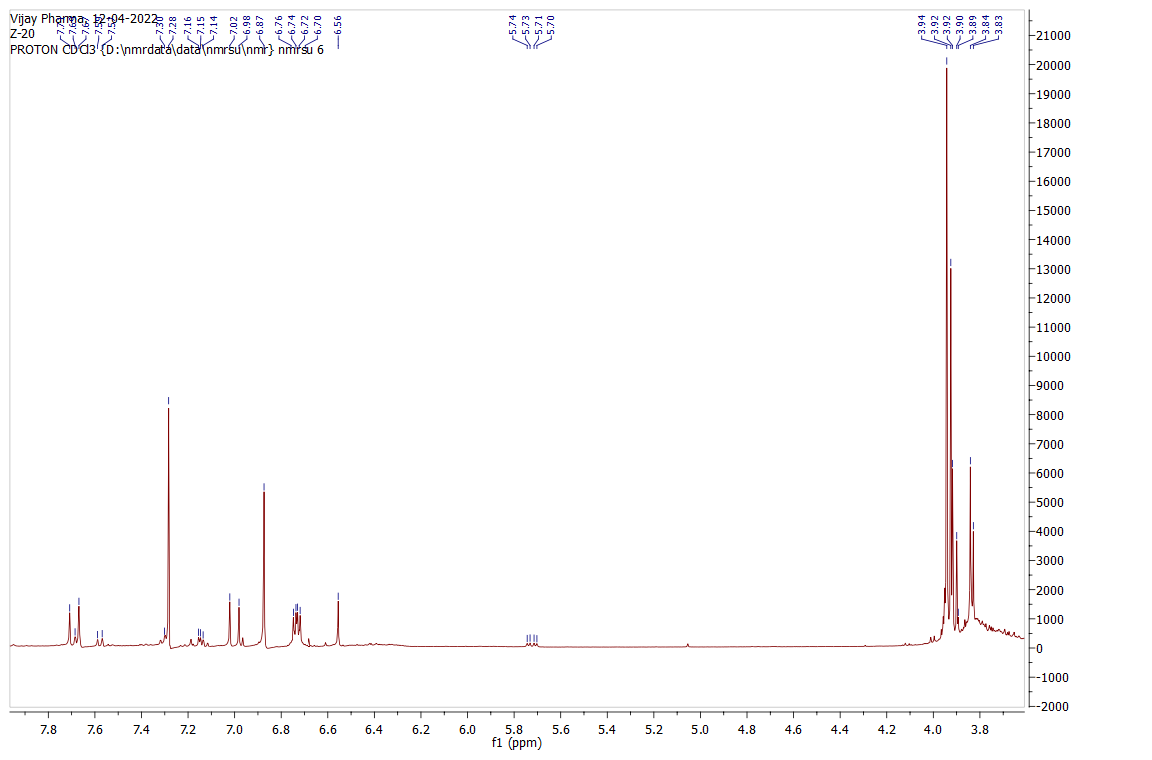
**

**Figure S31:** ^1^H NMR spectra of compound Z20 [(*E*)-2-(3-(3,4,5-trimethoxystyryl)-5-(3,4,5-trimethoxyphenyl)-4,5-dihydropyrazol-1-yl) benzo[d]thiazole]
